# Electrochemical Ammoxidation of Unprotected Glycosides

**DOI:** 10.1021/acselectrochem.5c00111

**Published:** 2025-05-15

**Authors:** Imke M. A. Bartels, Md Asmaul Hoque, J. Prathap Kaniraj, Mathew R. Johnson, Sebastian B. Beil, Shannon S. Stahl, Martin D. Witte, Adriaan J. Minnaard

**Affiliations:** † Stratingh Institute for Chemistry, 3647 University of Groningen, Nijenborgh 7, 9747 AG Groningen, The Netherlands; ‡ Department of Chemistry, 5228 University of Wisconsin−Madison, Madison, Wisconsin 53706, United States; § 28313 Max-Planck-Institute for Chemical Energy Conversion, Stiftstraße 34-36, 45470 Mülheim an der Ruhr, Germany

**Keywords:** Electrocatalytic oxidation, TEMPO, carbohydrates, nitriles, ammoxidation

## Abstract

Cyano-sugars are useful carbohydrate building blocks. Here, we report an electrocatalytic method to oxidize the primary alcohols of unprotected glycosides to the corresponding nitrile using 2,2,6,6-tetramethylpiperidin-1-oxyl (TEMPO) as a mediator and ammonium tetrafluoroborate as the ammonia source. Monosaccharides and disaccharides undergo successful reactions in an acetonitrile/pyridine solvent mixture at room temperature under constant current electrolysis.

## Graphical abstract

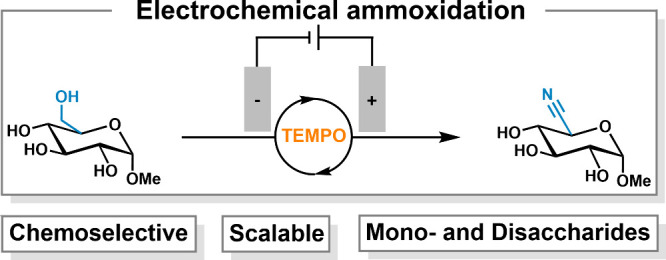

## Introduction

Functionalized carbohydrates are versatile building blocks in a range of industrial applications, including materials^1^ and life sciences.^2–4^ Their preparation is synthetically challenging; however, it involves discriminating between the multiple different hydroxy groups in carbohydrates. The hydroxyl groups have inherent reactivity differences that have been exploited in protecting group strategies.^5–13^ The use of these small reactivity differences for the selective modification of unprotected carbohydrates is rapidly gaining attention as well.^5,6^ Recent efforts have focused on the functionalization of secondary alcohols,^7–10^ epimerization,^11–13^ and carbon skeleton modification.^14,15^

Methods that functionalize the primary hydroxyl group have been known for much longer and are commonly applied to functionalize saccharides^16^ using stoichiometric chemical oxidation or under electrochemical conditions.^17–19^ In the early 1990s, a patent by Casciani et al.^20^ showed that 2,2,6,6-tetramethylpiperidin-1-oxyl (TEMPO) site-selectively oxidizes the primary hydroxyl group of alkyl polyglucosides into the carboxylic acid in the presence of unprotected secondary hydroxyl groups. Later, Davis and Flitsch reported the selective oxidation of partially protected monosaccharide derivatives using TEMPO.^21^ Sugar aldehydes in water mainly exist in the hydrate form and undergo rapid oxidation to the carboxylic acid.^22–24^ The aldehyde product can be obtained when the reaction is carried out in the absence of water.^22^

Oxidation of the primary alcohol or aldehyde in sugars to the amide or the nitrile using ammonia, so-called ammoxidation, has received much less attention.^25,26^ Cyano-sugars, or sugar nitriles, are useful building blocks, as the nitrile-group can be converted into the corresponding amine and various nitrogen containing heterocycles, including oxazoles^27^ and tetrazoles.^28^ Cyano-sugars are also interesting as target molecules for click-chemistry^29^ and as glycosidase inhibitors.^30^

The conventional synthesis of nitriles employs nucleophilic substitution with cyanide, the dehydration of amides^31^ and oximes,^32,33^ or the oxidation of amines.^34^ The introduction of a cyano group at the C5-position of pyranoses has been achieved by protection of the secondary hydroxy groups, oxidation of the primary hydroxy group to the aldehyde, aldoxime formation and dehydration.^35^ A preferred approach would directly introduce the cyano group in carbohydrates without the need for protecting groups, and ammoxidation has been shown to be compatible with unprotected glycosides given that oxidation is site-selective. For example, Vatèle demonstrated that utilizing a (TEMPO)/phenyliodine­(III) diacetate (PIDA)/NH_4_OAc system, a partially protected carbohydrate could be converted to the nitrile.^25^ Recently, we have shown that a modified procedure may be used with unprotected carbohydrates,^26^ (Figure 1A), albeit using 50% water as co-solvent and requiring a large excess of NH_4_OAc to suppress competing formation of the carboxylic acid.

**Figure 1:**
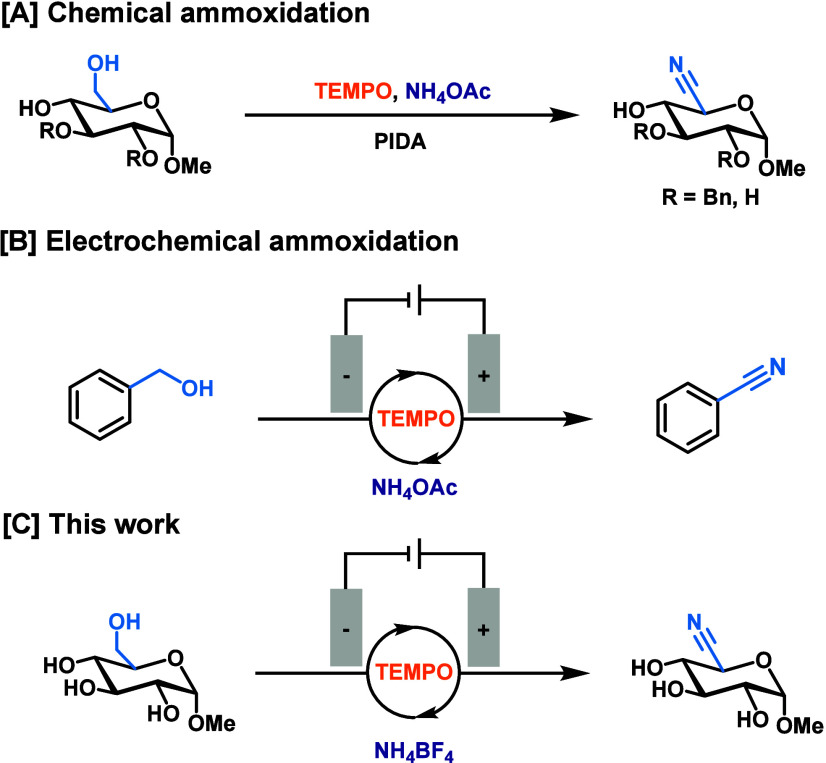
Context and strategy for the ammoxidation of carbohydrates.

The use of PIDA is not atom economical. In our previous studies, 6.2 g of PIDA was used to oxidize 1.5 g of glucoside. The use of electrochemistry instead of chemical oxidants to regenerate TEMPO could mitigate this issue. Several methods have been developed for the electrochemical ammoxidation of benzyl alcohols and benzaldehydes. For example, in 1988, Chiba et al.^36^ reported that benzaldehydes can be oxidized electrochemically to the corresponding nitriles using KI and ammonia. Other electrochemical ammoxidation methods have been reported using different redox catalysts and ammonia sources.^37^ The first TEMPO-mediated electrochemical ammoxidation was developed in 2016 by the group of Li,^38^ using hexamethyl disilazane as an ammonia equivalent in the conversion of benzaldehyde, and later benzyl alcohol,^39^ into the corresponding nitrile. This approach was also adapted to use NH_4_OAc as the ammonia source^40^ (Figure 1B).

The electrochemical ammoxidation of aliphatic hydroxyl groups and aldehydes is more challenging. In the few instances where such substrates were successfully converted to nitriles, the reactions typically suffered from lower yields and reduced charge efficiency.^36^ Furthermore, some substrates were susceptible to side reactions, such as aldol condensations of the aldehyde intermediates.^39^

The previously reported TEMPO-mediated ammoxidation of glycopyranosides using stoichiometric PIDA and the electrochemical ammoxidation of benzyl alcohol raised the possibility of TEMPO-mediated electrochemical ammoxidation of unprotected carbohydrates (Figure 1C). The poor solubility of unprotected carbohydrates in standard organic solvents presents a challenge as the use of water to aid solubility can contribute to the formation of carboxylic acid products. This side reaction must be suppressed to enable electrochemical ammoxidation. Herein, we address this challenge and report a method for the selective electrochemical ammoxidation of unprotected glycosides.

## Results and Discussion

Partially protected glucopyranoside **1** is soluble in acetonitrile, a solvent commonly used in electrochemical reactions, and it is a suitable substrate for TEMPO/PIDA ammoxidation.^25^ Therefore, our initial tests with **1** used the electrochemical conditions of Li et al.,^40^ with TEMPO as the mediator, ammonium acetate as the ammonia source, and sodium perchlorate as the supporting electrolyte, in acetonitrile. The ammonium acetate did not fully dissolve in acetonitrile, even after extended stirring. Several minutes after the reaction was initiated, a precipitate formed and the reaction lost conductivity. The same problem occurred with all other supporting electrolytes tested in neat acetonitrile. The addition of 10 vol % water as a co-solvent helped to solubilize the ammonium acetate in the reaction mixture, allowing it to be used as both the ammonia source and the supporting electrolyte. The reaction mixture is biphasic, however, likely reflecting the kosmotropic properties of ammonium acetate, which affects the hydrogen bonding of water and causes phase separation. Nonetheless, after some optimization (Figure S4), we were able to achieve full conversion of **1** and formation of the desired nitrile **2** as the major product (Figure 2A).

**Figure 2:**
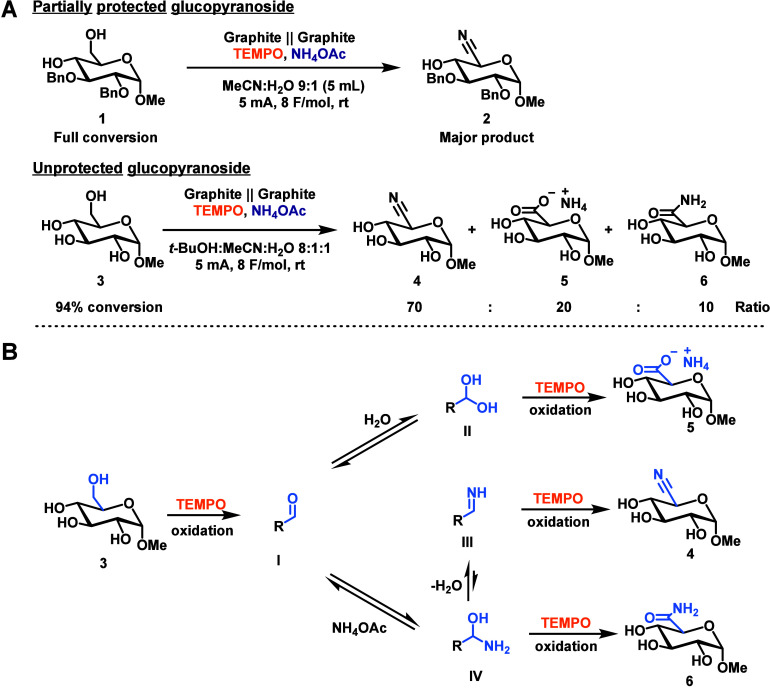
A) Electrochemical oxidation of methyl-2,3-di-*O*-benzyl-α-d-glucopyranoside **1** and methyl-α-d-glucopyranoside **3** using an IKA Electrasyn 2.0. B) Potential reaction pathways.

These promising initial results prompted an exploration of the electrochemical ammoxidation of unprotected carbohydrates. These substrates are much more polar than **1** and do not dissolve well in acetonitrile, so the above conditions using water as a co-solvent were tested. Electrochemical ammoxidation of glucoside **3** to the desired nitrile **4** did occur, but formation of **4** was accompanied by the ammonium salt of glucuronic acid **5** and amide **6** as major and minor side products, respectively (Figure 2A). No epimerization was observed, contrasting the PIDA-promoted ammoxidation of **3** reported by Haaksma et al.^26^ The product ratios and yields obtained from the electrochemical ammoxidation greatly varied between reactions and appeared to depend strongly on the mixing efficiency. Previous work^26,41^ suggested that the ammoxidation reaction occurs preferentially in the organic phase, while oxidation to the acid **5** occurs in the aqueous phase. If correct, inefficient mixing of the biphasic solvent system likely contributes to poor reproducibility.

Most other solvent mixtures tested either did not dissolve all the reaction components or did not show suitable conductivity to support electrochemistry (see Table S1 for details). One exception was *t*-BuOH:MeCN:water in a 8:1:1 ratio (Table S1 entry 12). This mixture was homogeneous throughout the reaction, maintained a conductivity of 280 μS, and reproducibly yielded the nitrile. These conditions still led to formation of side-products **5** and **6**, however, likely reflecting the presence of H_2_O in the solvent mixture, which contribute to side products through the mechanisms shown in Figure 2B.

Increasing the NH_4_OAc concentration suppressed the side-product formation; however, a lower conversion of **3** was observed (Table S4). Cyclic voltammetry (CV) studies were conducted to investigate the effect of NH_4_OAc on TEMPO under these conditions. The CV of TEMPO in the presence of NH_4_OAc exhibits an irreversible redox feature (Figure S5), implicating among others rapid decomposition of TEMPO^+^. Additional contributing factors, such as the solution’s alkalinity and conductivity, may also influence the formation of the non-peak-shaped voltammogram. This result may be compared with previous studies of benzyl alcohol and NH_4_OAc, which have shown good TEMPO-mediated ammoxidation reactivity,^40,42^ presumably because TEMPO^+^ reacts rapidly with the benzylic alcohol. This contrast suggests that carbohydrates may not interact efficiently with TEMPO^+^, leading to slower oxidation and competing TEMPO^+^ degradation.

The above consideration motivated efforts to identify a new solvent system and ammonia source. CV studies evaluated the redox behavior of various ammonium salts (NH_4_OAc, NH_4_BF_4_, NH_4_PF_6_, NH_4_OH, (NH_4_)_2_CO_3_, and NH_4_HCO_2_) in different solvents by cyclic voltammetry in the absence and presence of TEMPO and substrate (see section 6 in the Supporting Information). TEMPO displayed electrochemical reversibility in the presence of NH_4_BF_4_ and NH_4_PF_6_ in 8:1:1 *t*-BuOH:MeCN:water and in 10 vol % pyridine in acetonitrile (Figure 3B); however, the ammoxidation reaction did not proceed well under the former conditions. The outcome is rationalized by the higher acidity of HBF_4_ and HPF_6_ relative to that of acetic acid. This feature will reduce the concentration of ammonia relative to conditions with NH_4_OAc and hinder formation of the imine intermediate **III** (cf. Figure 2B). Strong acid can also inhibit the electrochemical oxidation of the reduced TEMPOH form of the mediator^43–45^ and thereby limit catalytic turnover (Figure 3A). We postulated that these issues would be addressed by using pyridine in acetonitrile as a basic buffer under these modified conditions, and screening of different conditions (Table S7) showed that 10 vol % pyridine in acetonitrile could attenuate the acidity of the medium and aid in solubilization of the carbohydrate substrate in the absence of water.

**Figure 3:**
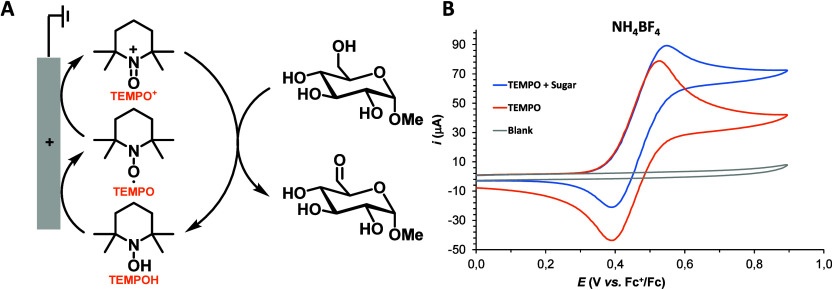
A) TEMPO-catalyzed electrochemical oxidation of methyl-α-d-glucopyranoside.^44^ B) CVs (100 mV/s) in MeCN:pyridine 9:1 and NH_4_BF_4_ (0.3 M) of TEMPO (2 mM) (red trace); TEMPO (2 mM) and methyl-α-d-glucopyranoside (100 mM) (blue trace). All potentials are reported relative to those of Fc^+^/Fc.

TEMPO still exhibits a quasi-reversible redox feature at *E*_1/2_ = 540 mV in the presence of ammonium tetrafluoroborate (Figure 3B, orange trace). CV analysis of a solution containing ammonium tetrafluoroborate, TEMPO, and methyl-α-d-glucopyranoside **3** reveals a slight increase in anodic current at the TEMPO redox potential, with a corresponding decrease in the cathodic feature (Figure 3B, blue trace). The partial reversibility of the TEMPO/TEMPO^+^ redox couple in the presence of **3** shows that oxidation of the sugar is slower than the time scale of the CV scan under these conditions, contrasting the electrocatalytic behavior often observed for TEMPO-mediated alcohol oxidation.^46^

Efforts then shifted to bulk electrolysis to explore the preparative conditions for substrate ammoxidation. Initially, reactions were conducted with TEMPO as the mediator, ammonium tetrafluoroborate as the ammonia source and the supporting electrolyte, and acetonitrile:pyridine 9:1 as the solvent. Electrolysis was conducted by using constant current electrolysis with a graphite rod anode and platinum wire cathode. Evaluation of the reaction of **3** showed that complete conversion required passing 8 F/mol of charge. These conditions resulted in 96% conversion of **3**, but the yield of the desired product **4** was only 28% due to product decomposition (Table 1, entry 1). Replacing TEMPO by 4-NAc-TEMPO or ABNO led to even lower yields of the desired nitrile (Table 1, entries 2 and 3). The mediator appeared to decompose rapidly during the reaction, as indicated by a significant increase in potential early in the reaction (Figure S23). Lowering the TEMPO loading led to significantly lower conversion (Table 1, entry 4), while increasing the TEMPO loading to 30 mol % led to complete conversion of **3** and an 80% yield of **4** (Table 1, entry 5). Use of other ammonium salts had a deleterious effect, consistent with observations from the CV studies noted above (Table 1, entries 8–11). Decreasing the quantity of the ammonium salt from 3 to 2 equiv retained good performance (Table 1, entry 13). Adjusting the applied constant current to 10 mA led to further improvement, accessing **4** in 93% yield (Table 1, entry 15) and decreasing the reaction time from 14 to 5.6 h. Further variations to the reaction conditions, including temperature and electrode material, had little impact or significantly diminished product yields (see sections 5.4.1 and 5.4.2 in the Supporting Information for details). When no TEMPO was added or no current was passed, quantitative substrate was recovered (Table 1, entries 6 and 7).

**Table 1:**
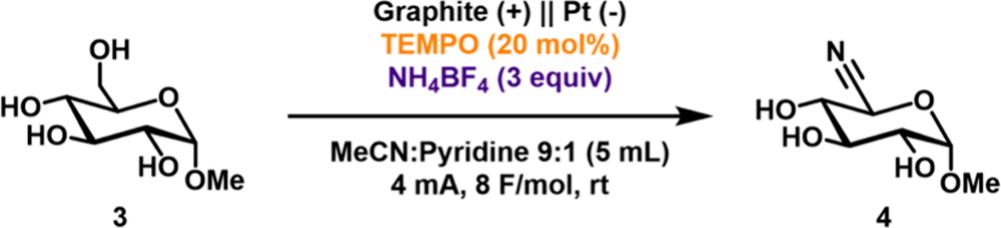
Optimization of the Bulk Electrolysis of **3**

Entry	Deviation standard conditions	Conversion (%)	Yield **4** (%)^a^
1		96	28
2	4-NAc-TEMPO	49	6
3	ABNO	37	2
4	10 mol % TEMPO	63	24
5	30 mol % TEMPO	100	80
6^c^	0 mol % TEMPO	0	0
7	No electrolysis	0	0
8^b^	NH_4_PF_6_ instead of NH_4_BF_4_	100	87
9^b^	NH_4_OAc instead of NH_4_BF_4_	48	14
10^b^	NH_4_OHinstead of NH_4_BF_4_	100	38
11^b^	NH_4_HCO_3_ instead of NH_4_BF_4_	41	10
12^b^	1.2 equiv NH_4_BF_4_	100	80
13^b^	2 equiv NH_4_BF_4_	100	84
14^b^	4 equiv NH_4_BF_4_	100	75
15^c^	**10 mA**	100	93
16^c^	15 mA	95	86

a NMR yields with trimethoxybenzene as internal standard.

b Standard conditions using 30 mol % TEMPO.

c Standard conditions using 30 mol % TEMPO, 6 F/mol, and 2 equiv NH_4_BF_4_.

Analysis of the constant current electrolysis profile (Figure 4) in combination with quantitative NMR (qNMR) spectroscopy provided valuable insights into the reaction. With 30 mol % TEMPO, the anode maintained a steady potential of 550 mV (vs FcH^+^/FcH) over the first 6 F/mol of applied charge, and substrate **3** fully converted into the desired nitrile **4** (Figure 4). After this point, the electrolysis potential was increased and product decomposition was observed. When using less TEMPO, less charge passed before the potential jump occurred and lower conversion of **3** and incomplete mass balance was observed. These electrolysis profiles suggest that TEMPO decomposes during the reaction, the potential increases, and the product starts to decompose when TEMPO is no longer available.

**Figure 4:**
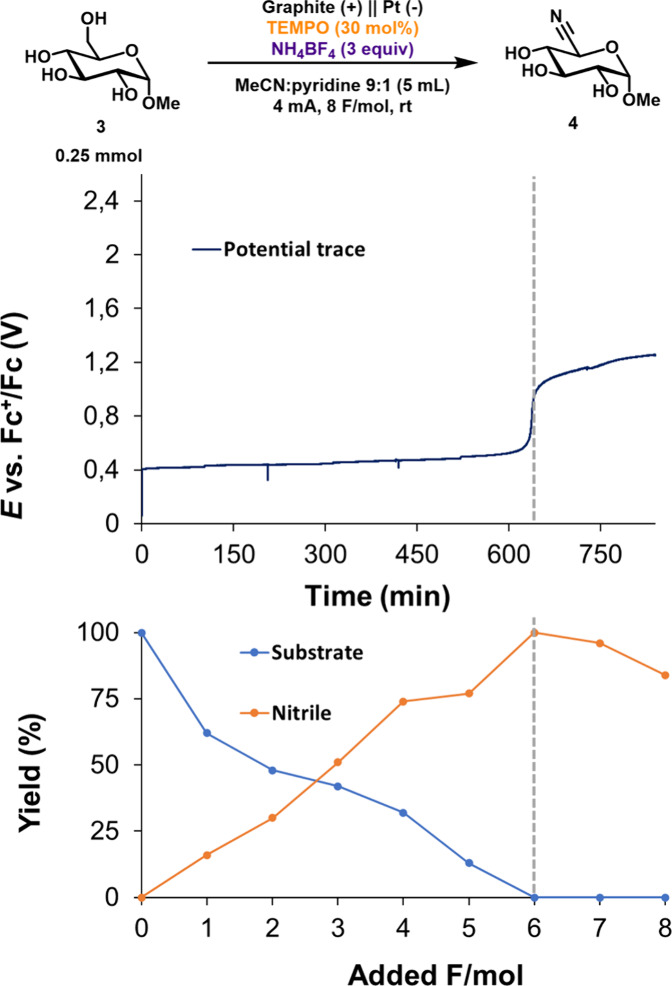
Electrochemical ammoxidation. Top graph: the constant current electrolysis trace. Bottom graph: the qNMR yield over time.

The optimized conditions were then used for the ammoxidation of a representative collection of mono- and disaccharides (Table 2). The results show that the stereochemistry of the anomeric position has little effect on the reaction outcome. As both methyl-β-d-glucopyranoside **4b** and methyl-β-d-galactopyranoside **4e** generated nitriles in 92% and 89% yields, respectively, which is similar to the yields of their α-epimers. Other glucopyranoside epimers, methyl-α-d-mannopyranoside **4c** and methyl-α-d-galactopyranoside **4d**, and methyl-*N*-acetyl-α-d-glucosamine **4f** afforded good yields of the nitrile products. Unprotected sugars can be challenging to isolate, so one of these monosaccharides, methyl-α-d-glucopyranoside **4a**, was acetylated (see the Supporting Information) and isolated with 75% yield, to quantify the difference between the qNMR and isolated yields (see the Supporting Information).

**Table 2:**
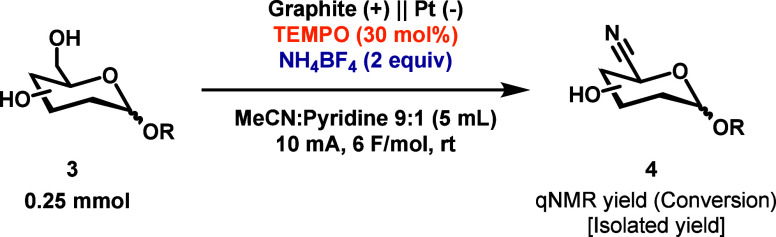

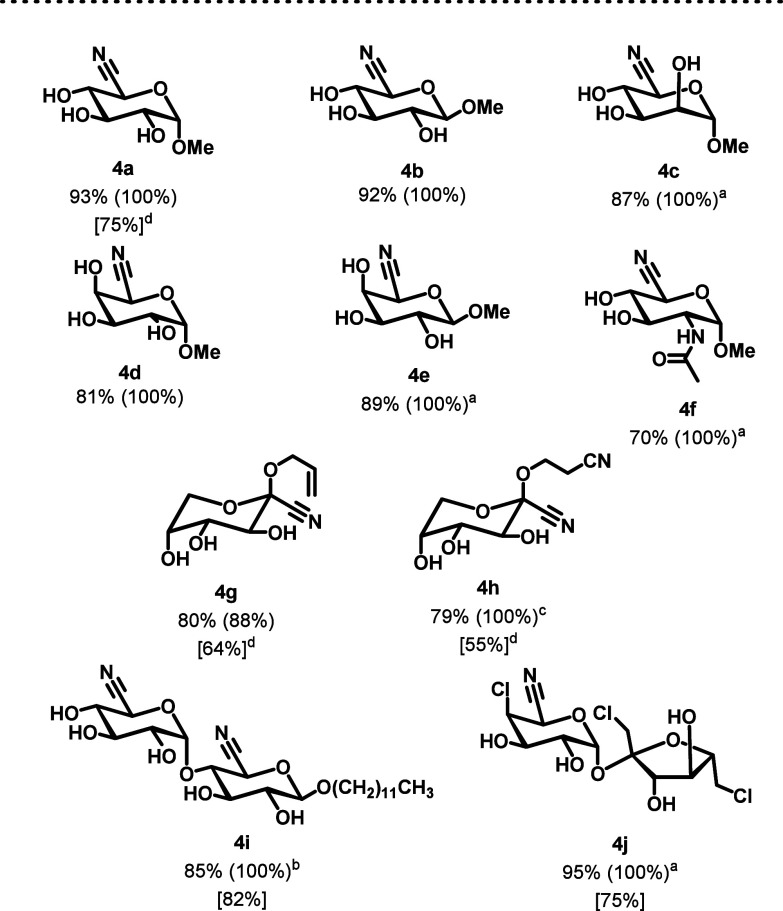
Substrate Scope for the Electrochemical Ammoxidation of Monosaccharides^a^

a 8 F/mol was used.

b 60 mol % TEMPO, 4 equiv NH_4_BF_4_, 5 mA and 12 F/mol were used.

c 5 mA was used.

d Isolated as the tri-acetate.

e Yield (conversion): determined by quantitative ^1^H NMR (400 MHz, CD_3_OD) using trimethoxybenzene or dimethyl sulfone as internal standard.

The method was also successfully used to oxidize the fructopyranosides allyl β-d-fructopyranoside **4g** (80% yield) and cyanoethyl-β-fructopyranoside **4h** (79% yield). These new substrates were also tested with the conventional oxidation method from Haaksma et al.^47^ using TEMPO/PIDA, but both failed to react. Due to the lower solubility of cyanoethyl-β-fructopyranoside **4h**, the applied current was lowered to 5 mA for this reaction. As a high applied current with poorly soluble substrates led to lower yields.

Disaccharides also proved to be effective substrates for electrochemical ammoxidation as long as they have adequate solubility. *n*-Dodecyl β-d-maltoside **4i** underwent oxidation to the corresponding dicyano species in a high yield (85%). A two-fold increase in the catalyst/reagent loading was used in this case (60 mol % loading of TEMPO, 4 equiv of NH_4_BF_4_, 12 F/mol) to account for the presence of two primary hydroxyl groups, and lower constant current was applied (5 mA) due to poor substrate solubility. Finally, sucralose **4j** was shown to generate nitrile in 95% yield. Attempts to subject penta- and hexafuranosides to these ammoxidation conditions met with failure. Non-reducing and free sugars, such as sucrose and glucose, respectively, were not reactive, due to their to low solubility in the solvent mixture.

To demonstrate the scalability of the reaction, **3** was oxidized on 1.5 g scale using a larger batch reactor^48^ containing multiple electrodes (see section 3 in the Supporting Information for details) (Figure 5). Graphite rods were used as the anode, and stainless-steel rods as cathodes. A constant current of 100 mA (1.6 mA/cm^2^) was chosen to achieve an overall cell voltage similar to the cell voltage associated with the small-scale reactions. Performing this reaction led to product **4a** in 92% qNMR yield.

**Figure 5:**
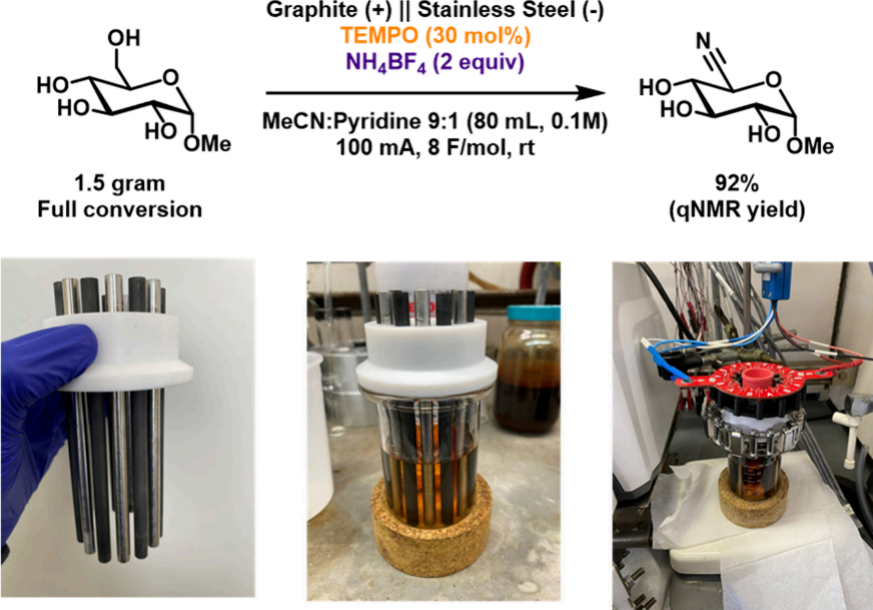
Gram-scale electrochemical ammoxidation in a multi-electrode batch electrolysis reactor.

## Conclusion

The results described herein have led to an electrochemical method for the selective ammoxidation of unprotected carbohydrates. Monosaccharides and disaccharides undergo reaction to afford the corresponding C5-cyano-sugars in good to excellent yields. The use of TEMPO as a mediator allows for the site-selective ammoxidation of the primary hydroxyl group. Ammonium tetrafluoroborate is an effective ammonia source, when paired with pyridine to buffer the acetonitrile reaction medium. These conditions provide the required basic environment and support substrate solubility, enabling effective product formation in the absence of water. Use of low-cost graphite anode and stainless-steel cathode, allows for easy scalability using a multi-electrode stirred batch reactor. Overall, this method achieved excellent chemoselective ammoxidation of the primary alcohol of unprotected sugars to access cyano-sugars.

## Experimental Methods

### General Considerations

All solvents were purchased from commercially available sources and used without further purification. All ammonium salts, carbohydrates, and mediators were purchased form Sigma-Aldrich, TCI, Biosynth and Combi-Blocks. All chemicals were used without further purification. Electrochemical reactions were carried out using either 1) an ElectraSyn 2.0 device (IKA) or 2) a Pine WaveNow potentiostat. Graphite electrodes were cleaned by sonication in demi-water for 5 min, then polished with a 220-sanding sponge, rinsed with acetone or demiwater, and dried in air. Platinum electrodes were sonicated in demi-water for 2 min, then rinsed with acetone and dried in air. The reference electrodes were rinsed with acetone and dried in air.

NMR spectra (^1^H and ^13^C) were obtained with either a Bruker Avance III 500 MHz spectrometer or a Varian AMX400 spectrometer with reference to residual solvent peaks: CDCl_3_ peaks at 7.26 (^1^H) and 77.16 ppm (^13^C); CD_3_OD peaks at 3.31 ppm (^1^H) and 49.00 ppm (^13^C); and D_2_O peaks at 4.79 (^1^H). All data are reported as follows: Chemical shifts (δ), multiplicity (s = singlet, d = doublet, dd = doublet of doublets, t = triplet, q = quartet, m = multiplet) coupling constant *J* (Hz), and integration. Voltammetric experiments were performed using a Pine WaveNow potentiostat. Chromatography was performed using an automated Büchi Reveleris X2.

### Cyclic Voltammetry

All cyclic voltammetry (CV) experiments were carried out using a Pine WaveNow potentiostat. They were carried out with a glassy carbon (GC) working electrode (3 mm diameter) and a platinum wire counter electrode (1.0 cm, spiral wire). The working potentials were measured against a Ag/AgNO_3_ reference electrode (internal solution, 0.1 M Bu_4_NPF_6_ and 0.01 M AgNO_3_ in MeCN). The GC working electrode was polished with alumina powder (5 μm) and water before each experiment. The redox potential values were adjusted relative to Fc^+^/Fc and electrochemical studies in organic solvent mixtures were recorded accordingly.

### Constant Current Electrolysis

CCE experiments were carried out using ether a IKA Electrasyn 2.0 in an undivided cell equipped with an IKA graphite anode (Plate, 5.25 cm × 0.8 cm × 0.2 cm, ∼3 cm was immersed in the solution) and a platinum plate cathode (Plate, 5.25 cm × 0.8 cm × 0.1 mm, ∼3 cm was immersed in the solution) or a graphite cathode (Plate, 5.25 cm × 0.8 cm × 0.2 cm, ∼3 cm was immersed in the solution) if stated, or using a Pine WaveNow potentiostat with an undivided cell equipped with a graphite anode (Rod, 6.0 cm × o.d. 0.6 cm, ∼3 cm was immersed in the solution), a platinum wire cathode (1.0 cm, spiral wire), and a Ag/AgNO_3_ reference electrode.

In a 5 mL vial, the starting material (0.25 mmol, 1 equiv), ammonium tetrafluoroborate (52 mg, 0.5 mmol, 2 equiv), and 0.5 mL of freshly prepared stock solution of 0.15 M TEMPO in MeCN were added in 5 mL of a 9:1 MeCN:pyridine mixture. The mixture was stirred for 2 min before the vial was capped and equipped with the electrodes. Constant current electrolysis (10 mA, *j* = 1.6 mA/cm^2^) was conducted at room temperature, with stirring at 1000 rpm and 6 F/mol of charge was passed. At the end of the reaction, 0.5 mL of a 0.5 M solution of 1,3,5-trimethoxybenzene as an internal standard in MeCN was added to the reaction mixture, and an aliquot of 75 μL was taken for qNMR analysis in CD_3_OD.

### Isolation

Method 1: At the end of the reaction, the crude reaction mixture was transferred to a round-bottomed flask and concentrated in vacuo. The remaining crude was dissolved in 2 mL of pyridine, to which acetic anhydride (350 μL, 3.75 mmol, 15 equiv) was added. The reaction mixture was stirred at room temperature for 16 h. The mixture was diluted with EtOAc and washed with 1 M HCl_aq_, dried over MgSO_4_ and evaporated under reduced pressure. The product was isolated upon column chromatography on silica gel (20% to 50% EtOAc in heptane).

Method 2: At the end of the reaction, the crude reaction mixture was transferred to a round-bottom flask and concentrated in vacuo. The remaining crude was dissolved in 2 mL of pyridine, to which acetic anhydride (350 μL, 3.75 mmol, 15 equiv) was added. The reaction mixture was stirred at room temperature for 16 h. Upon full conversion, the mixture was concentrated under a reduced pressure. The concentrated reaction mixture was diluted with EtOAc and washed once with aqueous copper sulfate solution and once with brine, dried over NaSO_4_ and evaporated under reduced pressure. The product was isolated upon column chromatography on silica gel (20% to 50% EtOAc in heptane).

### Large Scale

A scale up electrolysis was performed in bath mode in a multi-electrode batch electrolysis reactor. The reactor utilized a precedented design^1^ that constituted a 100 mL glass Mettler-Toledo reaction vessel, a PFTE electrode holder, and a custom annular circuit board. Seven working electrodes (graphite rod) and seven counter electrodes (316 stainless steel) with diameters of 0.64 cm were distributed evenly within the reaction vessel. To this reactor were added methyl-α-d-glucopyranoside **1** (1.5 g, 8 mmol, 1 equiv, 100 mM), ammonium tetrafluoroborate (1.6 g, 16 mmol, 2 equiv, 200 mM) and TEMPO (251 mg, 1.6 mmol, 30 mol %) in 80 mL of a 9:1 MeCN:pyridine solvent mixture. A constant current electrolysis (100 mA, *j* = 2.1 mA/cm^2^) was applied at room temperature, with stirring at 1000 rpm until 8 F/mol of charge. At the end of the reaction, an aliquot of 75 μL was taken for qNMR analysis in CD_3_OD with 1,3,5-trimethoxybenzene as an internal standard.

### Time Course Experiment

Reactions were carried out in an undivided cell equipped with a graphite anode (Rod, 6.0 cm × o.d. 0.6 cm, ∼3 cm was immersed in the solution), a platinum wire cathode (1.0 cm, spiral wire), and a Ag/AgNO_3_ reference electrode. A mixture of methyl-α-d-glucopyranoside **1** (48 mg, 0.25 mmol, 1 equiv), ammonium tetrafluoroborate (52 mg, 0.5 mmol, 2 equiv) and TEMPO (12 mg, 0.075 mmol, 30 mol %) were added in 5 mL of 9:1 MeCN:pyridine. Constant current electrolysis (4 mA, *j* = 0.7 mA/cm^2^) was conducted for 840 min (charged passed = 8 F/mol) at room temperature, with a stirring speed of 1000 rpm. During electrolysis, an aliquot of the reaction mixture (50 μL) was drawn after addition of each Faraday. To this was added 25 μL of a solution of 0.1 M 1,3,5-trimethoxybenzene in MeCN as an internal standard, diluted with CD_3_OD and submitted to ^1^H NMR analysis.

## Supplementary Material



## Notes

General procedure for electrochemical ammoxidation; general procedure for the isolation of the prepared nitriles; reaction optimization; cyclic voltammetry (CV) studies; time course experiment for the ammoxidation of methyl-α-d-glucopyranoside; potential traces; characterization data of the electrochemical oxidations; NMR and HRMS spectra (PDF)
