# Supplementary material for: Electrochemical Ammoxidation of Unprotected Glycosides
Source: ACS Electrochem. 2025 May 15;1(8):1515–22. doi: 10.1021/acselectrochem.5c00111 (PMC12337100; doi:10.1021/acselectrochem.5c00111)
Supplement: Supplementary file 1 [file ec5c00111_si_001.pdf]

Supporting Information

Electrochemical ammoxidation of unprotected glycosides

Imke M. A. Bartels,[a] Md Asmaul Hoque,[b] J. Prathap Kaniraj,*[,a] Mathew R. Johnson,[b] Sebastian B. Beil,[a,c] Shannon S. Stahl,*[,b] Martin D. Witte,*[,a] Adriaan J. Minnaard*[,a]

[a] Stratingh Institute for Chemistry, University of Groningen, Nijenborgh 7, 9747 AG

Groningen, The Netherlands. E-mail: *J.P.Kaniraj@rug.nl* , *M.D.Witte@rug.nl* , *A.J.Minnaard@rug.nl*

[b] Department of Chemistry, University of Wisconsin-Madison, Madison, Wisconsin 53706, United States. E-mail: stahl@chem.wisc.edu

[c] Max-Planck-Institute for Chemical Energy Conversion, Stiftstraße 34–36, 45470 Mülheim an der Ruhr, Germany

Table of contents

1. General Information S2

2. General procedure for electrochemical ammoxidation S2

3. General procedure for the large scale electrochemical ammoxidation S3

4. General procedure for the isolation of the prepared nitriles S4

4.1 method 1: S4

4.2 method 2: S4

5. Reaction optimization S4

5.1 Initial studies on the electrochemical ammoxidation on protected glucoside 1 using IKA S4

5.2 Initial solvent screening S4

5.3 Initial reaction optimization in the IKA electrasyn set-up S5

5.4. Final optimization in the Pine WaveNow potentiostat set-up S8

6. Cyclic voltammetry (CV) studies S9

6.1 Cyclic voltammetry studies in the solvent mixture *t*-BuOH : MeCN : water in 8 : 1 : 1 ratio S10

6.2 Cyclic voltammetry studies in MeCN : pyridine 9 : 1 .... S14

7. Time course experiment for the ammoxidation of methyl-α-D-glucopyranoside S18

8. Potential traces S20

9. Characterization data of the electrochemical oxidations S20

10. NMR and HRMS spectra S26

10.1 qNMR spectra compared to isolated product and crude NMR spectra S26

10.2 Isolated ^{1}H and ^{13}C-NMR spectra S36

10.3 HRMS spectra S41

References S44

1. General Information

All solvents were purchased from commercially available sources and used without further purification. All ammonium salts, carbohydrates and mediators were purchased form Sigma-Aldrich, TCI, Biosynth and Combi-Blocks. All chemicals were used without further purification. Electrochemical reactions were carried out either using 1) an ElectraSyn 2.0 device (IKA) or 2) a Pine WaveNow potentiostat. Graphite electrodes were cleaned by sonication in demi-water for 5 min, then polished with a 220-sanding sponge and rinsed with acetone or demi-water and dried in air. Platinum electrodes were sonicated in demi-water for 2 min, then rinsed with acetone and dried in air. The reference electrodes were only rinsed with acetone and dried in air.

NMR spectra (^1H and ^{13}C) were obtained with either a Bruker Avance III 500 MHz spectrometer or a Varian AMX400 spectrometer with reference to residual solvent peaks: $CDCl_3$ peaks at 7.26 ppm (^1H) and 77.16 ppm (^{13}C); CD_3OD peaks at 3.31 ppm (^1H) and 49.00 (^{13}C); D_2O peaks at 4.79 (^1H). All data is reported as follows: Chemical shifts (δ), multiplicity (s = singlet, d = doublet, dd = doublet of doublets, t = triplet, q = quartet, m = multiplet) coupling constant J (Hz), and integration. Voltametric experiments were performed using a Pine WaveNow potentiostat. Chromatography was performed using an automated Büchi Reveleris® X2.

2. General procedure for electrochemical ammoxidation

1) In an IKA electrasyn

Reactions were carried out using a IKA Electrasyn 2.0 in an undivided cell equipped with an IKA graphite anode (Plate, 5.25 cm x 0.8 cm x 0.2 cm, ~ 3 cm was immersed in the solution) and a platinum plate cathode (Plate, 5.25 cm x 0.8 cm x 0.1 mm, ~ 3 cm was immersed in the solution) or a graphite cathode (Plate, 5.25 cm x 0.8 cm x 0.2 cm, ~ 3 cm was immersed in the solution) if stated. (see **Figure S1** for images of the cell assembly)

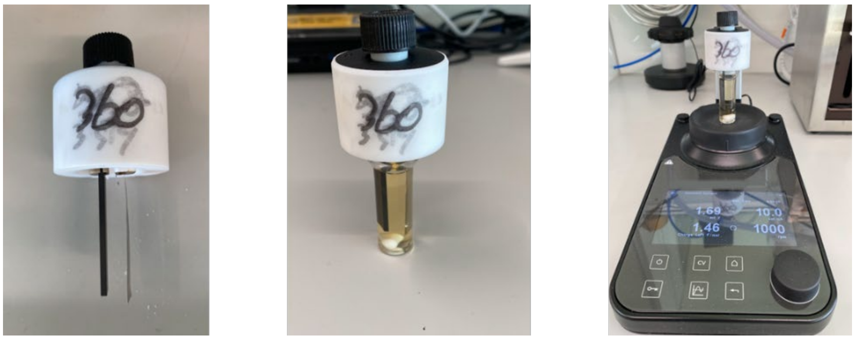

Figure S1: Pictures of the IKA electrasyn 2.0 undivided cell set-up

2) In a Pine WaveNow potentiostat set-up

Reactions were carried out using an undivided cell equipped with a graphite anode (Rod, 6.0 cm x Ø0.6 cm , ~ 3 cm was immersed in the solution), a platinum wire cathode (1.0 cm, spiral wire) and a $Ag/AgNO_3$ reference electrode. (see **Figure S2** for images of the cell assembly)

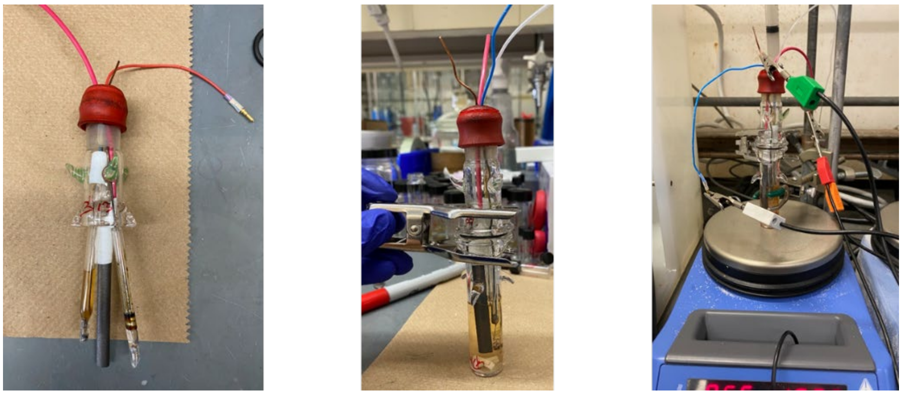

Figure S2: Pictures of the electrochemical undivided cell set-up

In a 5 mL vial, the starting material (0.25 mmol, 1 equiv), ammonium tetrafluoroborate (52 mg, 0.5 mmol, 2 equiv) and 0.5 mL of freshly prepared stock solution of 0.15 M TEMPO in MeCN were added in 5 mL of a 9 : 1 MeCN : pyridine. The mixture was stirred for 2 min, before the vial was capped and equipped with the electrodes. Constant current electrolysis (10 mA, j = 1.6 mA/cm^2) was conducted at room temperature, with stirring at 1000 rpm and 6 F/mol of charge was passed. At the end of the reaction, 0.5 mL of a 0.5 M solution of 1, 3, 5-trimethoxy benzene as an internal standard in MeCN was added to the reaction mixture and an aliquot of 75 µL was taken for qNMR analysis in CD_3OD.

3. General procedure for the large scale electrochemical ammoxidation

A scale up electrolysis was performed in bath mode in a multi-electrode batch electrolysis reactor. The reactor utilized a precedented design[1] that constituted a 100 mL glass Mettler-Toledo reaction vessel, a PFTE electrode holder, and a custom annular circuit board. Seven working electrodes (graphite rod) and seven counter electrodes (316 stainless steel) with a diameter of 0.64 cm were distributed evenly within the reaction vessel. To this reactor, methyl-α-D-glucopyranoside **1** (1.5 g, 8 mmol, 1 equiv., 100 mM), ammonium tetrafluoroborate (1.6 g, 16 mmol, 2 equiv, 200 mM) and TEMPO (251 mg, 1.6 mmol, 30 mol%) were added in 80 mL 9 : 1 MeCN : pyridine solvent mixture. A constant current electrolysis (100 mA, j = 2.1 mA/cm^2) was applied at room temperature, with stirring at 1000 rpm until the passage of 8 F/mol of charge. At the end of the reaction, an aliquot of 75 µL was taken for qNMR analysis in CD_3OD with 1, 3, 5-trimethoxy benzene as an internal standard.

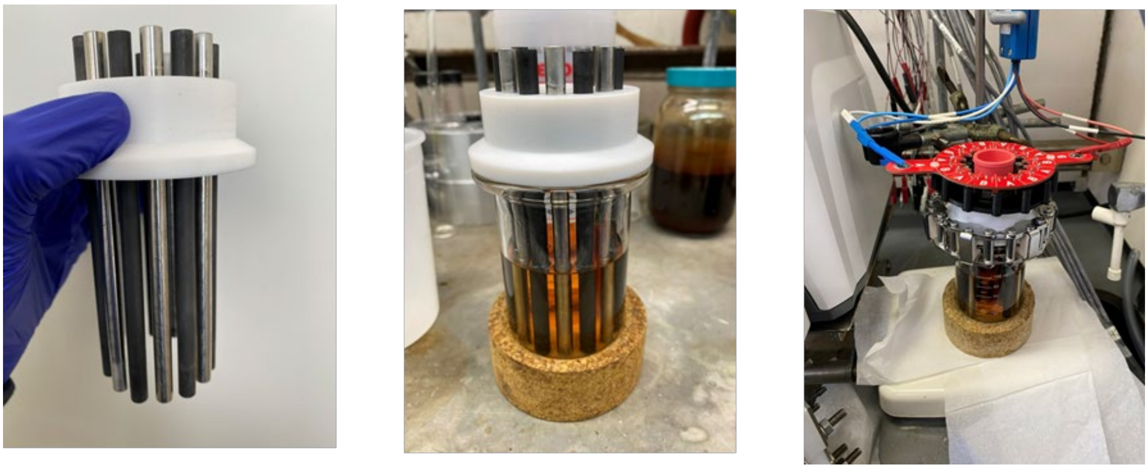

Figure S3: Gram scale electrochemical reaction in a multi-electrode batch electrolysis reactor.

4. General procedure for the isolation of the prepared nitriles

4.1 method 1:

At the end of the reaction, the crude reaction mixture was transferred to a round-bottom flask and concentrated *in vacuo*. The remaining crude was dissolved in 2 mL pyridine, to which acetic anhydride (350 µL, 3.75 mmol, 15 equiv) was added. The reaction mixture was stirred at room temperature for 16 h. The mixture was diluted with EtOAc and washed with 1 M HCl_{aq}, dried over $MgSO_4$ and evaporated under reduced pressure. The product was isolated upon column chromatography on silica gel (20% to 50% EtOAc in heptane).

4.2 method 2:

At the end of the reaction, the crude reaction mixture was transferred to a round-bottom flask and concentrated *in vacuo*. The remaining crude was dissolved in 2 mL pyridine, to which acetic anhydride (350 µL, 3.75 mmol, 15 equiv) was added. The reaction mixture was stirred at room temperature for 16 h. Upon full conversion, the mixture was concentrated under reduced pressure. The concentrated reaction mixture was diluted with EtOAc and washed once with aqueous copper sulfate solution and once with brine, dried over $NaSO_4$ and evaporated under reduced pressure. The product was isolated upon column chromatography on silica gel (20% to 50% EtOAc in heptane).

5. Reaction optimization

5.1 Initial studies on the electrochemical ammoxidation of protected glucoside 1 using IKA

Among the various supporting electrolytes screened ($NaClO_4$, $LiClO_4$, Me_4NBF_4, and Bu_4NOAc) in MeCN, it was found that addition of those supporting electrolytes did not improve the conductivity of the solution that prevent us from running the electrochemical reaction. Utilizing the ammonium acetate as both the nitrogen source and electrolyte improve the conductivity upon using water as a co-solvent to aid solubility. Using constant current electrolysis allowed for full consumption of the starting material **1**[2], assessed based on TLC (60% EtOAc in pentane). Crude ^{1}H-NMR showed the desired nitrile **2** being the major product (**Figure S4**).

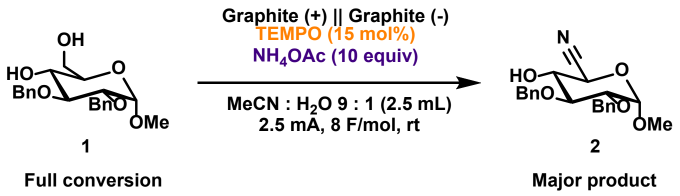

Figure S4: Electrochemical oxidation of methyl 2,3-di-*O*-benzyl-α-D-glucopyranoside **1** using the IKA Electrasyn 2.0 set-up.

5.2 Initial solvent screening

Among the various solvents screened, a mixture of *tert*-butanol, acetonitrile, and water in a 8 : 1 : 1 ratio was initially found to have the best trade-off between solubility of the substrate and ammonium acetate, conductivity and the product to side-product ratio. This solvent mixture afforded the highest reproducible conversion of substrate towards the desired product **4**.

Solvent mixtures with higher water content allow to dissolve all substrates and the reaction mixture showed good conductivity, but the selectivity towards product formation shifts from nitrile **4** to the undesired carboxylate **5** (**Table S1**, entry 1 & 10). The reaction mixture remained homogeneous in alcohol based solvent and has relatively good conductivity, however, solvent oxidation outcompetes the oxidation of the substrate during electrolysis (**Table S1**, entries 2 & 3). All other tested solvents

either do not dissolve the substrates well enough or do not have an acceptable conductivity to run the electrolysis reaction (**Table S1**, entries 4-9).

Table S1: Evaluation of different solvents[a]

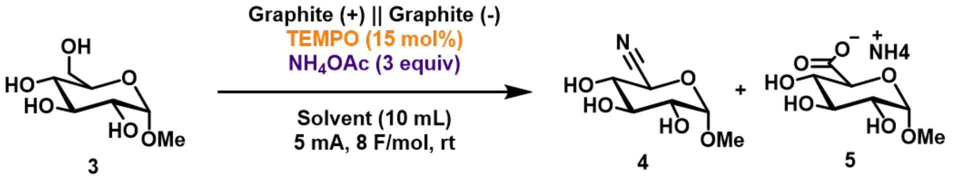

Entry	Solvent	Dissolved	Conductivity (Siemens)	Conversion **3** (%)	Ratio **4** : **5** (%)
1[b]	water	Yes	-	20	0 : 100
2	MeOH	Yes	5.8 mS	NR	-
3	*i*-PrOH	No	-	NR	-
4	acetone	No	130 µS	-	-
5	THF	No	14 µS	-	-
6	DME	No	7 µS	-	-
7	diethylcarbonate	No	6 µS	-	-
8	*t*-BuOH : water 1 : 2	No	90 µS	-	-
9	*t*-BuOH : water 9 : 1	No	50 µS	-	-
10	*t*-BuOH : MeCN : water 1 : 1 : 1	Yes	3 mS	71	36 : 64
11[c]	*t*-BuOH : MeCN : water 4.5 : 4.5 : 1	Yes	800 µS	NR	NR
**12**	***t*-BuOH : MeCN : water 8 : 1 : 1**	**Yes**	**280 µS**	**59**	**76 : 24**

[a]Reaction conditions: Using the IKA Electrasyn 2.0 set-up*,* methyl-α-D-glucopyranoside (124 mg, 0.64 mmol, 1 equiv) **3**, TEMPO (15 mg, 0.096 mmol, 15 mol%), ammonium acetate (148 mg, 1.92 mmol, 3 equiv), in 10 mL of the solvent (mixture) in an undivided cell with graphite as both the anode and cathode. Constant current electrolysis at 5 mA for 8 F/mol using the IKA electrasyn 2.0. Conversion based on ^{1}H-NMR; integration of the anomeric signal of the substrate and all formed products set to 1 to give conversion and product ratio. [b]NAc-TEMPO used instead for its better solubility in water. [c]Reaction was stopped after addition of 2 F due to too high observed potential.

5.3 Initial reaction optimization in the IKA electrasyn set-up

5.3.1 Varying TEMPO loading

Among the various loadings of TEMPO tested, it was found that 20 mol% TEMPO leads to the highest conversion of substrate with good selectivity towards the desired product **2** (**Table S2**).

Table S2: Optimization of the TEMPO loading[a]

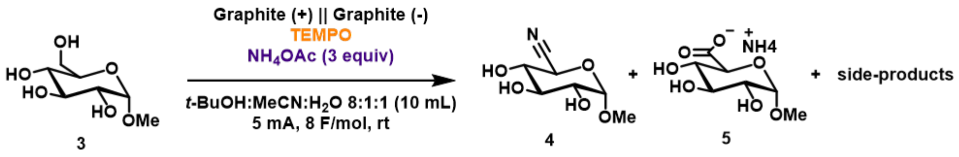

Entry	TEMPO (mol%)	Conversion **3** (%)	Ratio **4** : **5** : **side-products** (%)
1	10	69	67 : 19 : 14
2	15	64	70 : 20 : 10
**3**	**20**	**95**	**68 : 18 : 14**

[a]Reaction conditions: Using the IKA Electrasyn 2.0 set-up*,* methyl-α-D-glucopyranoside (124 mg, 0.64 mmol, 1 equiv) **3**, TEMPO, ammonium acetate (148 mg, 1.92 mmol, 3 equiv), in *t*-BuOH : MeCN : water 8 : 1 : 1 (10 mL) in an undivided cell with graphite as both the anode and cathode. Constant current electrolysis at 5 mA for 8 F/mol using the IKA electrasyn 2.0. Conversion based on ^{1}H-NMR; integration of the anomeric signal of the substrate and all formed products set to 1 to give conversion and product ratio.

5.3.2 Evaluation of different mediators

It was determined that TEMPO was the most optimal among the various screened mediators (**Table S3**, entry 1). The use of 4-NAc-TEMPO resulted in the same conversion to the desired nitrile **4**, but led to more acid **5** formation (**Table S3**, entry 2). 4-hydroxy-TEMPO led to less conversion (**Table S3**, entry 3), which we hypothesize can be atributed to the fact that 4-hydroxy-TEMPO is less stable in solution and thus decomposes faster.

Table S3: Evaluation of reaction conditions with different mediators[a]

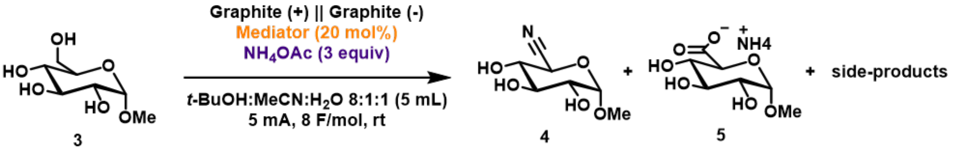

Entry	Mediator (mol%)	Conversion **3** (%)	Ratio **4** : **5** : **side-products** (%)
**1**	**TEMPO**	**94**	**70 : 16 : 14**
2	4-NAc-TEMPO	100	67: 20 : 13
3	4-Hydroxy-TEMPO	85	65: 20 : 15

[a]Reaction conditions: Using the IKA Electrasyn 2.0 set-up, methyl-α-D-glucopyranoside (62 mg, 0.32 mmol, 1 equiv) **3**, mediator (20 mol%), ammonium acetate (74 mg, 0.96 mmol, 3 equiv), in *t*-BuOH : MeCN : water 8 : 1 : 1 (5 mL) in an undivided cell with graphite as both the anode and cathode. Constant current electrolysis at 5 mA for 8 F/mol using the IKA electrasyn 2.0. Conversion based on ^{1}H-NMR; integration of the anomeric signal of the substrate and all formed products set to 1 to give conversion and product ratio.

5.3.3 Results at varying equivalents of NH_4OAc

Varying the amount of ammonium acetate at the optimized mediator conditions revealed that 3 equiv provided the highest overall conversion and reasonable chemoselectivity to the desired product **4**. Increasing the number of equiv of NH_4OAc suppressed the formation of acid **5** and the other side-products. This did come at the expense of conversion. Conversely, lowering the loading of NH_4OAc led to low substrate conversion and increase in side-products formation.

Table S4: Optimization of reaction conditions by varying the number of eq of NH_4OAc[a]

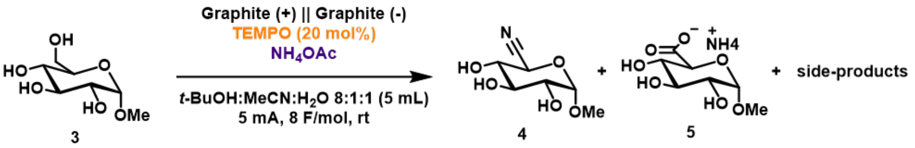

Entry	NH_4OAc (equiv)	Conversion **3** (%)	Ratio **4** : **5** : **side-products** (%)
1	1	60	52 : 32 : 16
2	2	66	59 : 27 : 14
**3**	**3**	**96**	**71 : 19 : 10**
4	5	86	79 : 11 : 10
5	10	83	77 : 15 : 8
6	20	57	81 : 11 : 8

[a]Reaction conditions: Using the IKA Electrasyn 2.0 set-up, methyl-α-D-glucopyranoside (62 mg, 0.32 mmol, 1 equiv) **3**, TEMPO (10 mg, 0.064 mmol, 20 mol%), ammonium acetate in *t*-BuOH : MeCN : water 8 : 1 : 1 (5 mL) in an undivided cell with graphite as both the anode and cathode. Constant current electrolysis at 5 mA for 8 F/mol using the IKA electrasyn 2.0. Conversion based on ^{1}H-NMR; integration of the anomeric signal of the substrate and all formed products set to 1 to give conversion and product ratio.

5.3.4 Evaluation of different ammonia sources

Various ammonia sources were tested, ammonium acetate showed better reactivity and chemoselectivity. Both ammonium chloride and ammonium hexafluorophosphate (**Table S5**, entries 2&3), provided excellent conductivity for the reaction to run electrolysis. However, with a pH of 4.9 and 5.5 respectively, the reaction solutions were too acidic for the ammoxidation to occur. Ammonium carbamate (**Table S5**, entry 3) did provide sufficient conductivity to run the electrolysis, but suffered from poor conversion and chemoselectivity. All other tested ammonia sources did not sufficiently dissolve well to provide sufficient conductivity to run the electrolysis (**Table S5**, entries 4-7).

Table S5*:* Optimization of reaction conditions with different ammonia sources[a]

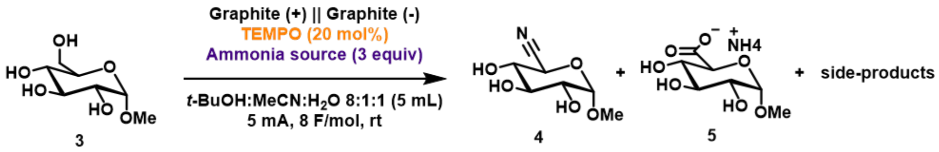

Entry	Ammonia source	Conversion **3** (%)	Ratio **4** : **5** : **side-products** (%)
**1**	**NH_4OAc**	**96**	**71 : 19 : 10**
2	NH_4PF_6	0	0 : 0 : 0
3	NH_4Cl	0	0 : 0 : 0
4	$H_2NCO_2NH_4$	25	52 : 0 : 48
5	NH3 (aq.)	-	-
6	NH_4HCO_3	-	-
7	$(NH_4)_2SO_4$	-	-
8	$(NH_4)_2HPO_4$	-	-

[a]Reaction conditions: Using the IKA Electrasyn 2.0 set-up, methyl-α-D-glucopyranoside (62 mg, 0.32 mmol, 1 equiv) **3,** TEMPO (10 mg, 0.064 mmol, 20 mol%), ammonia source (3 equiv) in *t*-BuOH : MeCN : water 8 : 1 : 1 (5 mL) in an undivided cell with graphite as both the anode and cathode. Constant current electrolysis at 5 mA for 8 F/mol using the IKA electrasyn 2.0. Conversion based on ^{1}H-NMR; integration of the anomeric signal of the substrate and all formed products set to 1 to give conversion and product ratio.

5.3.5 Optimization of the constant current during electrolysis

Two electrolysis conditions different from the established conditions were studied, but neither of these was found to improve the conversion or chemoselectivity (**Table S6**).

Table S6: Optimization of reaction conditions with different constant current[a]

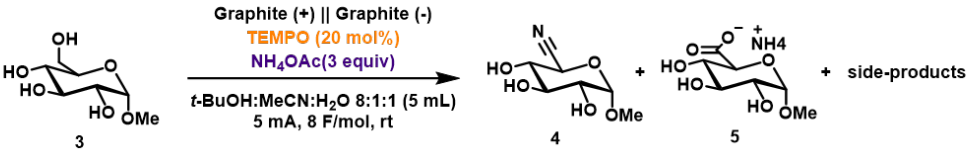

Entry	Deviation from standard	Conversion **3** (%)	Ratio **4** : **5** : **side-products** (%)
1	10 mA	59	69 : 22 : 9
2	alternating polarity (5 sec.)	27	52 : 33 : 15

[a]Reaction conditions: Using the IKA Electrasyn 2.0 set-up, methyl-α-D-glucopyranoside (62 mg, 0.32 mmol, 1 equiv) **3**, TEMPO (10 mg, 0.064 mmol, 20 mol%), ammonium acetate (3 equiv) in *t*-BuOH : MeCN : water 8 : 1 : 1 (5 mL) in an undivided cell with graphite as both the anode and cathode. Constant current electrolysis at 5 mA for 8 F/mol using the IKA electrasyn 2.0. Conversion based on ^{1}H-NMR; integration of the anomeric signal of the substrate and all formed products set to 1 to give conversion and product ratio.

5.4. Final optimization in the Pine WaveNow potentiostat set-up

5.4.1 Screening ammonia salts

Optimization of the ammonia salt was performed. See CV studies in section 6 and the main text for results.

5.4.2 Solvent screening

Among the various solvents screened, acetonitrile with 10% pyridine was found to be optimal (**Table S7**).

The optimal solvent had the following requirements: 1) it should not contain water, to prevent side-product formation. 2) it should (partially) dissolve the carbohydrate substrate. 3) it should provide the basic environment needed for the reaction, 4) it should be electrochemically inert. Of the limited number of solvents that meet all these requirements, it was determined that 9 : 1 acetonitrile : pyridine was the most optimal, as it gave the highest conversion of substrate and highest yield of the desired product **4** (**Table S7**, entry 4).

Table S7: Further evaluation of solvents[a]

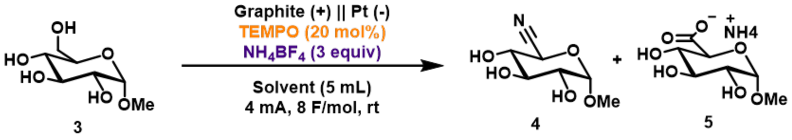

Entry	Solvent	Conversion (%)	Yield **4** (%)	Yield **5** (%)
1	*t*-BuOH : MeCN : water 8 : 1 : 1	57	8	6
2	*t*-BuOH : MeCN : water 1 : 8 : 1	83	8	12
3	DCM : water 9 : 1	77	-	-
**4**	**MeCN : pyridine 9 : 1**	**96**	**28**	**-**
5	MeCN : pyridine 8 : 2	80	24	-
6	MeCN : pyridine 7 : 3	29	26	-
7	MeCN : pyridine 6 : 4	53	27	-
8	MeCN : pyridine 1 : 1	66	12	-
9	MeCN : pyridine 1 : 9	30	23	-
10	MeCN + 1 equiv pyridine	83	12	-
11	MeCN + 3 equiv pyridine	79	12	-
12	MeCN + 5 equiv pyridine	64	26	-
13	MeCN + 10 equiv pyridine	35	7	-

[a]Reaction conditions: Using the Pine WaveNow set-up, methyl-α-D-glucopyranoside (48 mg, 0.25 mmol, 1 equiv) **3**, TEMPO (8 mg, 0.05 mmol, 20 mol%), ammonium tetrafluoroborate (79 mg, 0.75 mmol, 3 equiv) in a solvent mixture (5 mL) in an undivided cell with graphite as the anode, Pt as the cathode and $Ag/AgNO_3$ as reference electrode. Constant current electrolysis at 4 mA for 8 F/mol using the Pine WaveNow potentiostat. qNMR yields based on ^{1}H-NMR with either dimethyl sulfone or 1,3,5-trimethoxy benzene as an internal standard.

5.4.3 Evaluation of different electrodes

Four different combinations of electrodes were studied but did not provide a significant difference in conversion and yield of the reaction (**Table S8**). Therefore, because of the ease of handling, graphite as working electrode and platinum as counter electrode were considered most optimal (**Table S8**, entry 1)

Table S8: Evaluation of different electrode combinations[a]

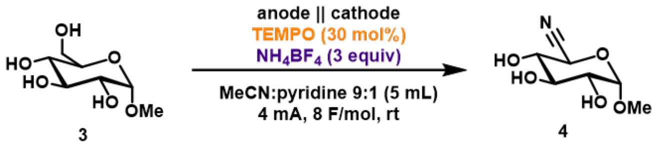

Entry	Electodes	Conversion (%)	Yield **4** (%)
**1**	**Graphite | | Pt**	**100**	**80**
2	Graphite | | Stainless steel	100	77
3	RVC | | Pt	100	83
4	RVC | | Stainless steel	100	78

[a]Reaction conditions: Using the Pine WaveNow set-up, methyl-α-D-glucopyranoside (48 mg, 0.25 mmol, 1 equiv) **3**, TEMPO (12 mg, 0.075 mmol, 30 mol%), ammonium tetrafluoroborate (79 mg, 0.75 mmol, 3 equiv) in MeCN : pyridine 9 : 1 (5 mL) in an undivided cell with an anode and a cathode and Ag/$AgNO_3$ as reference electrode. Constant current electrolysis at 4 mA for 8 F/mol using the Pine WaveNow potentiostat. qNMR yields based on ^{1}H-NMR with 1,3,5-trimethoxy benzene as an internal standard.

5.4.4 The influence of temperature on the electrolysis

A few different temperatures were studied to assess their influence on the reaction yield, but room temperature was optimal (**Table S9**).

Table S9: Evaluation of different temperatures[a]

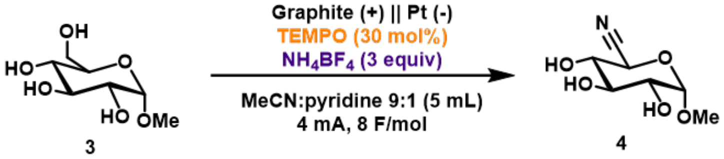

Entry	Temperature	Conversion (%)	Yield **4** (%)
**1**	**rt**	**100**	**80**
2	40 °C	90	69
3	60 °C	51	16

[a]Reaction conditions: Using the Pine WaveNow set-up, methyl-α-D-glucopyranoside (48 mg, 0.25 mmol, 1 equiv) **3**, TEMPO (12 mg, 0.075 mmol, 30 mol%), ammonium tetrafluoroborate (79 mg, 0.75 mmol, 3 equiv) in MeCN : pyridine 9 : 1 (5 mL) in an undivided cell with a graphite anode and a platinum cathode and Ag/$AgNO_3$ as reference electrode. Constant current electrolysis at 4 mA for 8 F/mol using the Pine WaveNow potentiostat. qNMR yields based on ^{1}H-NMR with 1,3,5-trimethoxy benzene as an internal standard.

6. Cyclic voltammetry (CV) studies

All cyclic voltametric (CV) experiments were carried out using a Pine WaveNow potentiostat. They were carried out with a glassy carbon (GC) working electrode (3 mm diameter) and a platinum wire counter electrode (1.0 cm, spiral wire). The working potentials were measured against a Ag/$AgNO_3$ reference electrode (internal solution, 0.1 M Bu_4NPF_6 and 0.01 M $AgNO_3$ in MeCN). The GC working electrode was polished with alumina powder (5 μ) and water before each experiment. The redox potential values were adjusted relative to Fc^+/Fc and electrochemical studies in organic solvent mixtures were recorded accordingly.

6.1 Cyclic voltammetry studies in the solvent mixture *t*-BuOH : MeCN : water in 8 : 1 : 1 ratio

6.1.1 CV study using ammonium acetate as an electrolyte and ammonia-source

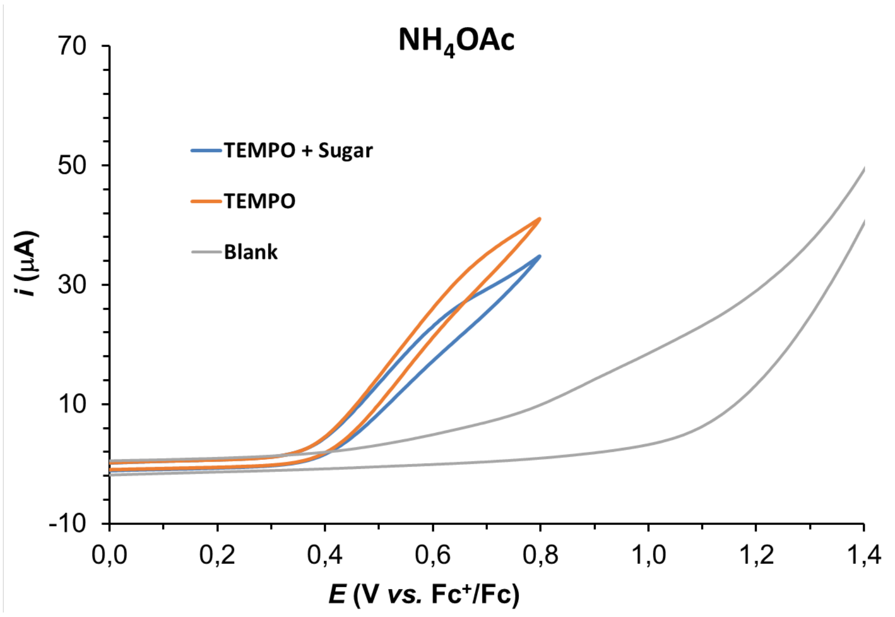

Figure S5: CV of 300 mM NH_4OAc (gray), 300 mM NH_4OAc + 2 mM TEMPO (orange), 300 mM NH_4OAc + 2 mM TEMPO + 100 mM methyl-α-D-glucopyranoside **3** (blue) in the solvent mixture containing *t*-BuOH : MeCN : water in 8 : 1 : 1 ratio. Using a glassy carbon working electrode, a platinum wire counter electrode and a Ag/$AgNO_3$ reference electrode and converted to Fc^+/Fc with a scan rate of 100 mV/s.

6.1.2 CV study using ammonium tetrafluoroborate as an electrolyte and ammonia-source

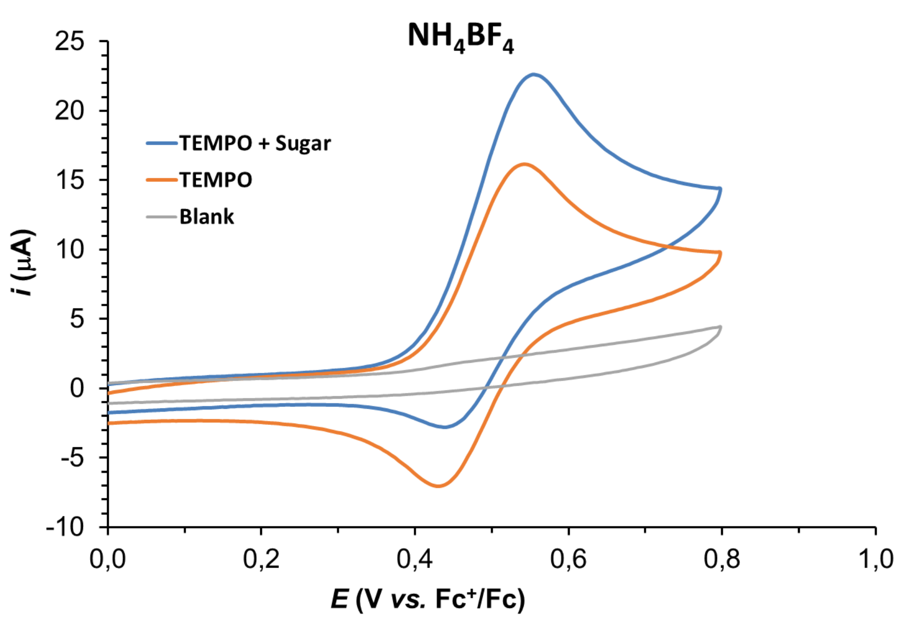

Figure S6: CV of 300 mM NH_4BF_4 (gray), 300 mM NH_4BF_4 + 2 mM TEMPO (orange), 300 mM NH_4BF_4 + 2 mM TEMPO + 100 mM methyl-α-D-glucopyranoside **3** (blue) in the solvent mixture containing *t*-BuOH : MeCN : water in 8 : 1 : 1 ratio. Using a glassy carbon working electrode, a platinum wire counter electrode and a Ag/$AgNO_3$ reference electrode and converted to Fc^+/Fc with scan rate of 100 mV/s.

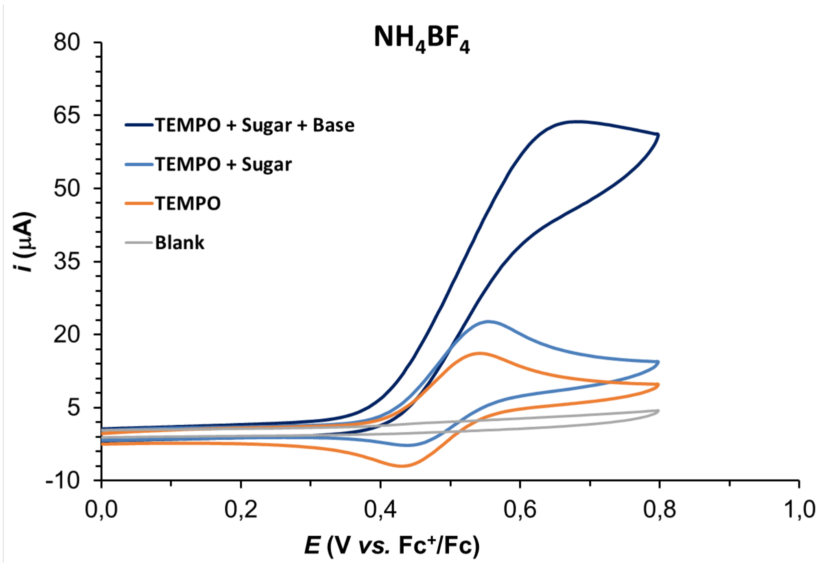

Figure S7: Cyclic voltammogram Figure S5 with one extra measurement of: 300 mM NH_4BF_4 + 2 mM TEMPO + 100 mM methyl-α-D-glucopyranoside **3** + 100 mM NH_4OH (dark blue) in the solvent mixture containing *t*-BuOH : MeCN : water in 8 : 1 : 1 ratio. Using a glassy carbon working electrode, a platinum wire counter electrode and a Ag/$AgNO_3$ reference electrode and converted to Fc^+/Fc with scan rate of 100 mV/s.

6.1.3 CV study using ammonium hexafluorophosphate as an electrolyte and ammonia-source

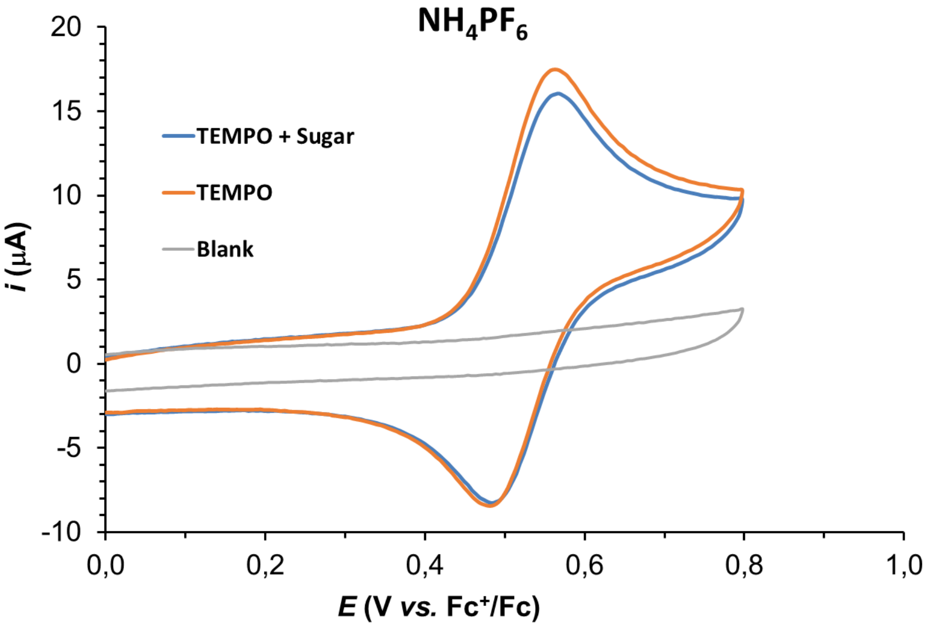

Figure S8: CV of 300 mM NH_4PF_6 (gray), 300 mM NH_4PF_6 + 2 mM TEMPO (orange), 300 mM NH_4PF_6 + 2 mM TEMPO + 100 mM methyl-α-D-glucopyranoside **3** (blue) in the solvent mixture containing *t*-BuOH : MeCN : water in 8 : 1 : 1 ratio. Using a glassy carbon working electrode, a platinum wire counter electrode and a Ag/$AgNO_3$ reference electrode and converted to Fc^+/Fc with scan rate of 100 mV/s.

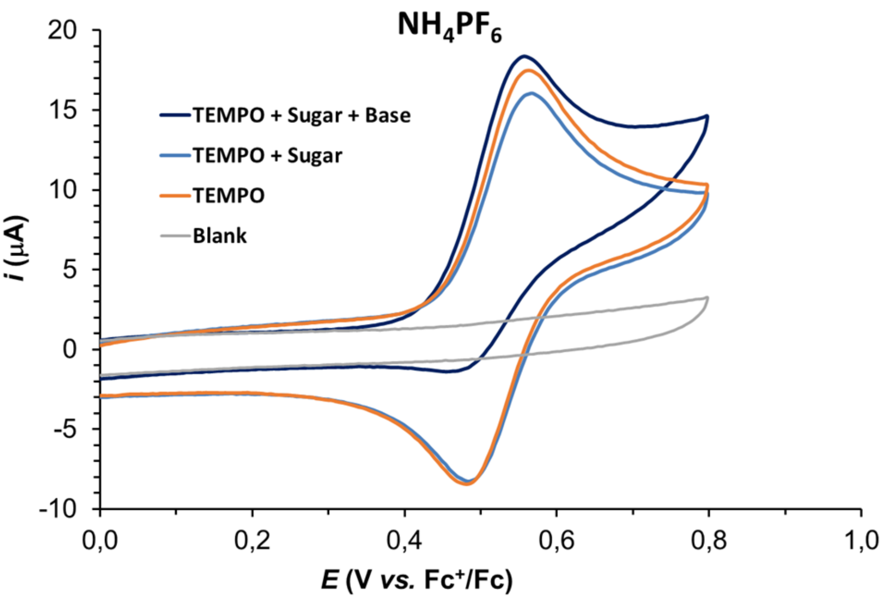

Figure S9: Cyclic voltammogram Figure S7 with one extra measurement of: 300 mM NH_4PF_6 + 2 mM TEMPO + 100 mM methyl-α-D-glucopyranoside **3** + 100 mM NH_4OH (dark blue) in the solvent mixture containing *t*-BuOH : MeCN : water in 8 : 1 : 1 ratio. Using a glassy carbon working electrode, a platinum wire counter electrode and a Ag/$AgNO_3$ reference electrode and converted to Fc^+/Fc with scan rate of 100 mV/s.

6.1.4 CV study using aqueous ammonia (ammonium hydroxide) as an electrolyte and ammonia-source

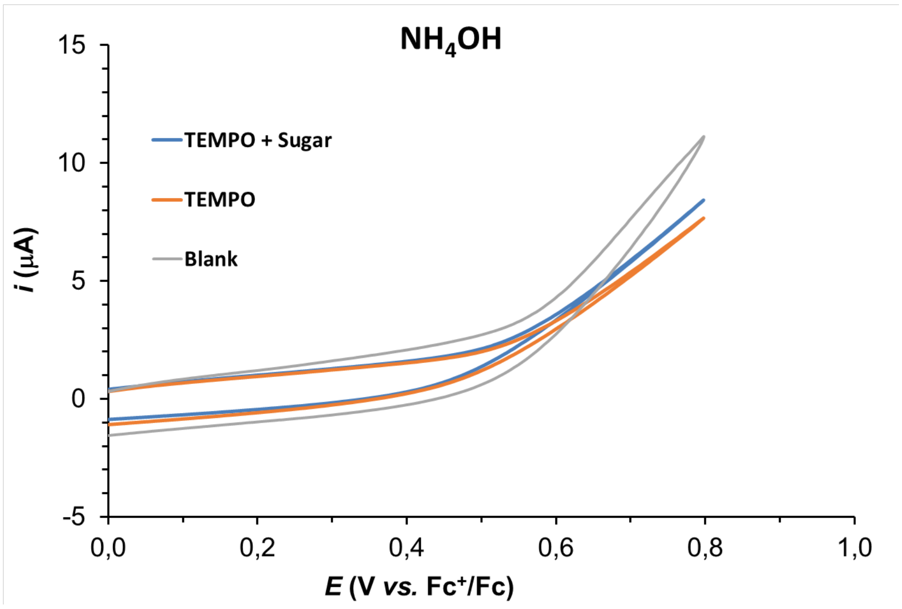

Figure S10: CV of 300 mM NH_4OH (gray), 300 mM NH_4OH + 2 mM TEMPO (orange), 300 mM NH_4OH + 2 mM TEMPO + 100 mM methyl-α-D-glucopyranoside **3** (blue) in the solvent mixture containing *t*-BuOH : MeCN : water in 8 : 1 : 1 ratio. Using a glassy carbon working electrode, a platinum wire counter electrode and a Ag/$AgNO_3$ reference electrode and converted to Fc^+/Fc with scan rate of 100 mV/s.

6.1.5 CV study using ammonium carbonate as an electrolyte and ammonia-source

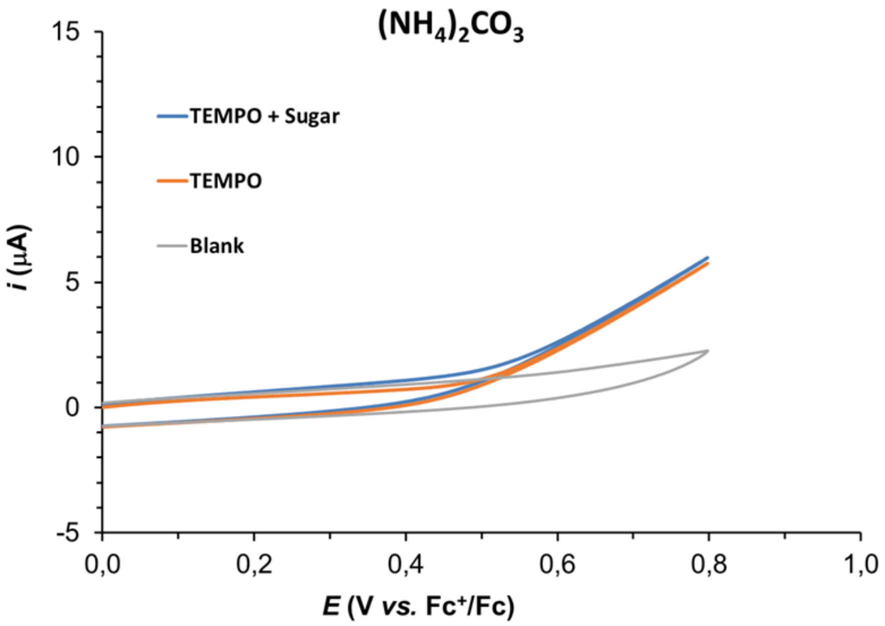

Figure S11: CV of 300 mM $(NH_4)_2CO_3$ (gray), 300 mM $(NH_4)_2CO_3$ + 2 mM TEMPO (orange), 300 mM $(NH_4)_2CO_3$ + 2 mM TEMPO + 100 mM methyl-α-D-glucopyranoside **3** (blue) in the solvent mixture containing *t*-BuOH : MeCN : water in 8 : 1 : 1 ratio. Using a glassy carbon working electrode, a platinum wire counter electrode and a $Ag/AgNO_3$ reference electrode and converted to Fc^+/Fc with scan rate of 100 mV/s.

6.1.6 CV study using ammonium formate as an electrolyte and ammonia-source

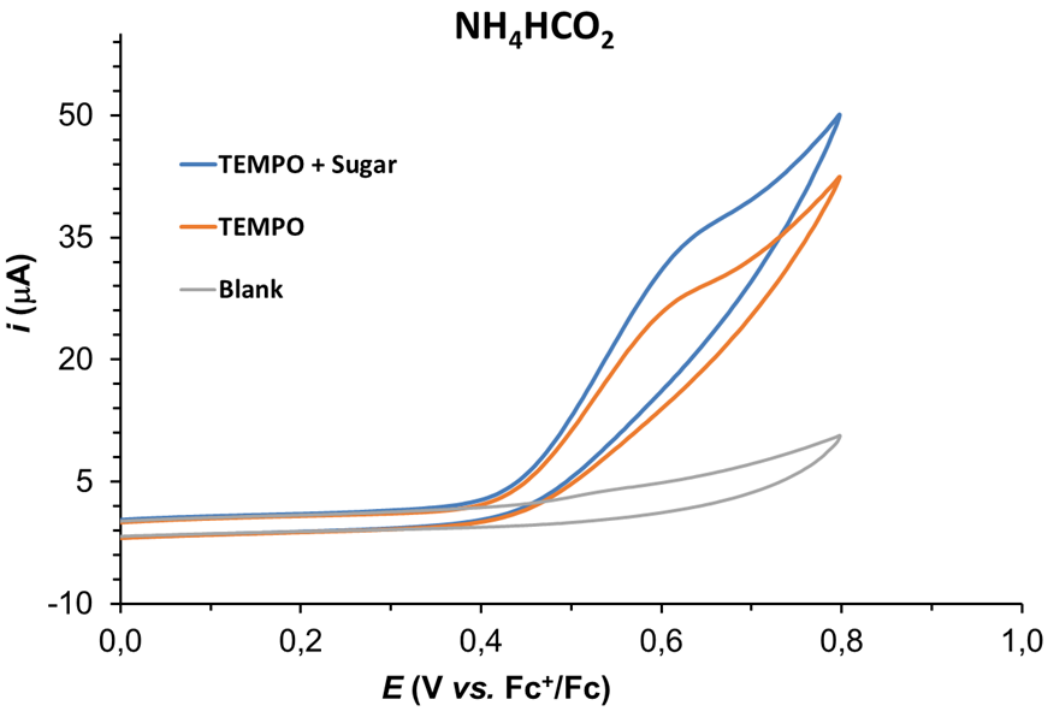

Figure S12: CV of 300 mM NH_4HCO_2 (gray), 300 mM NH_4HCO_2 + 2 mM TEMPO (orange), 300 mM NH_4HCO_2 + 2 mM TEMPO + 100 mM methyl-α-D-glucopyranoside **3** (blue) in the solvent mixture containing *t*-BuOH : MeCN : water in 8 : 1 : 1 ratio. Using a glassy carbon working electrode, a platinum wire counter electrode and a $Ag/AgNO_3$ reference electrode and converted to Fc^+/Fc with scan rate of 100 mV/s.

6.2 Cyclic voltammetry studies in MeCN : pyridine 9 : 1

6.2.1 CV Study of TEMPO in the presence of ammonium tetrafluoroborate in MeCN : pyridine 9 : 1

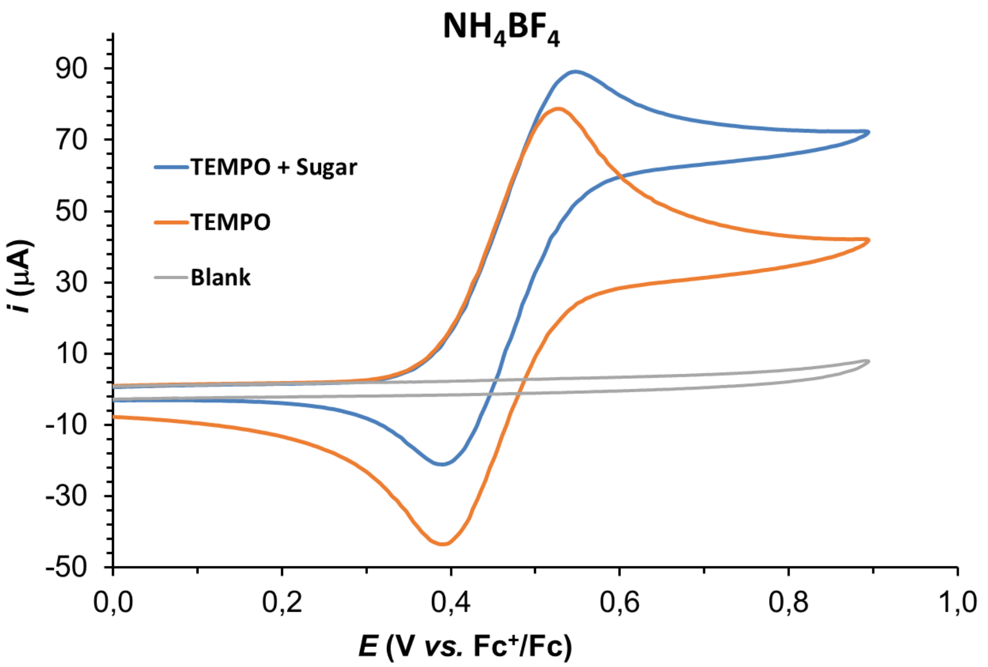

Figure S13: CV of 300 mM NH_4BF_4 (gray), 300 mM NH_4BF_4 + 2 mM TEMPO (orange), 300 mM NH_4BF_4 + 2 mM TEMPO + 100 mM methyl-α-D-glucopyranoside **3** (blue) in the solvent mixture containing MeCN : pyridine in 9 : 1 ratio. Using a glassy carbon working electrode, a platinum wire counter electrode and a Ag/$AgNO_3$ reference electrode and converted to Fc^+/Fc with scan rate of 100 mV/s.

6.2.2 CV study of TEMPO in the presence of ammonium acetate in MeCN : pyridine 9 : 1

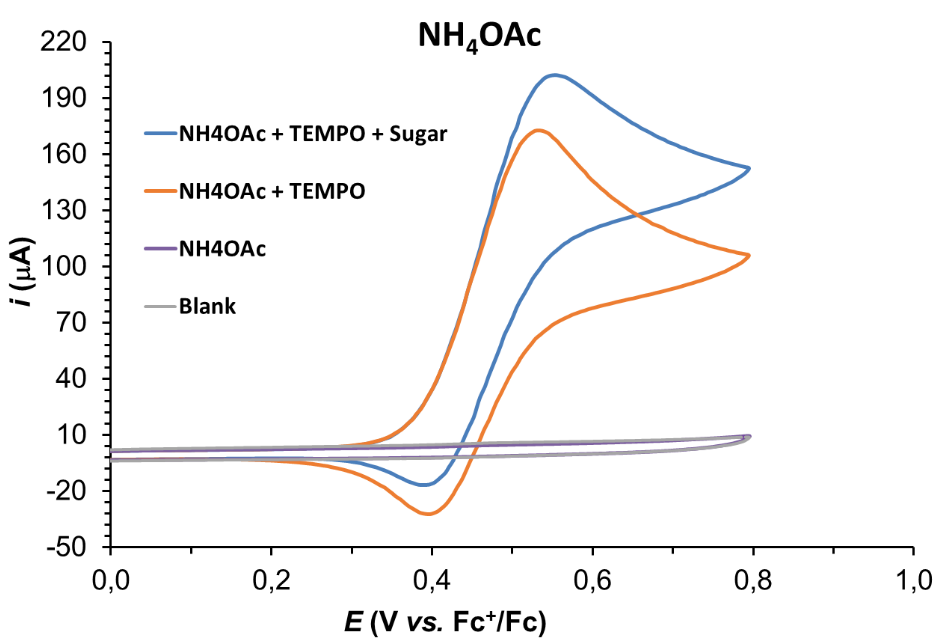

Figure S14: CV of 100 mM KPF_6 (gray), 300 mM NH_4OAc (purple), 300 mM NH_4OAc + 2 mM TEMPO (orange), 300 mM NH_4OAc + 2 mM TEMPO + 100 mM methyl-α-D-glucopyranoside **3** (blue) in the solvent mixture containing MeCN : pyridine in 9 : 1 ratio. Using a glassy carbon working electrode, a platinum wire counter electrode and a Ag/$AgNO_3$ reference electrode and converted to Fc^+/Fc with scan rate of 100 mV/s.

6.2.3 CV study of TEMPO in the presence of ammonium hexafluorophosphate in MeCN : pyridine 9 : 1

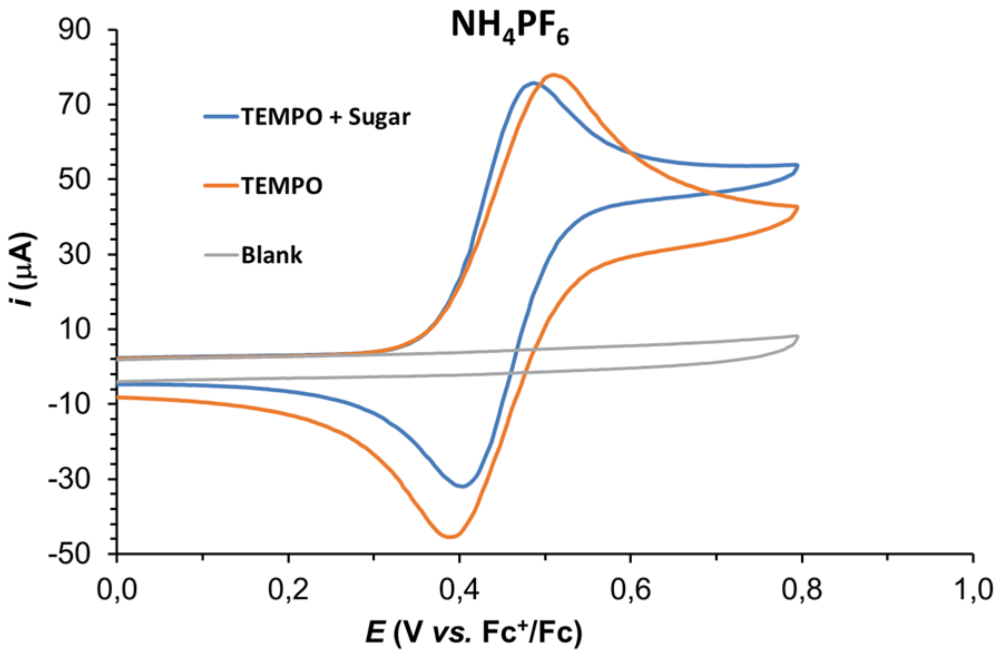

Figure S15: CV of 300 mM NH_4PF_6 (gray), 300 mM NH_4PF_6 + 2 mM TEMPO (orange), 300 mM NH_4PF_6 + 2 mM TEMPO + 100 mM methyl-α-D-glucopyranoside **3** (blue) in the solvent mixture containing MeCN : pyridine in 9 : 1 ratio. Using a glassy carbon working electrode, a platinum wire counter electrode and a Ag/$AgNO_3$ reference electrode and converted to Fc^+/Fc with scan rate of 100 mV/s.

6.2.4 CV study of TEMPO in the presence of ammonia (ammonium hydroxide) in MeCN : pyridine 9 : 1

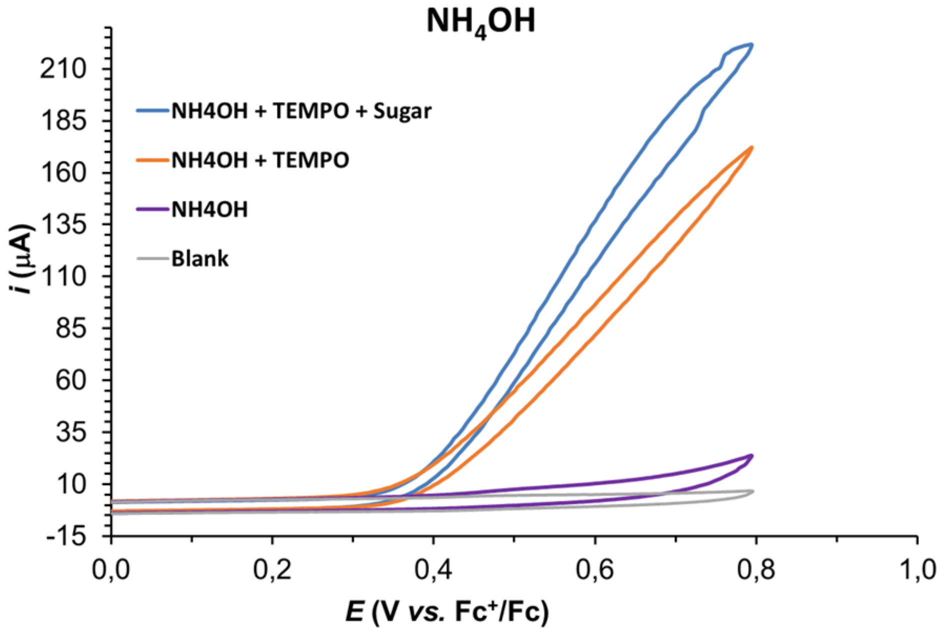

Figure S16: CV of 100 mM KPF_6 (gray), 300 mM NH_4OH (purple), 300 mM NH_4OH + 2 mM TEMPO (orange), 300 mM NH_4OH + 2 mM TEMPO + 100 mM methyl-α-D-glucopyranoside **3** (blue) in the solvent mixture containing MeCN : pyridine in 9 : 1 ratio. Using a glassy carbon working electrode, a platinum wire counter electrode and a Ag/$AgNO_3$ reference electrode and converted to Fc^+/Fc with scan rate of 100 mV/s.

6.2.5 CV study of TEMPO with methyl-β-D-glucopyranoside 3b in MeCN : pyridine 9 : 1

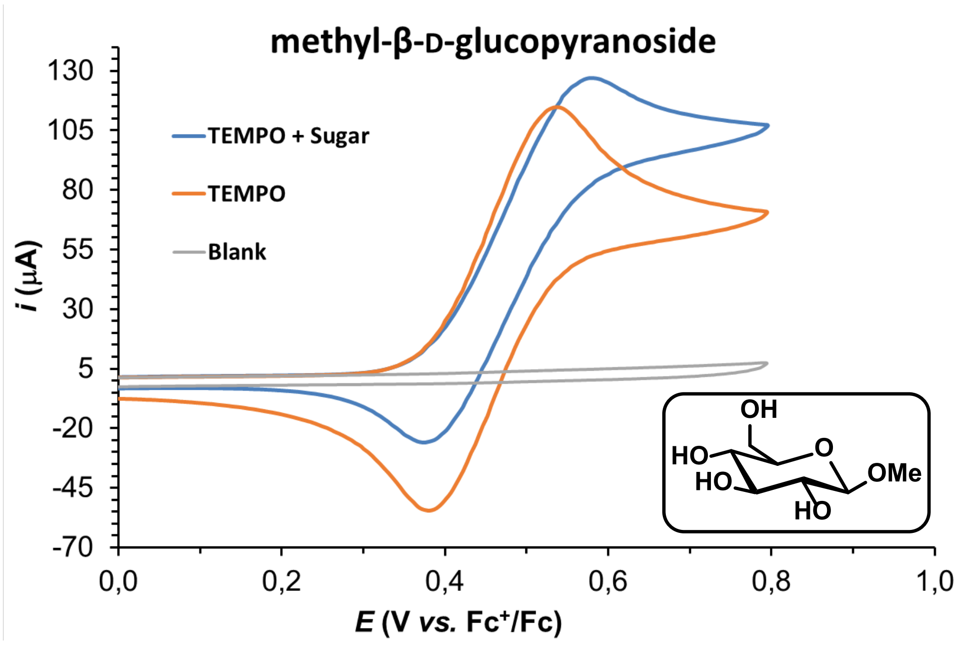

Figure S17: CV of 300 mM NH_4BF_4 (gray), 300 mM NH_4BF_4 + 2 mM TEMPO (orange), 300 mM NH_4BF_4 + 2 mM TEMPO + 100 mM methyl-β-D-glucopyranoside **3b** (blue) in the solvent mixture containing MeCN : pyridine in 9 : 1 ratio. Using a glassy carbon working electrode, a platinum wire counter electrode and a Ag/$AgNO_3$ reference electrode and converted to Fc^+/Fc with scan rate of 100 mV/s.

6.2.6 CV study of TEMPO with methyl-β-D-mannopyranoside 3c in MeCN : pyridine 9 : 1

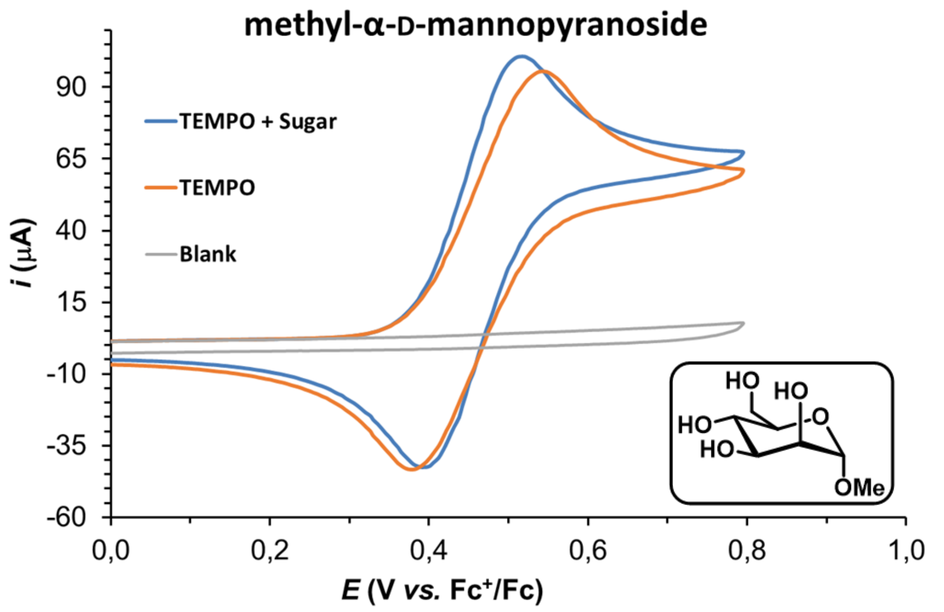

Figure 18: CV of 300 mM NH_4BF_4 (gray), 300 mM NH_4BF_4 + 2 mM TEMPO (orange), 300 mM NH_4BF_4 + 2 mM TEMPO + 100 mM methyl-β-D-glucopyranoside **3c** (blue) in the solvent mixture containing MeCN : pyridine in 9 : 1 ratio. Using a glassy carbon working electrode, a platinum wire counter electrode and a Ag/$AgNO_3$ reference electrode and converted to Fc^+/Fc with scan rate of 100 mV/s.

6.2.7 CV study of TEMPO with methyl-α-D-galactopyranoside 3d in MeCN : pyridine 9 : 1

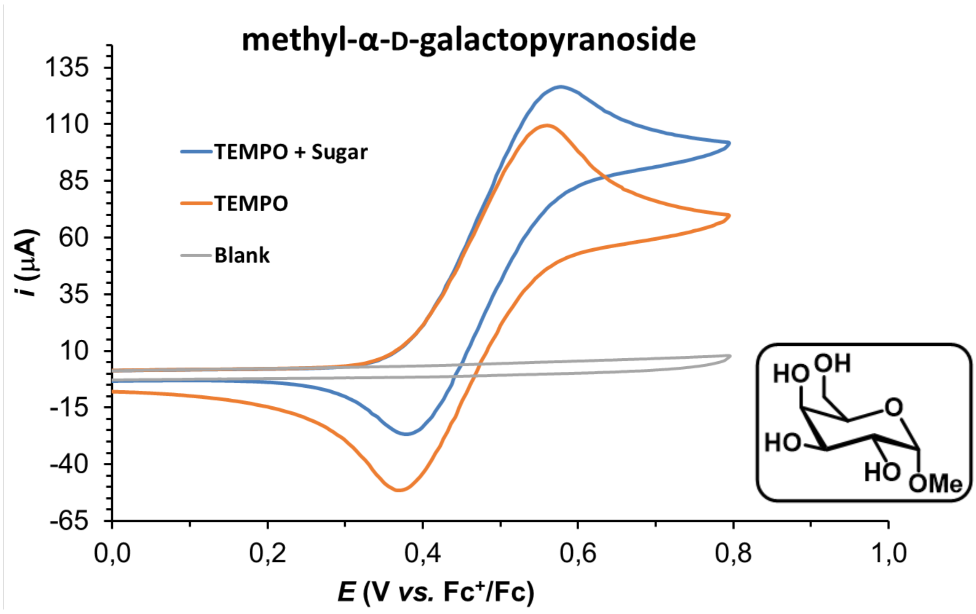

Figure S19: CV of 300 mM NH_4BF_4 (gray), 300 mM NH_4BF_4 + 2 mM TEMPO (orange), 300 mM NH_4BF_4 + 2 mM TEMPO + 100 mM methyl-α-D-galactopyranoside **3d** (blue) in the solvent mixture containing MeCN : pyridine in 9 : 1 ratio. Using a glassy carbon working electrode, a platinum wire counter electrode and a $Ag/AgNO_3$ reference electrode and converted to Fc^+/Fc with scan rate of 100 mV/s.

6.2.8 CV study of TEMPO with methyl-β-D-galactopyranoside 3e in MeCN : pyridine 9 : 1

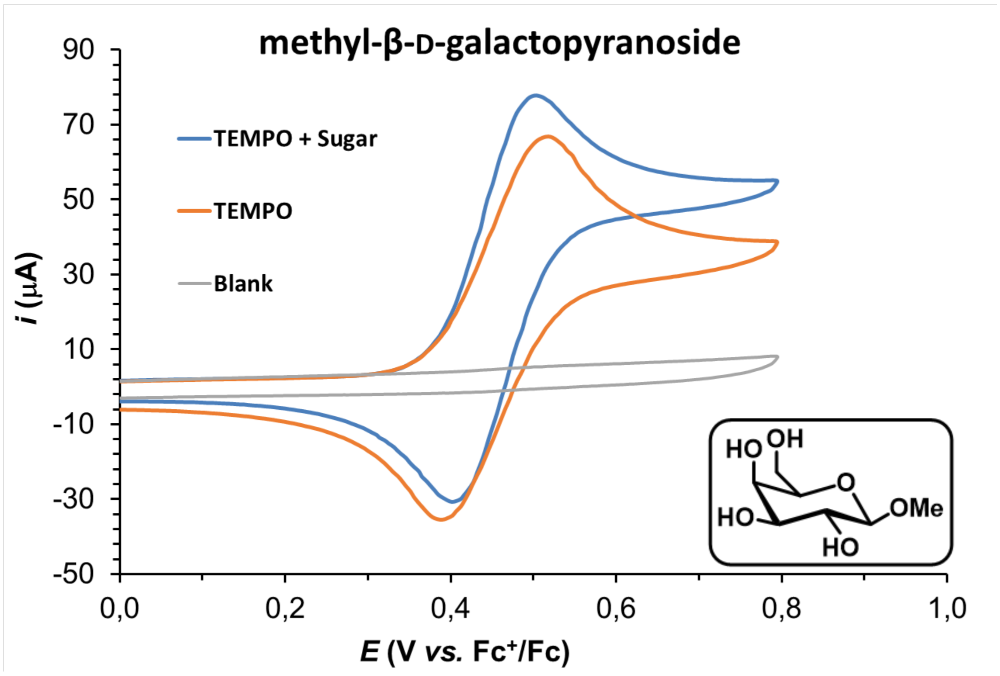

Figure S20: CV of 300 mM NH_4BF_4 (gray), 300 mM NH_4BF_4 + 2 mM TEMPO (orange), 300 mM NH_4BF_4 + 2 mM TEMPO + 100 mM methyl-β-D-galactopyranoside **3e** (blue) in the solvent mixture containing MeCN : pyridine in 9 : 1 ratio. Using a glassy carbon working electrode, a platinum wire counter electrode and a $Ag/AgNO_3$ reference electrode and converted to Fc^+/Fc with scan rate of 100 mV/s.

6.2.9 CV study of TEMPO with methyl-N-acetyl-α-D-glucosamine 3f in MeCN : pyridine 9 : 1

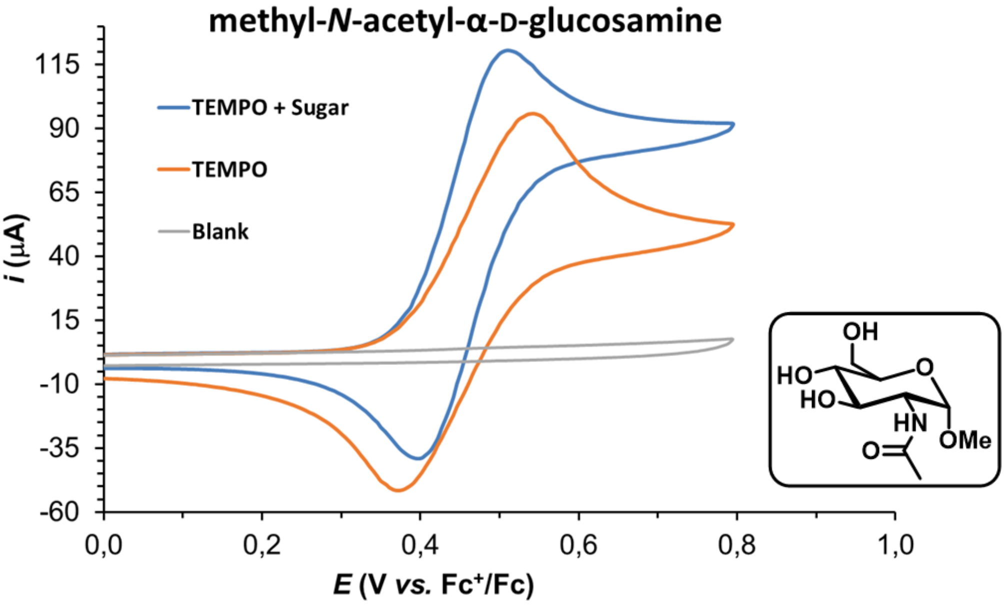

Figure S21: CV of 300 mM NH_4BF_4 (gray), 300 mM NH_4BF_4 + 2 mM TEMPO (orange), 300 mM NH_4BF_4 + 2 mM TEMPO + 100 mM 1-O-methyl-N-acetyl-α-D-glucosamine **3f** (blue) in the solvent mixture containing MeCN : pyridine in 9 : 1 ratio. Using a glassy carbon working electrode, a platinum wire counter electrode and a Ag/$AgNO_3$ reference electrode and converted to Fc^+/Fc with scan rate of 100 mV/s.

7. Time course experiment for the ammoxidation of methyl-α-D-glucopyranoside

Reactions were carried out in an undivided cell equipped with a graphite anode (Rod, 6.0 cm x Ø0.6 cm, ~ 3 cm was immersed in the solution), a platinum wire cathode (1.0 cm, spiral wire) and a Ag/$AgNO_3$ reference electrode. A mixture of methyl-α-D-glucopyranoside **1** (48 mg, 0.25 mmol, 1 equiv), ammonium tetrafluoroborate (52 mg, 0.5 mmol, 2 equiv) and TEMPO (12 mg, 0.075 mmol, 30 mol%) were added in 5 mL 9 : 1 MeCN : pyridine. Constant current electrolysis (4 mA, j = 0.7 mA/cm^2) was conducted for 840 minutes (charged passed = 8 F/mol) at room temperature, with a stirring speed of 1000 rpm. During electrolysis, an aliquot of the reaction mixture (50 µL) was drawn after addition of each Faraday. To this, 25 µL of a solution of 0.1 M 1, 3, 5-trimethoxy benzene in MeCN was added as an internal standard, diluted with CD_3OD and submitted to ^{1}H-NMR analysis.

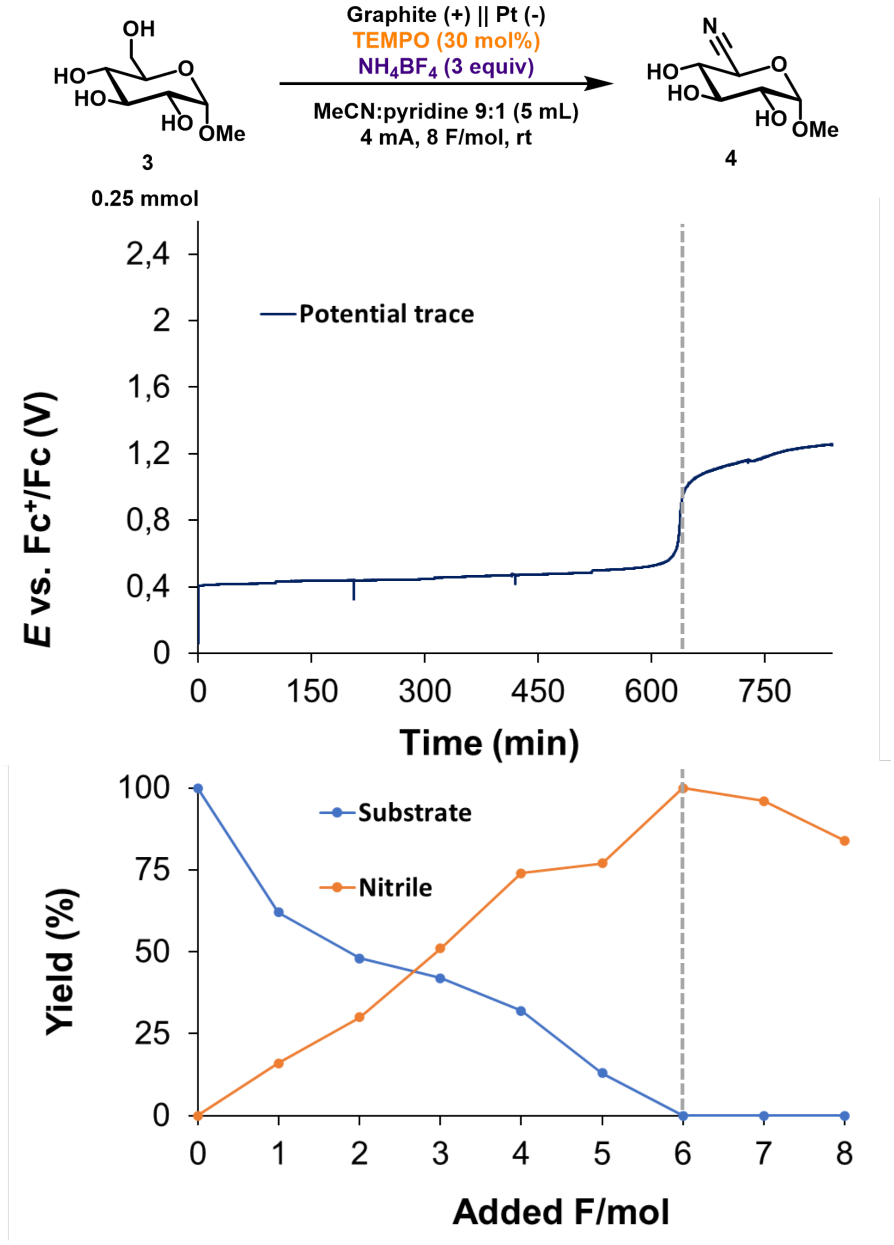

Figure S22: Time course for the oxidation of methyl-α-D-glucopyranoside **3**. Reaction conditions: methyl-α-D-glucopyranoside **3** (48 mg, 0.25 mmol, 1 equiv), ammonium tetrafluoroborate 52 mg, (0.5 mmol, 2 equiv) and TEMPO (12 mg, 0.075 mmol, 30 mol%) in 5 mL 9 : 1 MeCN : pyridine in an undivided cell with a graphite working electrode, Pt counter electrode and Ag/$AgNO_3$ reference electrode. Constant current electrolysis at 4 mA up to 8 F/mol at room temperature, with a stirring speed of 1000 rpm. qNMR yields based on ^{1}H-NMR with 1, 3, 5-trimethoxy benzene as an internal standard. Top: reaction scheme, middle: constant current plot at 4 mA, bottom: product yield plot.

8. Potential traces

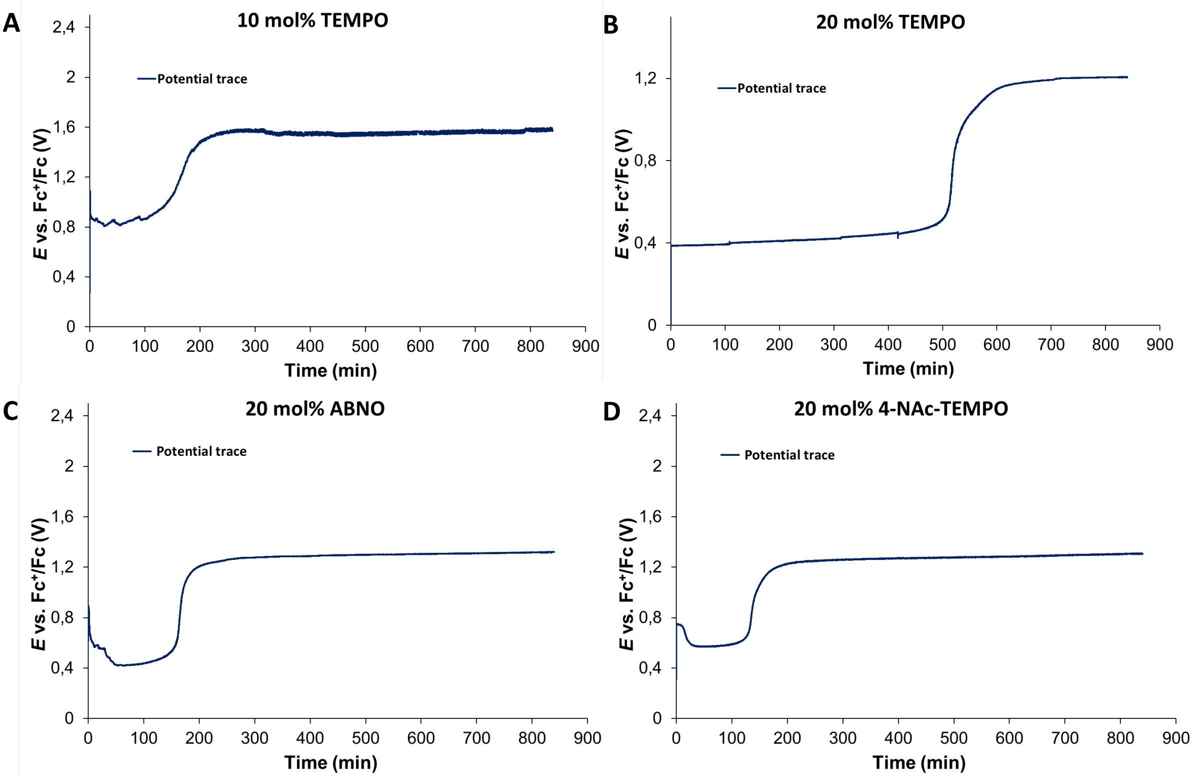

Figure S23: Potential trace of the electrochemical ammoxidation. Reaction conditions: methyl-α-D-glucopyranoside **3** (48 mg, 0.25 mmol, 1 equiv), ammonium tetrafluoroborate (52 mg, 0.5 mmol, 2 equiv) in 5 mL 9 : 1 MeCN : pyridine in an undivided cell with a graphite working electrode, Pt counter electrode and Ag/$AgNO_3$ reference electrode. Constant current electrolysis at 4 mA for 8 F/mol at room temperature. Using different mediators: A) 10 mol% TEMPO, B) 20 mol% TEMPO, C) 20 mol% ABNO, D) 20 mol% 4-NAc-TEMPO

9. Characterization data of the electrochemical oxidations

Cyano-methyl-α-D-glucopyranoside **4a**.

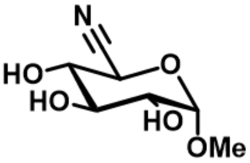

Prepared via the general procedure outlined in section 2, from methyl-α-D-glucopyranoside **3a** (48.5 mg, 0.25 mmol). The product was characterized by qNMR (93% yield) and the spectral data match with literature[3] values of the isolated product (**Figure S24**).

^{1}H-NMR (500 MHz, CD_3OD) δ 4.74 (d, *J* = 3.5 Hz, 1H), 4.36 (d, *J* = 9.9 Hz, 1H), 3.61 – 3.47 (m, 2H), 3.47 – 3.42 (m, 4H).

Cyano-methyl-α-D-glucopyranoside triacetate **7a**

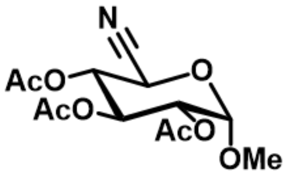

Product **4a** was isolated as its triacetate via the general procedure outlined in section 4.1. This afforded the product as a white solid (58.8 mg, 75%).

^{1}H-NMR (400 MHz, $CDCl_3$): δ 5.40 (dd, *J* = 10.1, 9.3 Hz, 1H), 5.27 (dd, *J* = 10.3, 9.3 Hz, 1H), 5.02 (d, *J* = 3.6 Hz, 1H), 4.86 (dd, *J* = 10.1, 3.6 Hz, 1H), 4.62 (dd, *J* = 10.3, 0.7 Hz, 1H), 3.48 (s, 3H), 2.10 (s, 3H), 2.08 (s, 3H), 2.03 (s, 3H).

^{13}C-NMR (101 MHz, $CDCl_3$): δ 170.0, 169.9, 169.0, 115.1, 97.5, 70.0, 69.3, 68.9, 58.9, 56.7, 20.7, 20.7, 20.5.

HRMS (ESI^+) calculated for $C_{13}H_{17}NO_8Na^+$ ($[M+Na]^+$): 338.0846, found: 338.0842.

MP: 133 °C

Cyano-methyl-β-D -glucopyranoside **4b**.

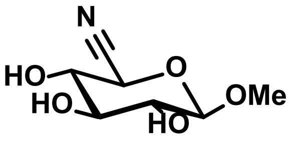

Prepared via the general procedure outlined in section 2, form methyl-α-D-glucopyranoside **3b** (48.5 mg, 0.25 mmol). The product was characterized by qNMR (92% yield) and the spectral match with literature[3] values of the isolated product (**Figure S26**).

^{1}H-NMR (400 MHz, CD_3OD) δ 4.28 – 4.23 (m, 2H, overlapping H1 and H5 coupling with *J* = 7.7 and *J* = 9.9 respectively), 3.55 – 3.47 (m, 4H), 3.35 (t, *J* = 9.2 Hz, 1H), 3.19 (dd, *J* = 9.2, 7.7 Hz, 1H).

Cyano-methyl-α-D-mannopyranoside **4c**.

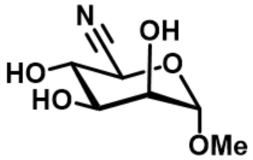

Prepared via the general procedure outlined in section 2, form methyl-α-D-glucopyranoside **3c** (48.5 mg, 0.25 mmol). Constant current electrolysis was conducted till addition of 8 F/mol. The product was characterized by qNMR (87% yield) and the spectral data match with literature[3] values of the isolated product (**Figure S28**).

^{1}H-NMR (500 MHz, CD_3OD) δ 4.69 (d, *J* = 1.8 Hz, 1H), 4.30 (d, *J* = 9.8 Hz, 1H), 3.87 (t, *J* = 9.6 Hz, 1H), 3.80 (dd, *J* = 3.2, 1.8 Hz, 1H), 3.61 (dd, *J* = 9.3, 3.2 Hz, 1H), 3.41 (s, 3H).

Cyano-methyl-α-D-galactopyranoside **4d**

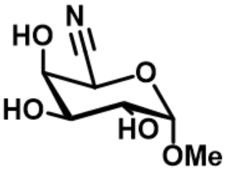

Prepared via the general procedure outlined in section 2, form methyl-α-D-galactopyranoside **3d** (48.5 mg, 0.25 mmol). The product was characterized by qNMR (81% yield) and the spectral data match with literature[3] values of the isolated product (**Figure S30**).

^{1}**H-NMR** (500 MHz, CD_3OD) δ 4.82 (d, *J* = 1.6 Hz, 1H), 4.77 (d, *J* = 3.3 Hz, 1H), 4.03 (dd, *J* = 3.1, 1.6 Hz, 1H), 3.81 – 3.73 (m, 2H), 3.44 (s, 3H).

Cyano-methyl-β-D -galactopyranoside **4e**.

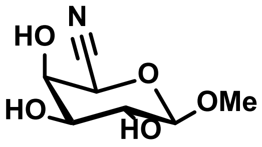

Prepared via the general procedure outlined in section 2, form methyl-β-D-galactopyranoside **3e** (48.5 mg, 0.25 mmol). Constant current electrolysis was conducted till addition of 8 F/mol. The product was characterized by qNMR (89% yield) and the spectral data match with literature[3] values of the isolated product (**Figure S32**).

^{1}**H-NMR** (500 MHz, CD_3OD) δ 4.69 (d, *J* = 1.5 Hz, 1H), 4.21 (d, *J* = 7.3 Hz, 1H), 3.98 (q, *J* = 1.5 Hz, 1H), 3.55-3.51 (m, 5H).

Cyano-methyl-N-acetyl-α-D-glucosamine **4f**

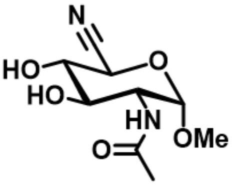

Prepared via the general procedure outlined in section 2, form methyl N-acetyl-α-D-glucosamine **3f** (58.8 mg, 0.25 mmol). Constant current electrolysis was conducted till addition of 8 F/mol. The product was characterized by qNMR (70% yield) and the spectral data match with literature[3] values of the isolated product (**Figure S34**).

^{1}**H-NMR** (500 MHz, CD_3OD) δ 4.74 (d, *J* = 3.4 Hz, 1H), 4.40 (d, *J* = 9.3 Hz, 1H), 3.97 (dd, *J* = 10.1, 3.4 Hz, 1H), 3.65 – 3.56 (m, 2H), 3.43 (s, 3H), 2.00 (s, 3H).

Cyano-allyl β-D-fructopyranoside **4g**

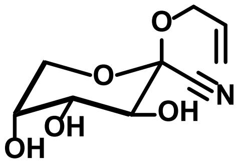

Prepared via the general procedure outlined in section 2, form allyl β-D-fructopyranoside **3g** (55.1 mg, 0.25 mmol), which was synthesized according to known literature procure.[4] The product was characterized by qNMR (82% yield) (**Figure S36**).

^{1}H-NMR (400 MHz, CD_3OD) δ 6.09 – 5.88 (m, 1H), 5.41 (dq, *J* = 17.2, 1.7 Hz, 1H), 5.24 (dq, *J* = 10.5, 1.4 Hz, 1H), 4.29 – 4.18 (m, 2H), 3.95 (d, *J* = 9.8 Hz, 1H), 3.89 (dt, *J* = 3.4, 1.7 Hz, 1H), 3.79 – 3.72 (m, 3H).

Cyano-allyl β-D-fructopyranoside triacetate **7g**

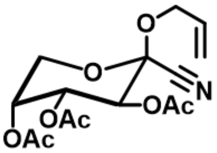

Product **4g** was isolated as its triacetate via the general procedure outlined in section 4.2. This afforded the product as a colorless oil (54.4 mg, 64%).

^{1}H-NMR (400 MHz, $CDCl_3$) δ 5.96 (ddt, *J* = 16.4, 10.8, 5.6 Hz, 1H), 5.68 (d, *J* = 10.5 Hz, 1H), 5.46 – 5.24 (m, 4H), 4.39 – 4.19 (m, 2H), 3.86 (t, *J* = 1.8 Hz, 2H), 2.19 (s, 3H), 2.15 (s, 3H), 2.00 (s, 3H).

^{13}C-NMR (101 MHz, $CDCl_3$) δ 170.3, 169.9, 169.3, 131.6, 119.1, 113.6, 95.3, 68.4, 68.1, 67.2, 67.2, 62.6, 21.0, 20.7, 20.6.

HRMS (ESI^+) calculated for $C_{15}H_{19}NO_8Na^+$ ($[M+Na]^+$): 364.1003, found: 364.1003.

Cyanoethyl-cyano-β-fructopyranoside **4h**

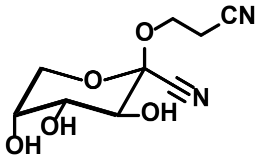

Prepared via the general procedure outlined in section 2, using only 5 mA constant current, form cyanoethyl-β-fructopyranoside **3h** (58.3 mg, 0.25 mmol), which was synthesized according to known literature procure [5]. The product was characterized by qNMR (79%) (**Figure S38**).

^{1}H-NMR (400 MHz,CD_3OD) δ 4.01 – 3.73 (m, 7H), 2.88 (td, *J* = 5.5, 2.1 Hz, 2H).

Cyanoethyl-cyano-β-fructopyranoside triacetate **7h**

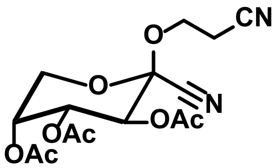

Product **4h** was isolated as its triacetate via the general procedure outlined in section 4.2. This afforded the product as a white solid (48.5 mg, 55%).

^{1}H-NMR (400 MHz, $CDCl_3$) δ 5.68 (d, *J* = 10.4 Hz, 1H), 5.37 (dt, *J* = 3.4, 1.8 Hz, 1H), 5.28 (dd, *J* = 10.4, 3.4 Hz, 1H), 4.06 – 3.83 (m, 4H), 2.90 – 2.67 (m, 2H), 2.18 (s, 3H), 2.15 (s, 3H), 2.00 (s, 3H).

^{13}C-NMR (101 MHz, $CDCl_3$) δ 170.2, 169.7, 169.2, 116.8, 113.0, 95.4, 68.0, 67.8, 66.8, 63.0, 61.1, 21.0, 20.6, 20.6, 18.5.

HRMS (ESI$^+$) calculated for $C_{15}H_{18}N_2O_8NH_4^+$ ($[M+NH_4]^+$): 372.1401, found: 372.1404.

MP: 138 °C

Dicyano-*n*-dodecyl β-D-maltoside **4i**

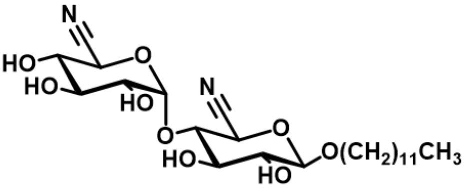

Prepared via the general procedure outlined in section 2 using twice the number of equivalents for all added reagents and only 5mA constant current, from *n*-dodecyl β-D-maltoside **3i** (127.7 mg, 0.25 mmol). The product was characterized by qNMR (85% yield) and isolated (**Figure S50**). The crude mixture was concentrated under reduced pressure, dissolved in EtOAc and washed with 1 M HCl_{aq}, dried over $MgSO_4$ and evaporated under reduced pressure. The product was isolated as an off-white solid (102.6 mg, 82%), in 85% purity.

^{1}H-NMR (400 MHz, CD_3OD) δ 5.46 (d, *J* = 3.2 Hz, 1H), 4.64 (d, *J* = 9.3 Hz, 1H), 4.51 (d, *J* = 10.0 Hz, 1H), 4.36 (d, *J* = 7.6 Hz, 1H), 3.90 – 3.75 (m, 2H), 3.64 – 3.47 (m, 5H), 3.26 (dd, *J* = 9.3, 7.6 Hz, 1H), 1.63 (p, *J* = 6.6 Hz, 2H), 1.30 (d, *J* = 5.0 Hz, 18H), 0.90 (t, *J* = 6.6 Hz, 3H).

^{13}C-NMR (101 MHz, CD_3OD) δ 118.3, 118.1, 104.8, 101.9, 80.3, 77.0, 74.1, 73.9, 73.2, 72.7, 71.5, 64.6, 64.4, 33.0, 30.8, 30.7, 30.7, 30.7, 30.6, 30.5, 30.4, 27.0, 23.7, 14.4.

HRMS (ESI$^-$) calculated for $C_{24}H_{39}N_2O_9^-$ ($[M-H]^-$): 499.2661, found: 499.2663.

MP: decomposition at 150 °C

Cyano-sucralose **4j**

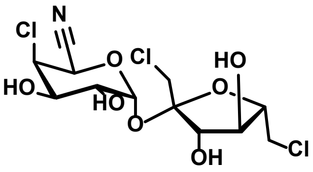

Prepared via the general procedure outlined in section 2, from sucralose **3j** (99.4 mg, 0.25 mmol). Constant current electrolysis was conducted till addition of 8 F/mol. The product was characterized by qNMR (93% yield) and isolated (**Figure S52**). The mixture was concentrated under reduced pressure. The concentrated reaction mixture was diluted with EtOAc and washed with once with an aqueous copper sulfate solution and once with brine, dried over Na_2SO_4 and evaporated under reduced pressure. The product was isolated as an off-white oil (73.3 mg, 75%), with 88% purity.

^{1}H-NMR (400 MHz, CD_3OD) δ 5.50 (d, *J* = 2.1 Hz, 1H), 5.49 (d, *J* = 3.6 Hz, 1H), 4.52 (dd, *J* = 3.6, 2.1 Hz, 1H), 4.33 (d, *J* = 7.6 Hz, 1H), 4.05 (dd, *J* = 9.5, 3.6 Hz, 1H), 4.00 – 3.89 (m, 2H), 3.87 – 3.75 (m, 3H), 3.73 (d, *J* = 1.7 Hz, 2H).

^{13}C-NMR (101 MHz, CD_3OD) δ 116.6, 105.2, 93.9, 83.6, 77.6, 77.0, 68.8, 68.7, 63.7, 62.3, 45.7, 45.1.

HRMS (ESI^-) calculated for $C_{12}H_{15}Cl_3NO_7^-$ ($[M-H]^-$): 389.9920, found: 389.9908.

10. NMR and HRMS spectra

10.1 qNMR spectra compared to isolated product and crude NMR spectra

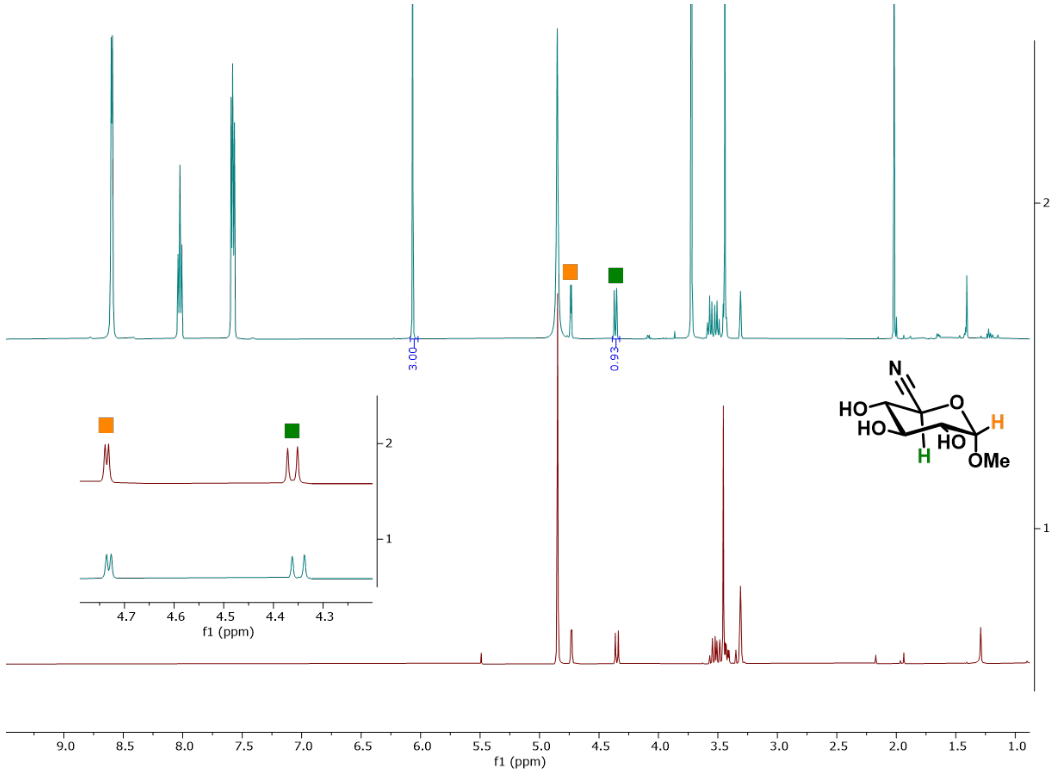

Figure S24: top: crude ^{1}H-NMR (500 MHz) in CD_3OD for electrochemical ammoxidation with trimethoxy benzene as internal standard. Bottom: ^{1}H-NMR in CD_3OD of isolated product **4a** from the literature.[3]

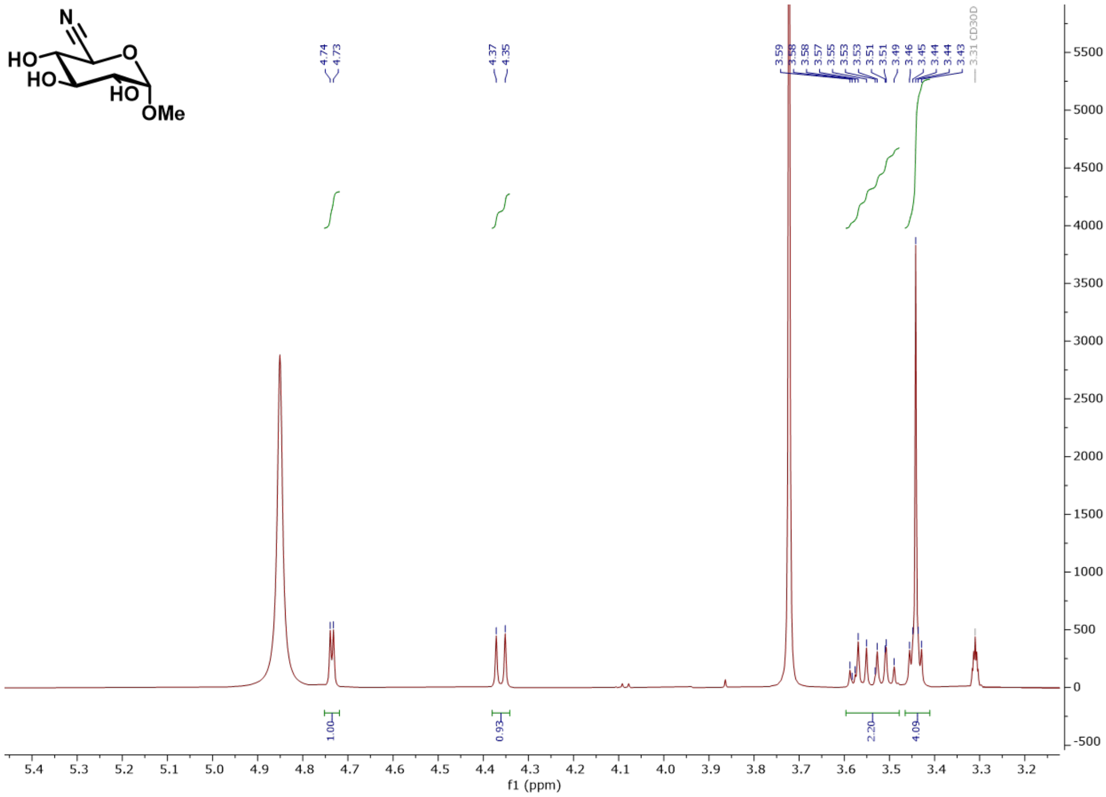

Figure S25: Zoomed in ^{1}H-NMR (500 MHz) spectrum in CD_3OD of crude of product **4a.**

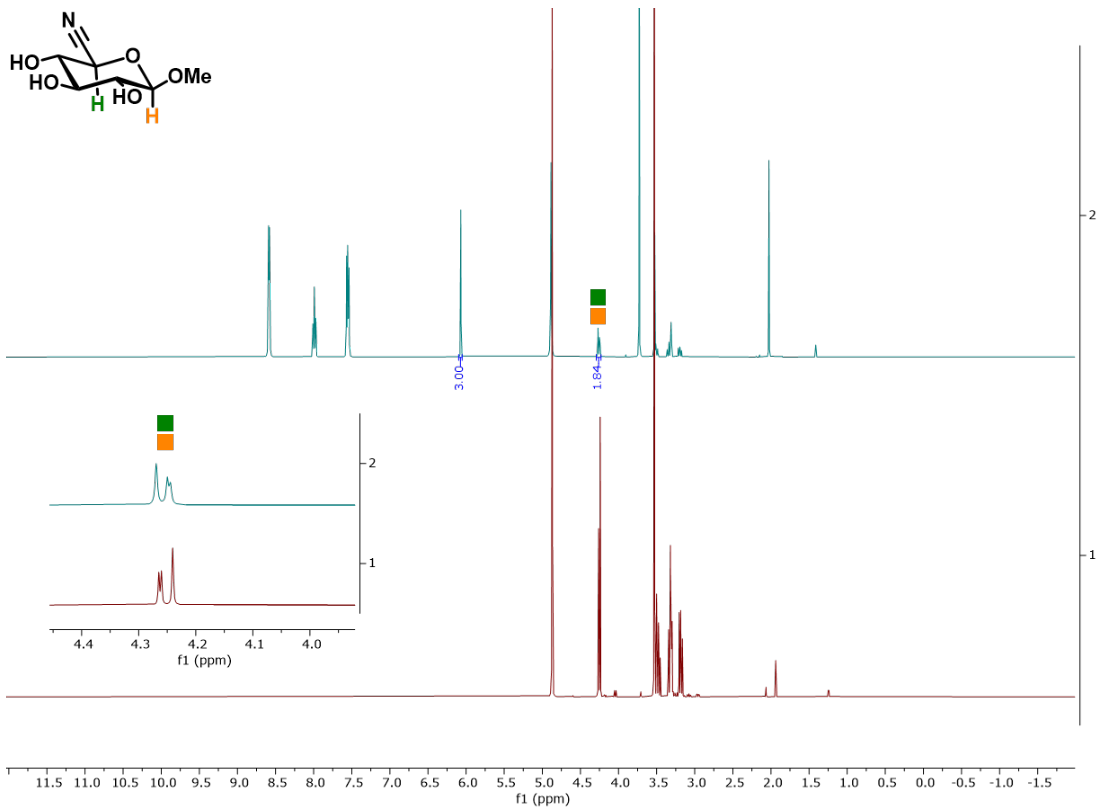

Figure S26*:* top: crude ^{1}H-NMR (500 MHz) in CD_3OD for electrochemical ammoxidation with trimethoxy benzene as internal standard. Bottom: ^{1}H-NMR in CD_3OD of isolated product **4b** from literature.[3]

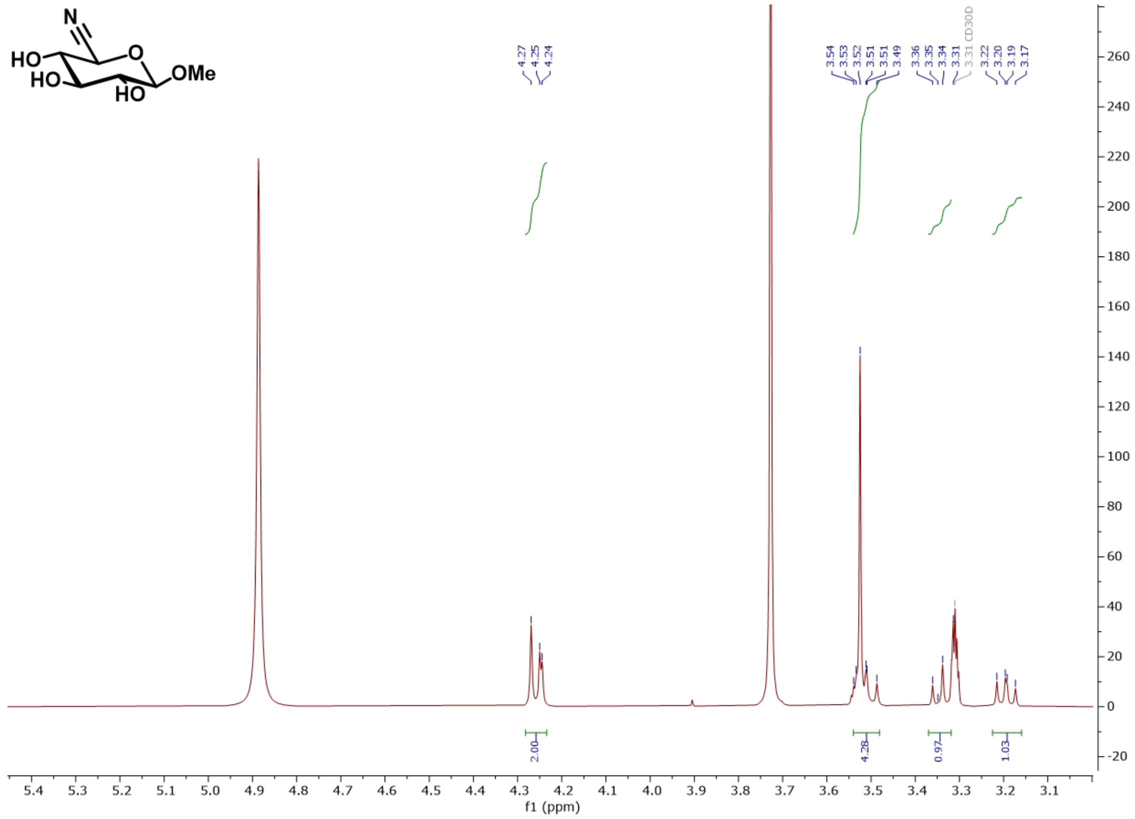

Figure S27*:* Zoomed in ^{1}H-NMR (500 MHz) spectrum in CD_3OD of crude of product **4b.**

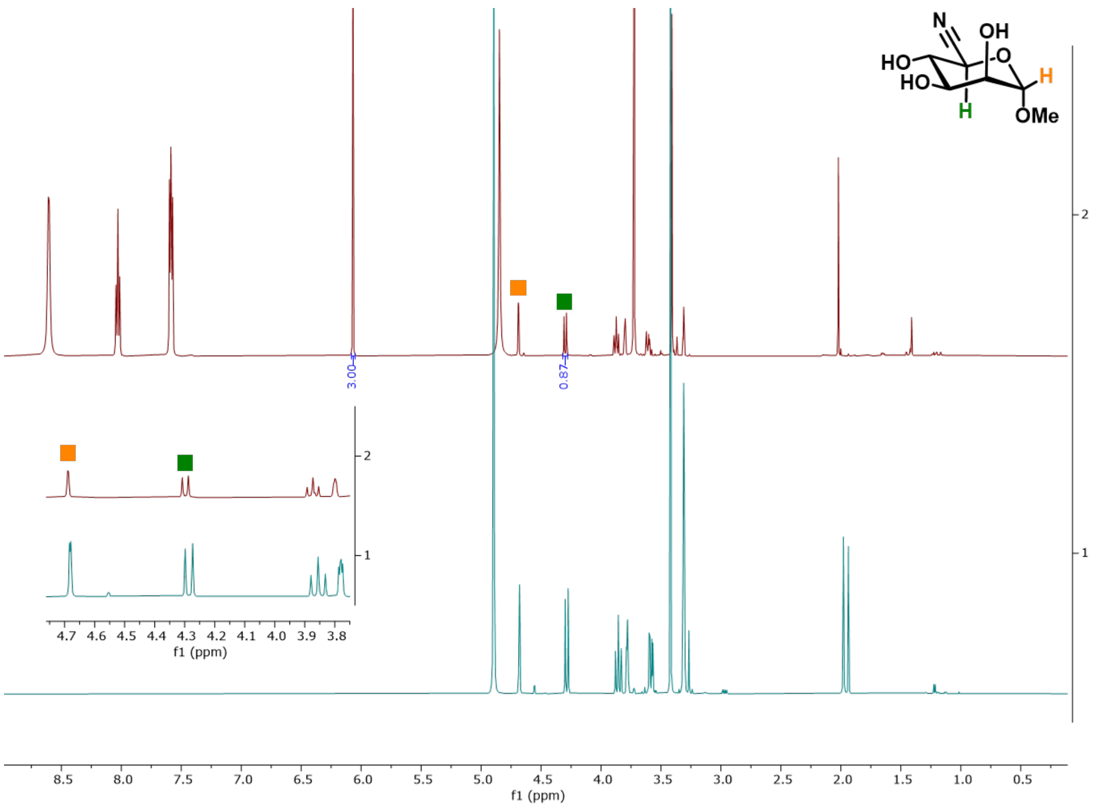

Figure S28: top: crude ^{1}H-NMR (500 MHz) in CD_3OD for electrochemical ammoxidation with trimethoxy benzene as internal standard. Bottom: ^{1}H-NMR in CD_3OD of isolated product **4c** from literature.[3]

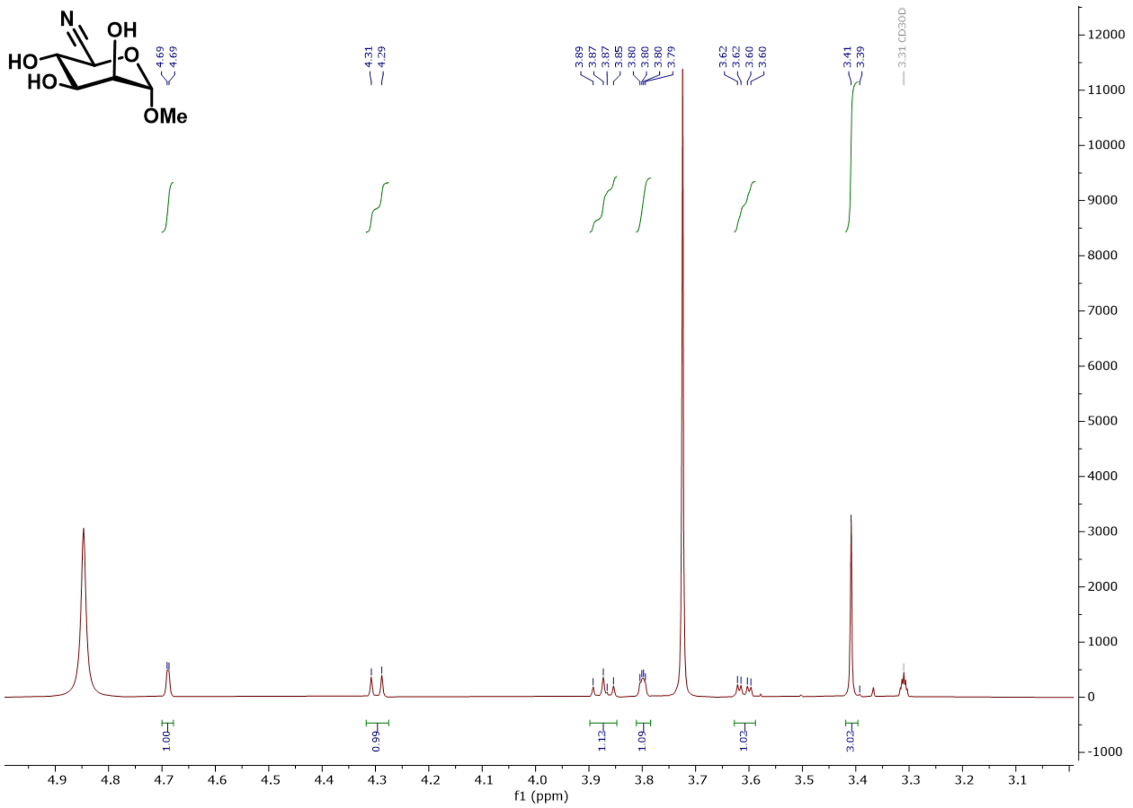

Figure S29: Zoomed in ^{1}H-NMR (500 MHz) spectrum in CD_3OD of crude of product **4c.**

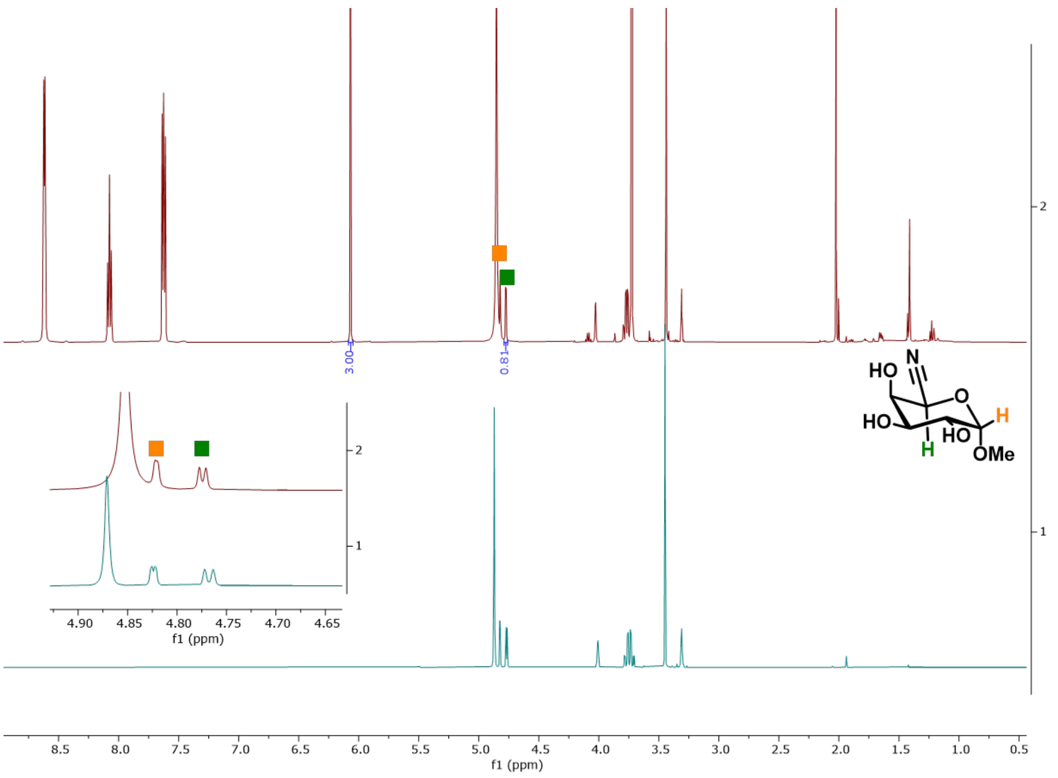

Figure S30: top: crude ^{1}H-NMR (500 MHz) in CD_3OD for electrochemical ammoxidation with trimethoxy benzene as internal standard. Bottom: ^{1}H-NMR in CD_3OD of isolated product **4d** from literature.[3]

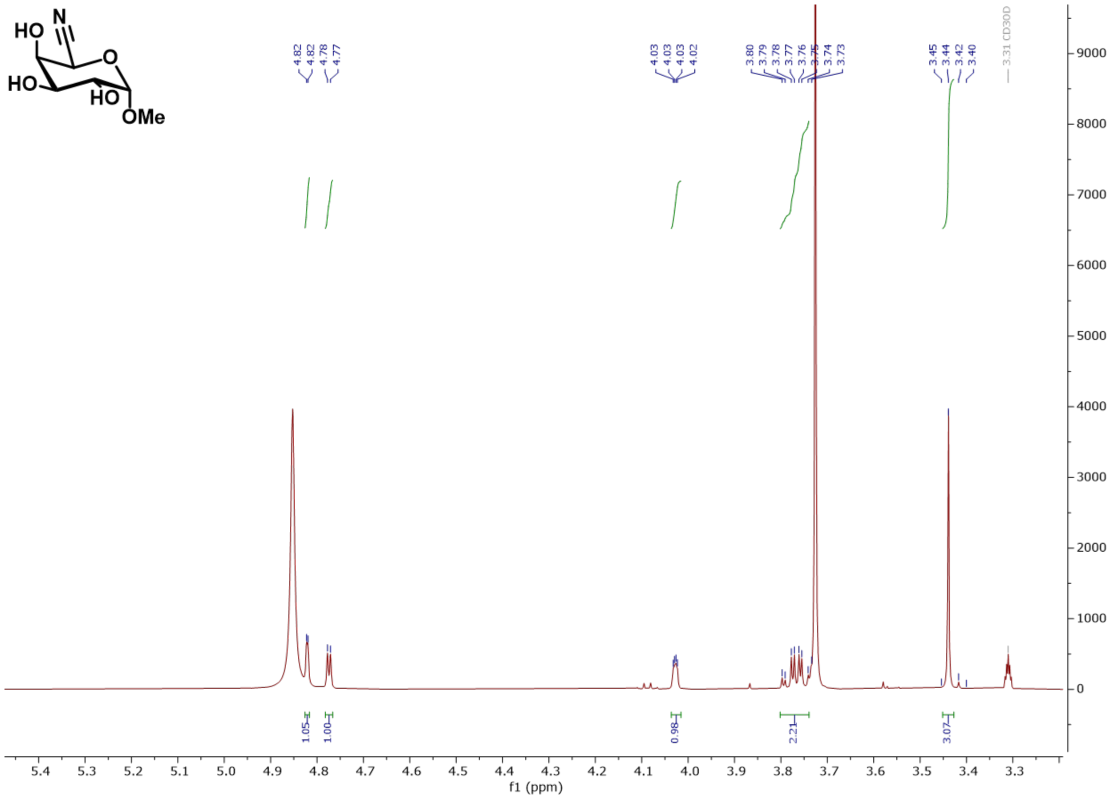

Figure S31: Zoomed in ^{1}H-NMR (500 MHz) spectrum in CD_3OD of crude of product **4d.**

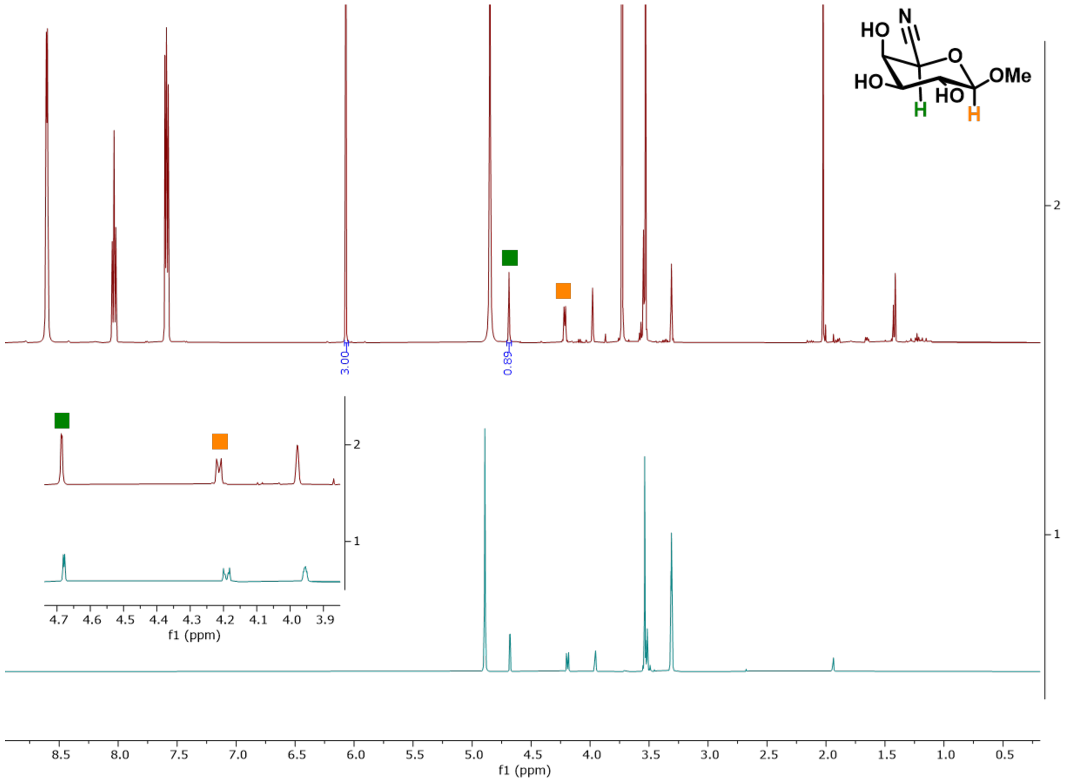

Figure S32*:* top: crude ^{1}H-NMR (500 MHz) in CD_3OD for electrochemical ammoxidation with trimethoxy benzene as internal standard. Bottom: ^{1}H-NMR in CD_3OD of isolated product **4e** from literature.[3]

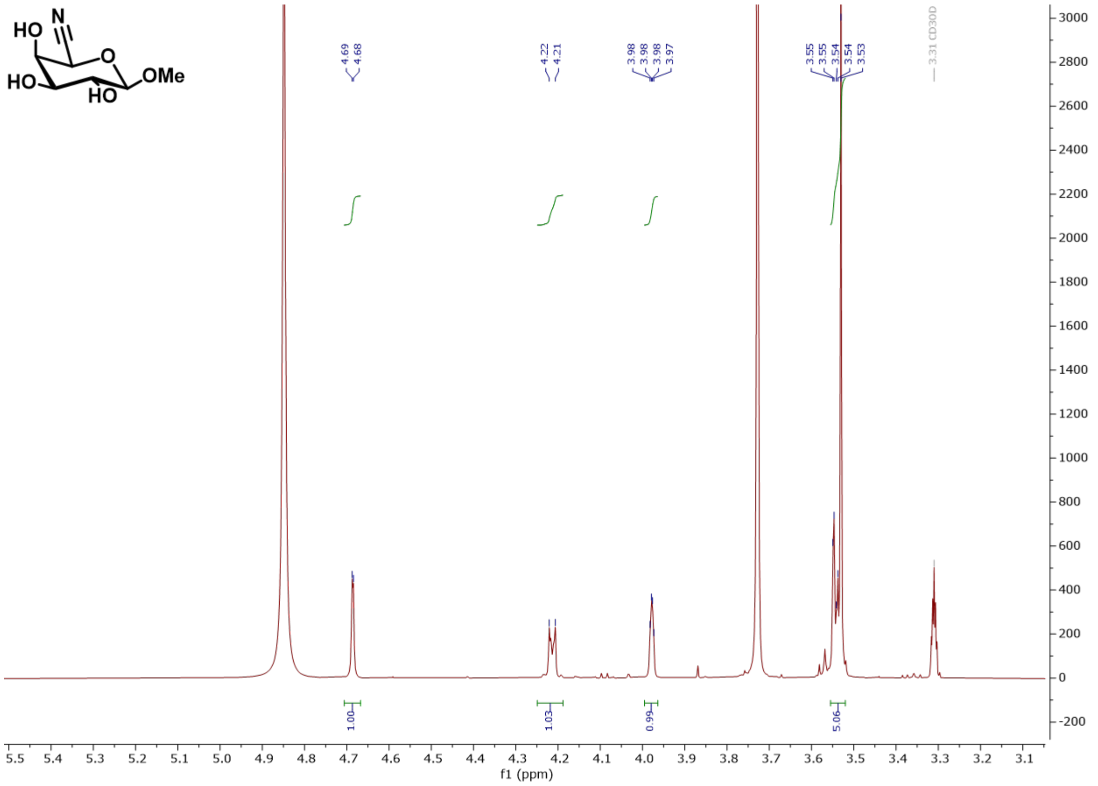

Figure S33*:* Zoomed in ^{1}H-NMR (500 MHz) spectrum in CD_3OD of crude of product **4e.**

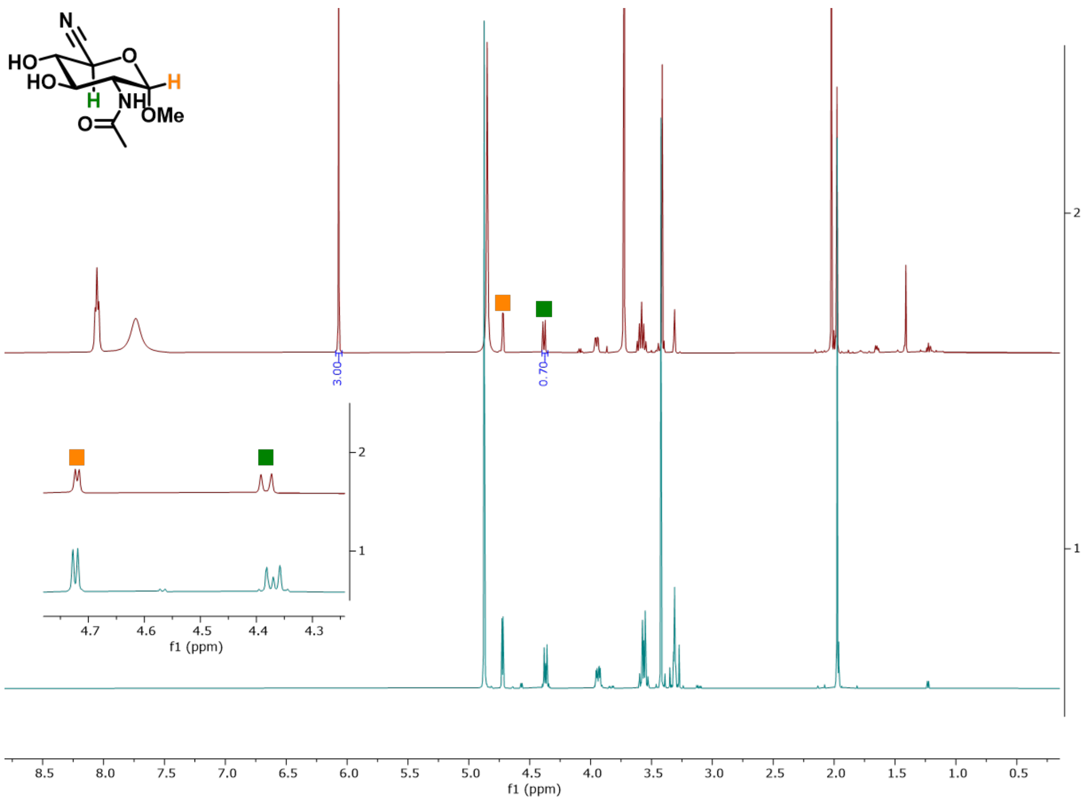

Figure S34*:* top: crude ^{1}H-NMR (500 MHz) in CD_3OD for electrochemical ammoxidation with trimethoxy benzene as internal standard. Bottom: ^{1}H-NMR in CD_3OD of isolated product **4f** from literature.[3]

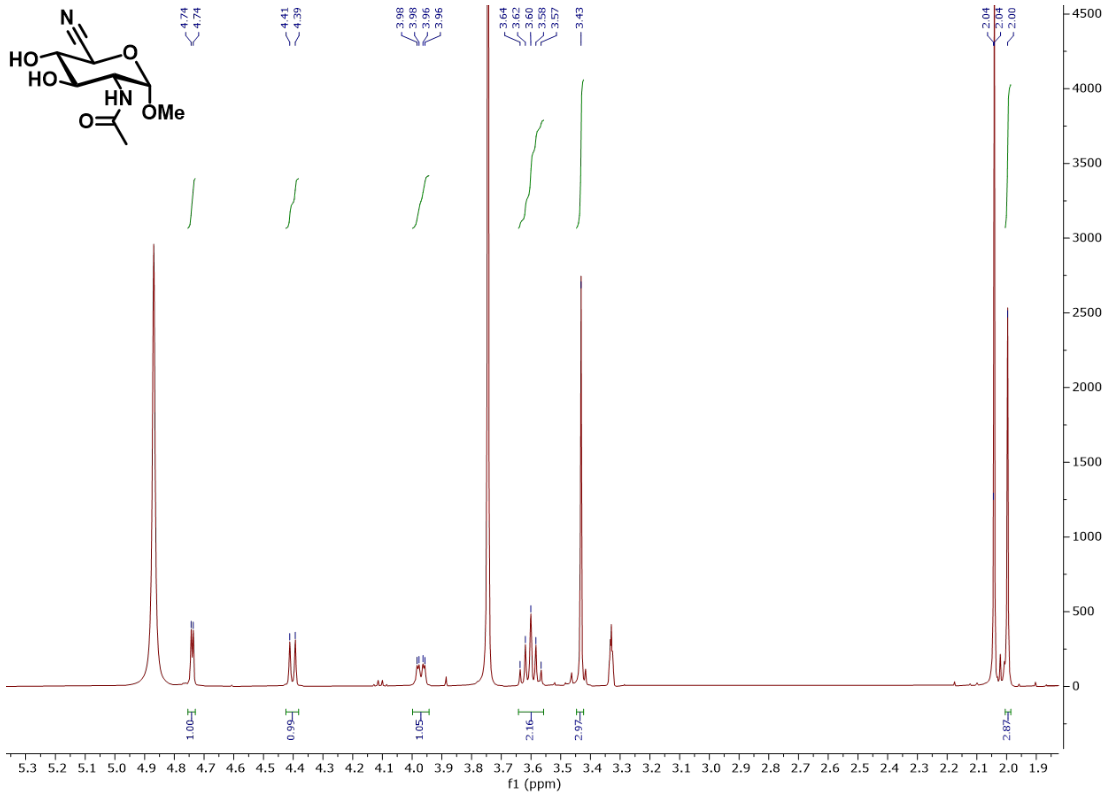

Figure S35*:* Zoomed in ^{1}H-NMR (500 MHz) spectrum in CD_3OD of crude of product **4f.**

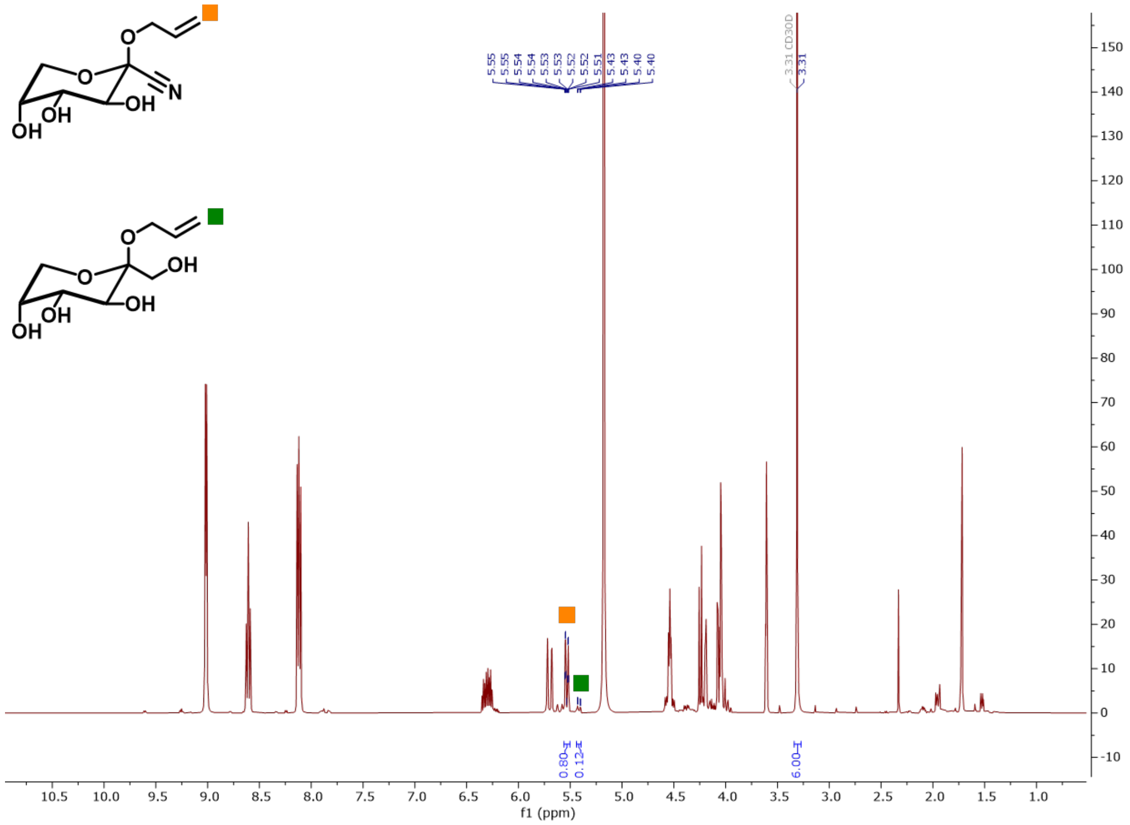

Figure S36: Crude ^{1}H-NMR (400 MHz) of **4g** in CD_3OD for electrochemical ammoxidation with dimethyl sulfone as internal standard.

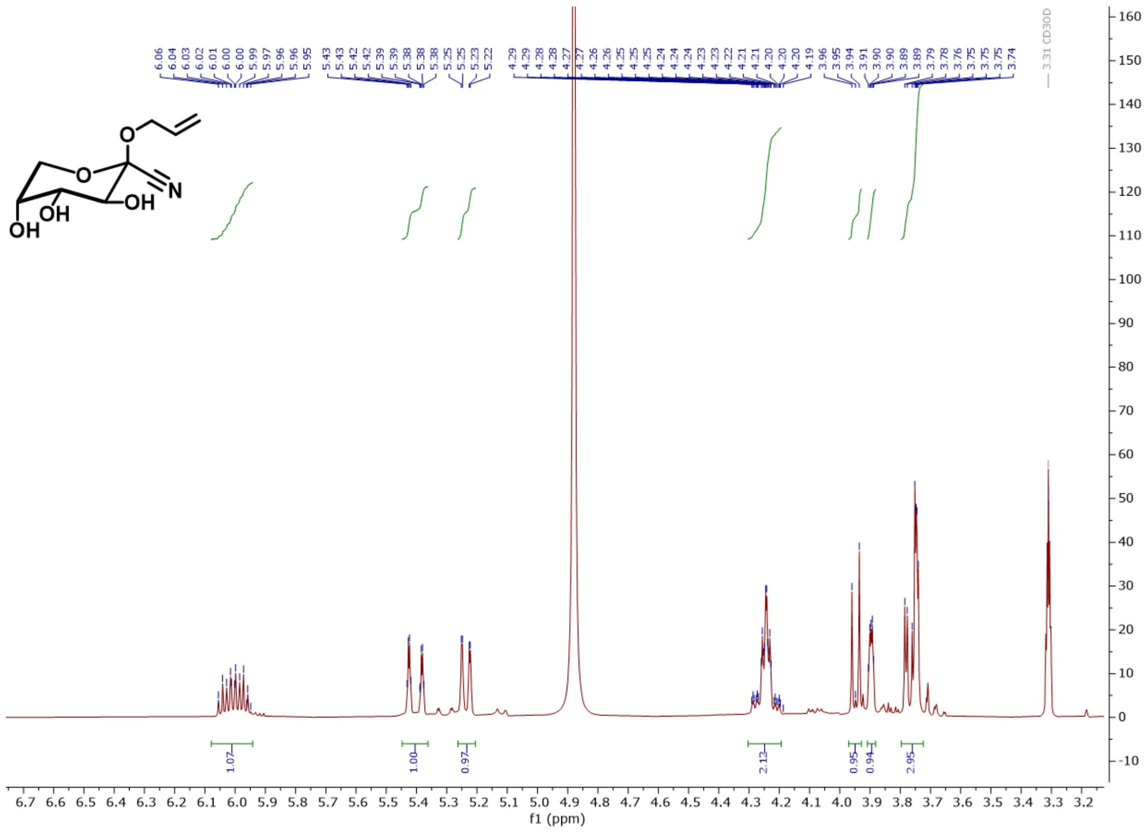

Figure S37*:* Zoomed in ^{1}H-NMR (400 MHz) spectrum in CD_3OD of crude of product **4g.**

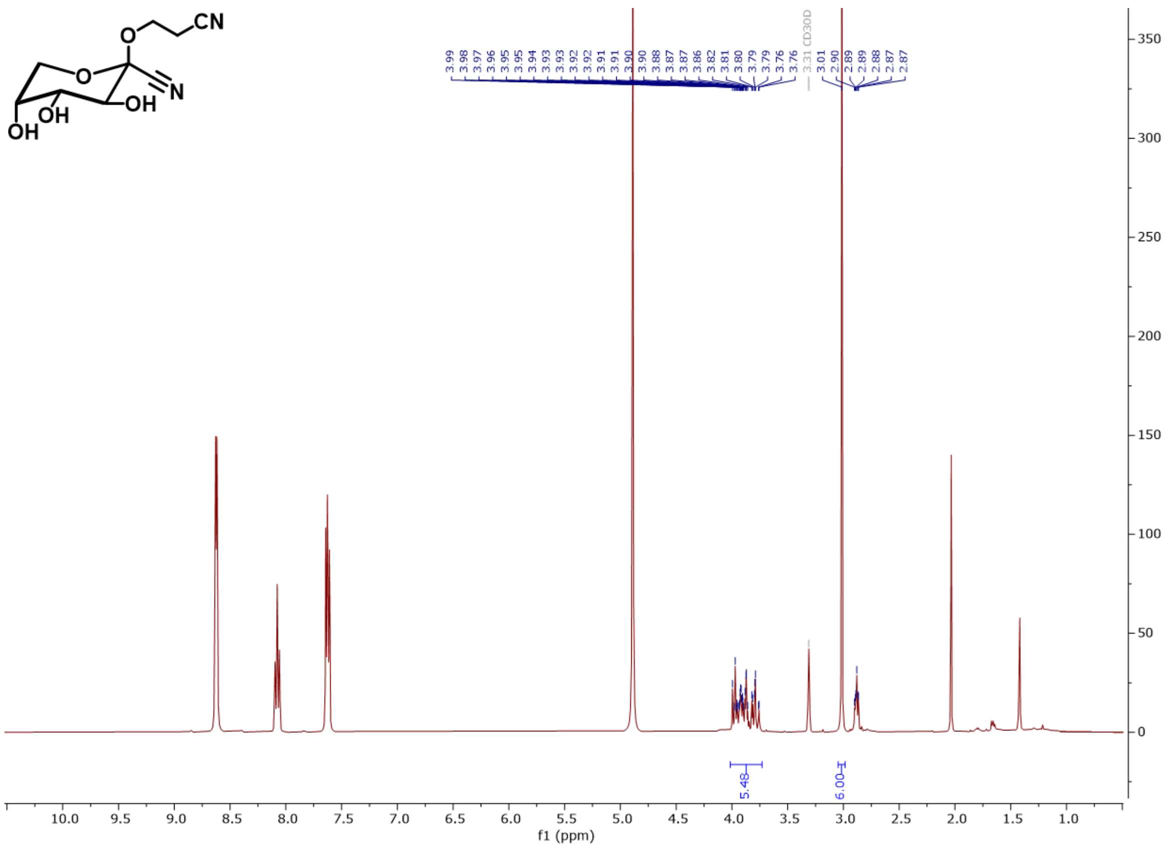

Figure S38: Crude ^{1}H-NMR (400 MHz) of **4h** in CD_3OD for electrochemical ammoxidation with dimethyl sulfone as internal standard.

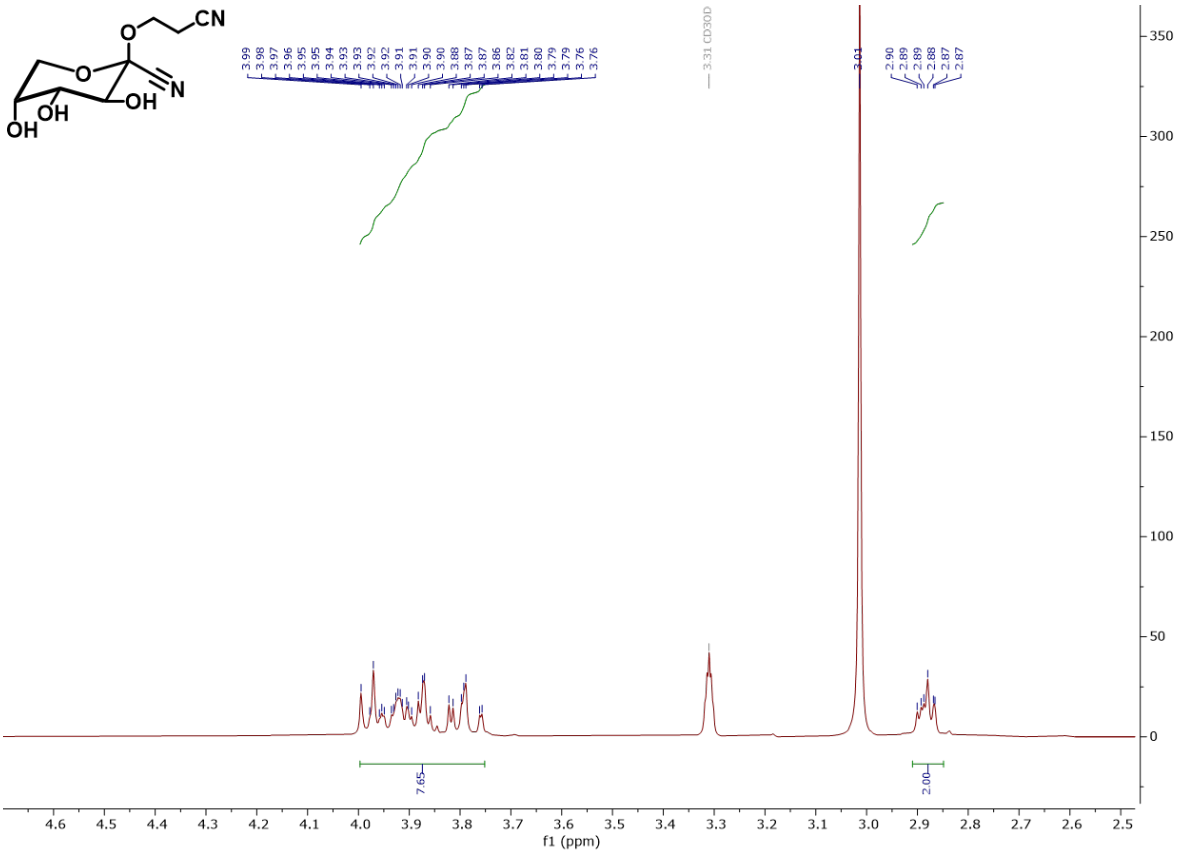

Figure S39: Zoomed in ^{1}H-NMR (400 MHz) spectrum in CD_3OD of crude of product**4.**

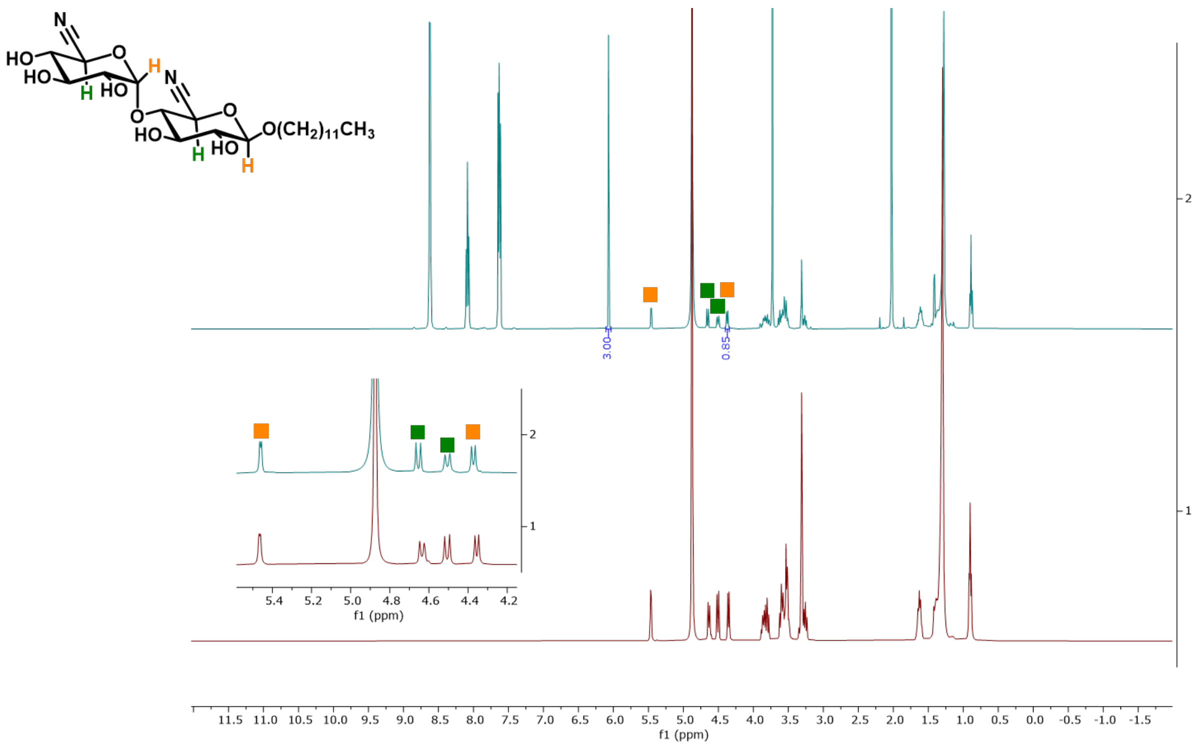

Figure S40: top: crude ^{1}H-NMR (400 MHz) in CD_3OD for electrochemical ammoxidation with trimethoxy benzene as internal standard. Bottom: ^{1}H-NMR in CD_3OD of isolated product **4i**.

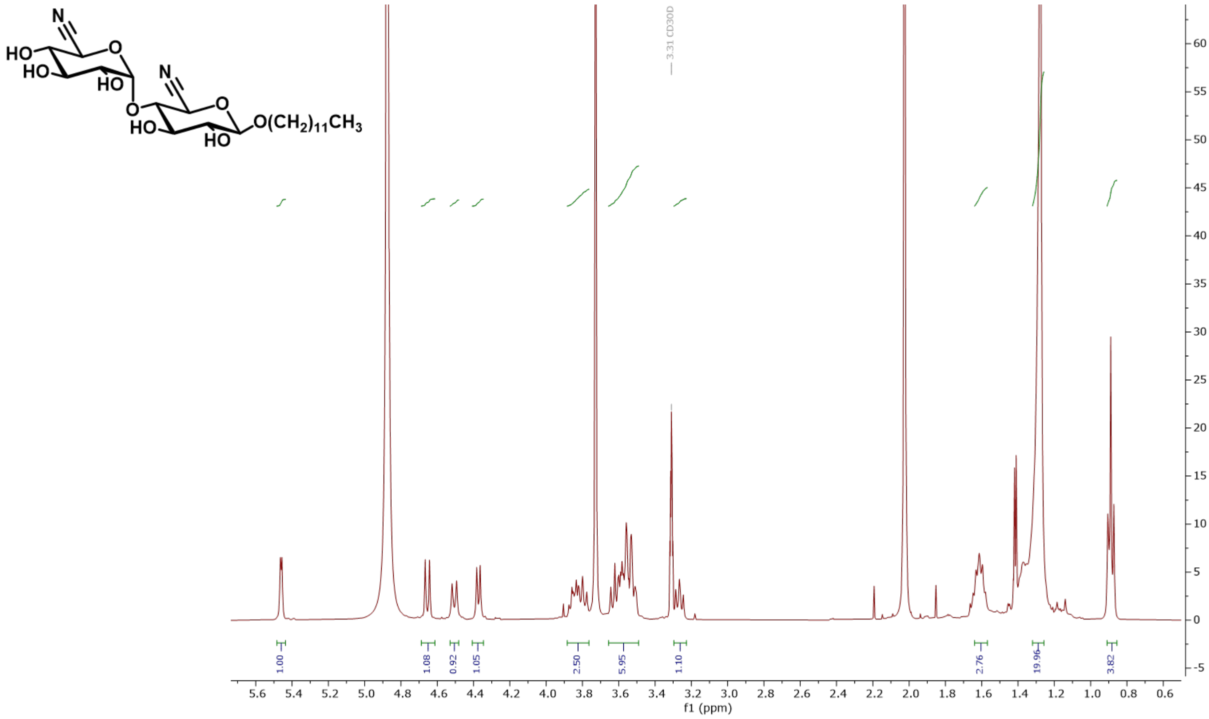

Figure S41*:* Zoomed in ^{1}H-NMR (400 MHz) spectrum in CD_3OD of crude of product **4i.**

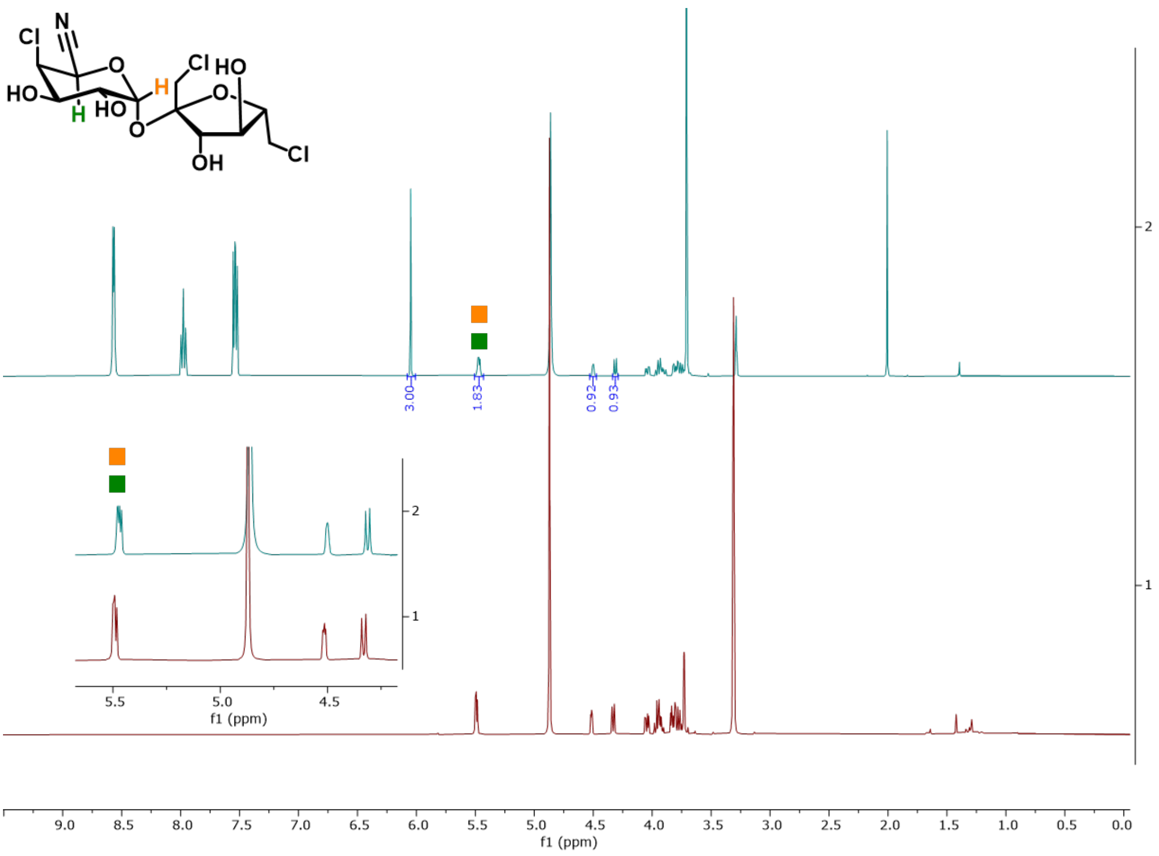

Figure S42: top: crude ^{1}H-NMR (400 MHz) in CD_3OD for electrochemical ammoxidation with trimethoxy benzene as internal standard. Bottom: ^{1}H-NMR in CD_3OD of isolated product **4j.**

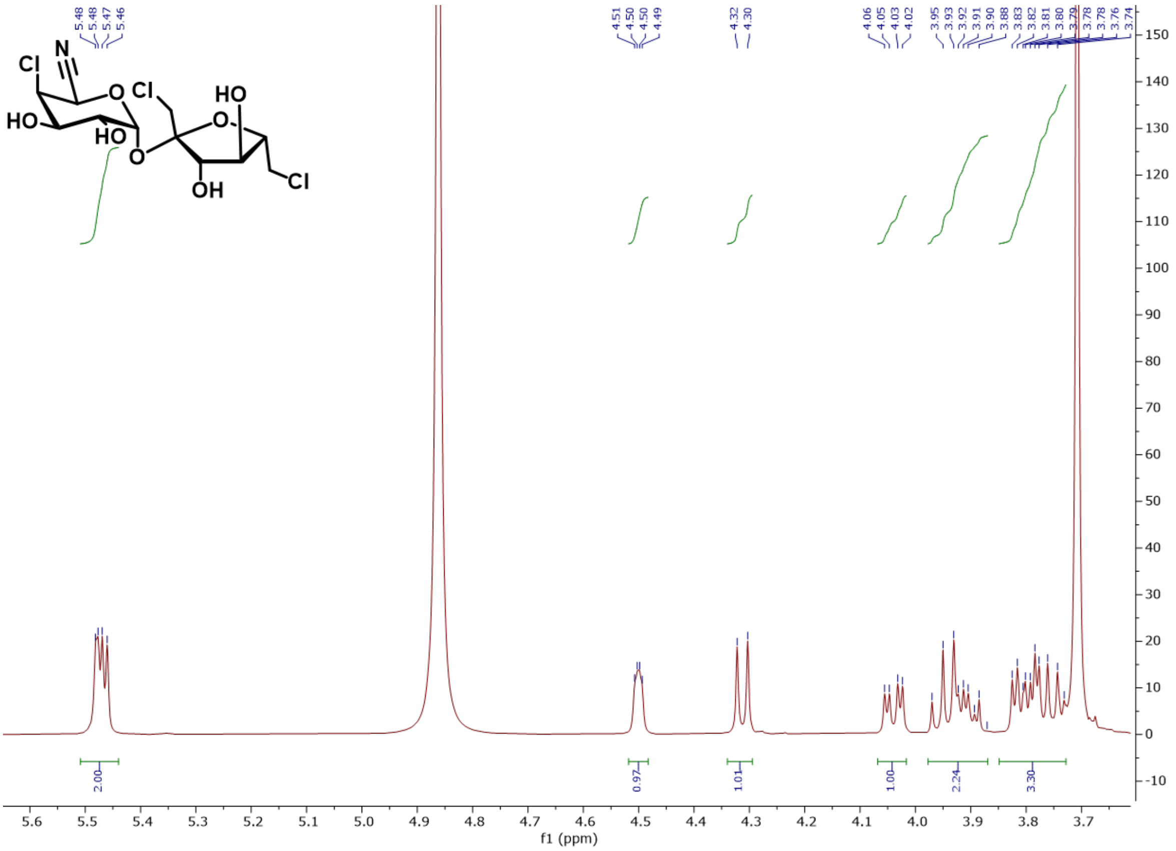

Figure S43: Zoomed in ^{1}H-NMR (400 MHz) spectrum in CD_3OD of crude of product **4j.**

10.2 Isolated ^{1}H and ^{13}C-NMR spectra

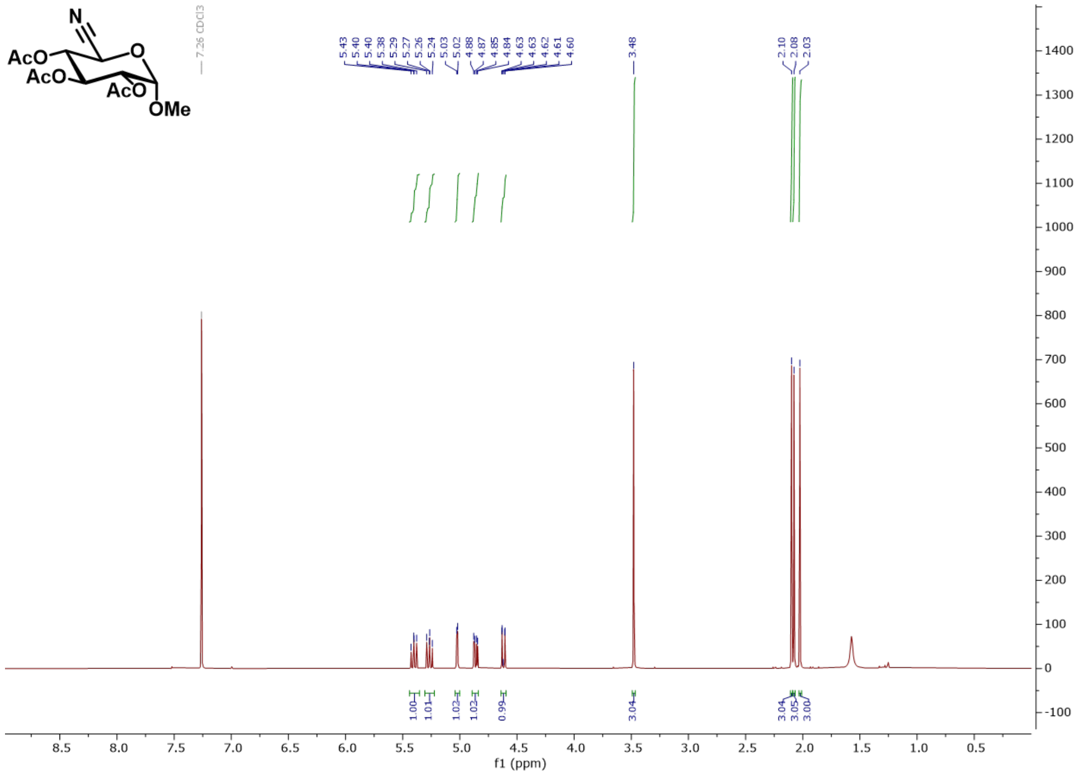

Figure S44*:* ^{1}H-NMR (400 MHz) spectrum in $CDCl_3$ of product **7a.**

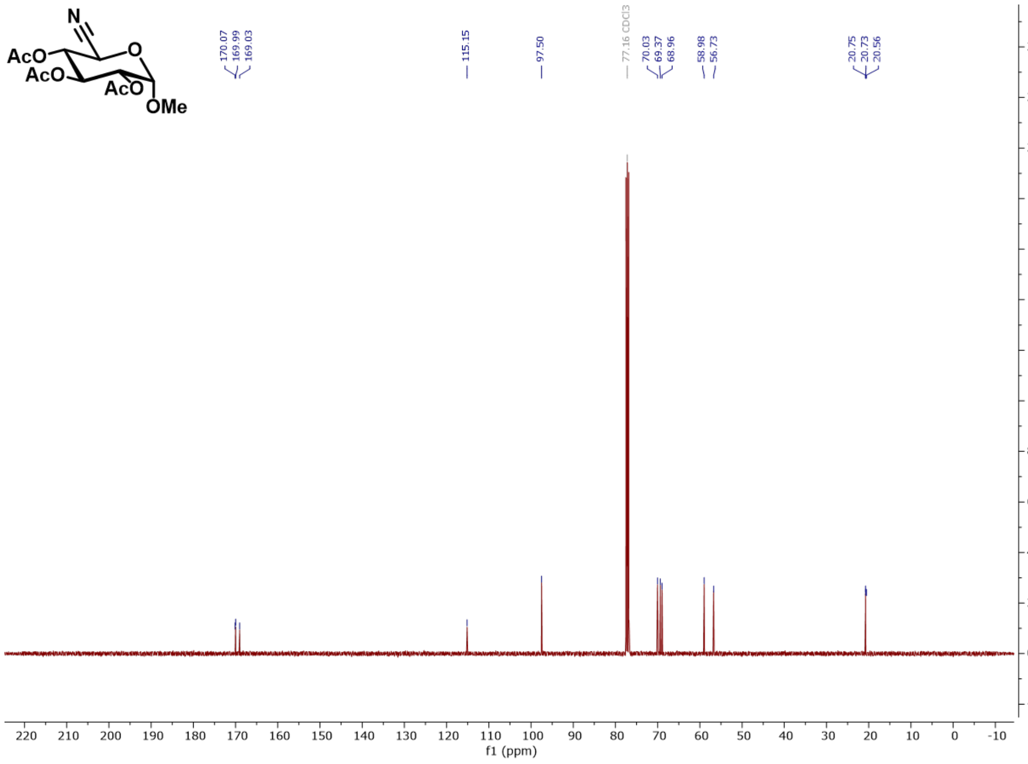

Figure S45: ^{13}C-NMR (101 MHz) spectrum in $CDCl_3$ of product **7a.**

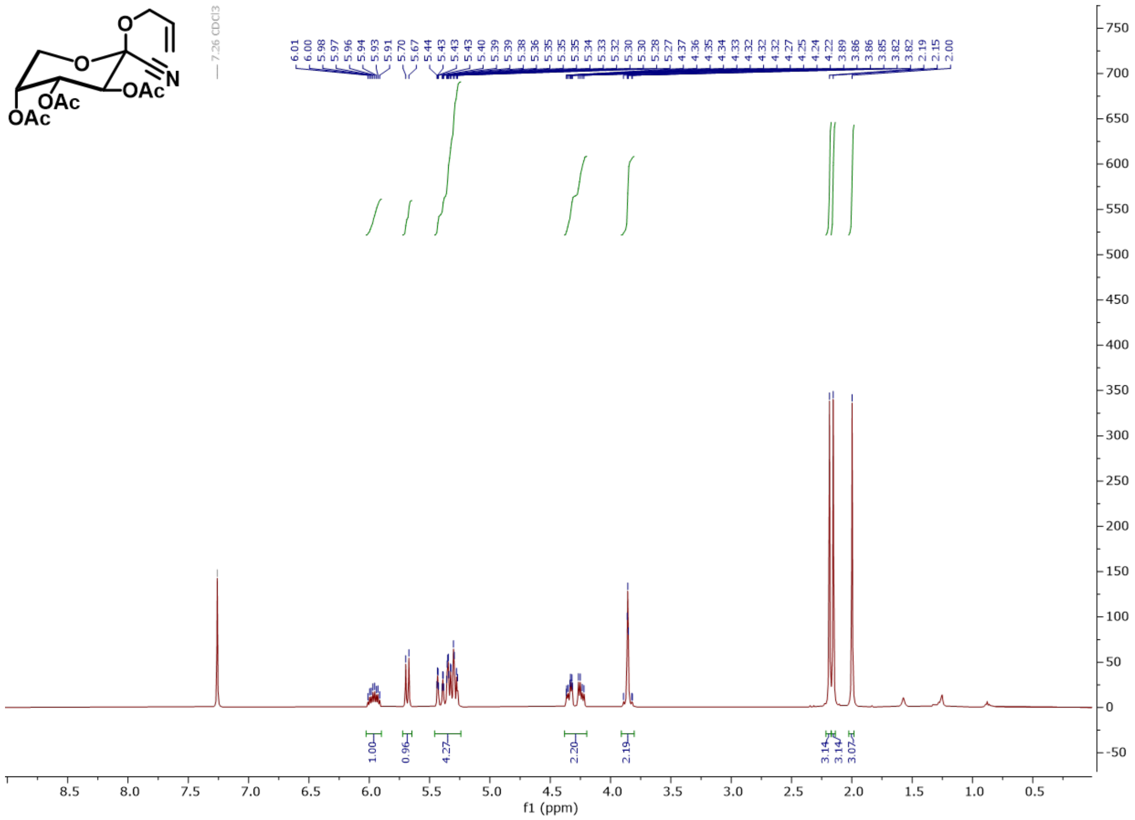

Figure S46: ^{1}H-NMR (400 MHz) spectrum in $CDCl_3$ of product **7g.**

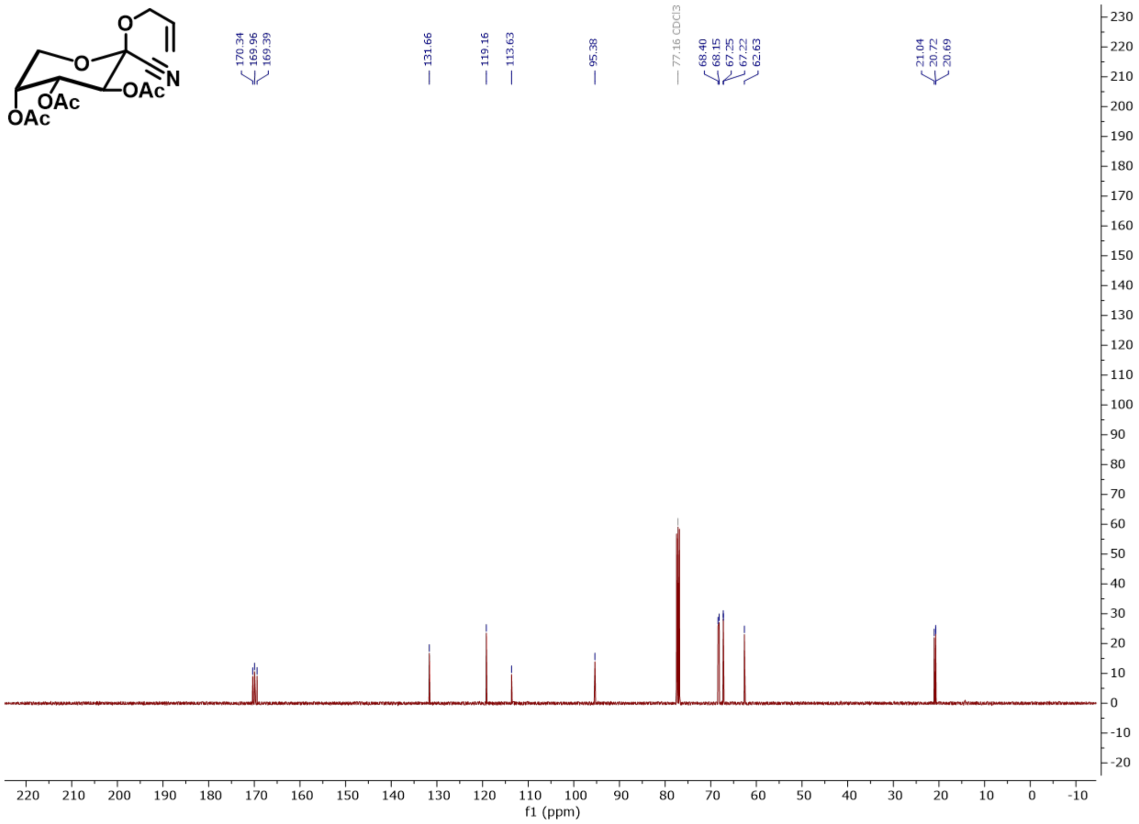

Figure S47: ^{13}C-NMR (101 MHz) spectrum in $CDCl_3$ of product **7g.**

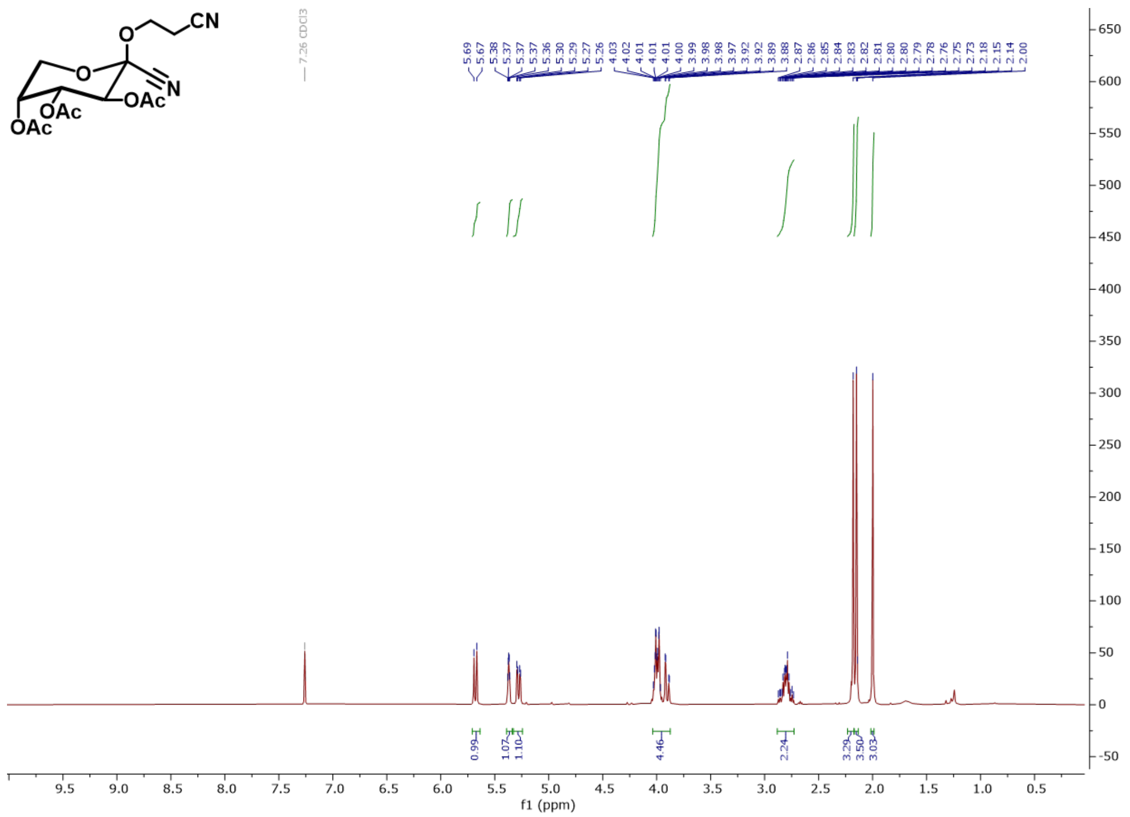

Figure S48: ^{1}H-NMR (400 MHz) spectrum in $CDCl_3$ of product **7h.**

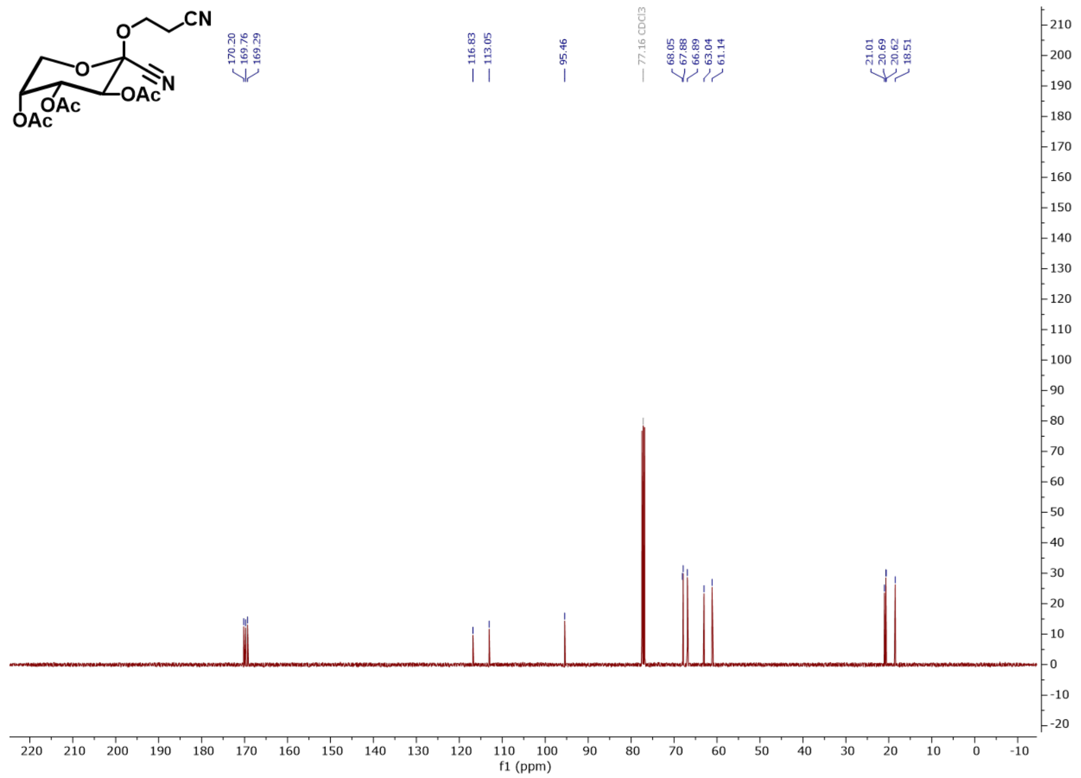

Figure S49: ^{13}C-NMR (101 MHz) spectrum in $CDCl_3$ of product **7h.**

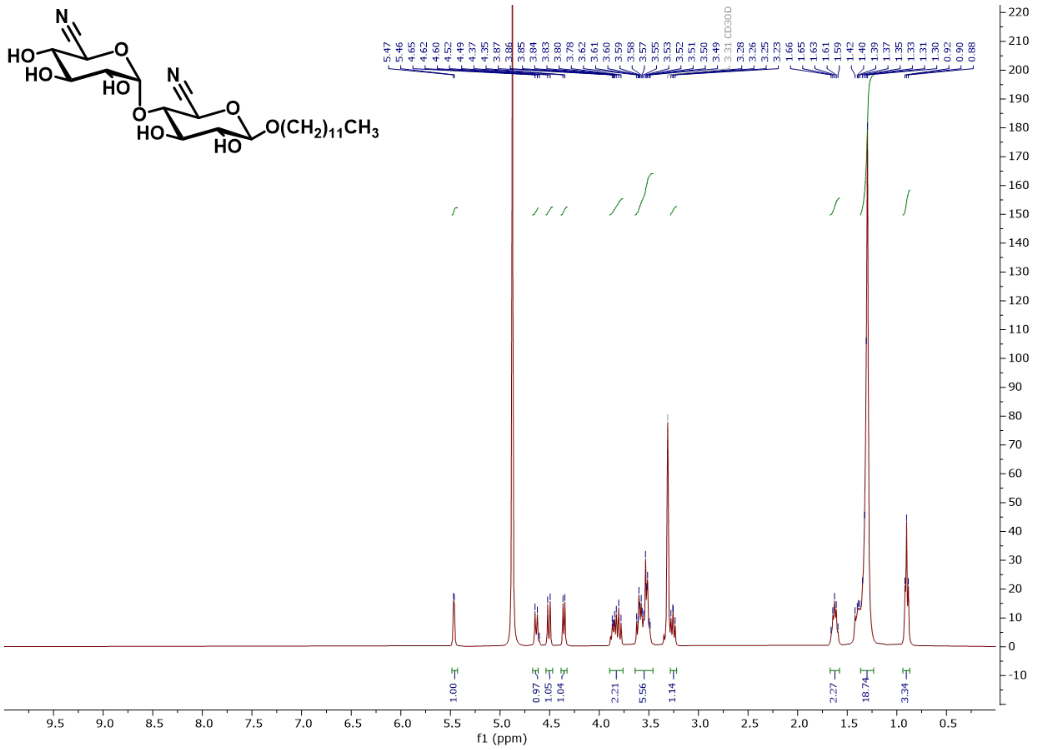

Figure S50*:* ^{1}H-NMR (400 MHz) spectrum in CD_3OD of product **4i.**

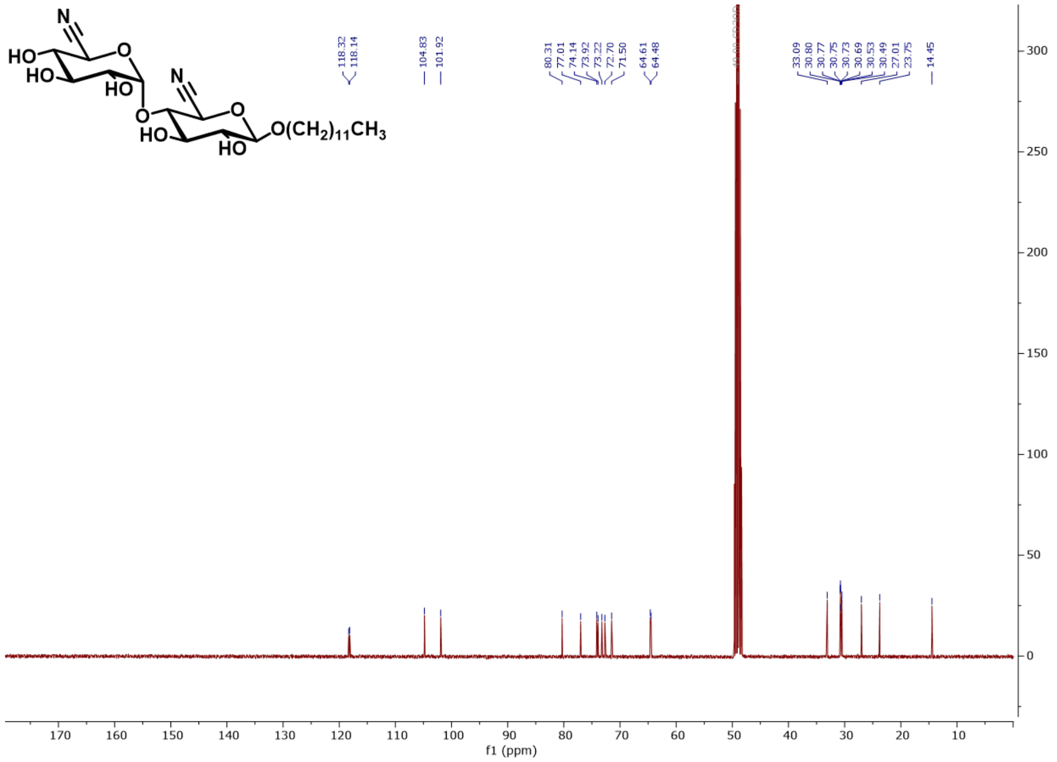

Figure S51: ^{13}C-NMR (101 MHz) spectrum in CD_3OD of product **4i.**

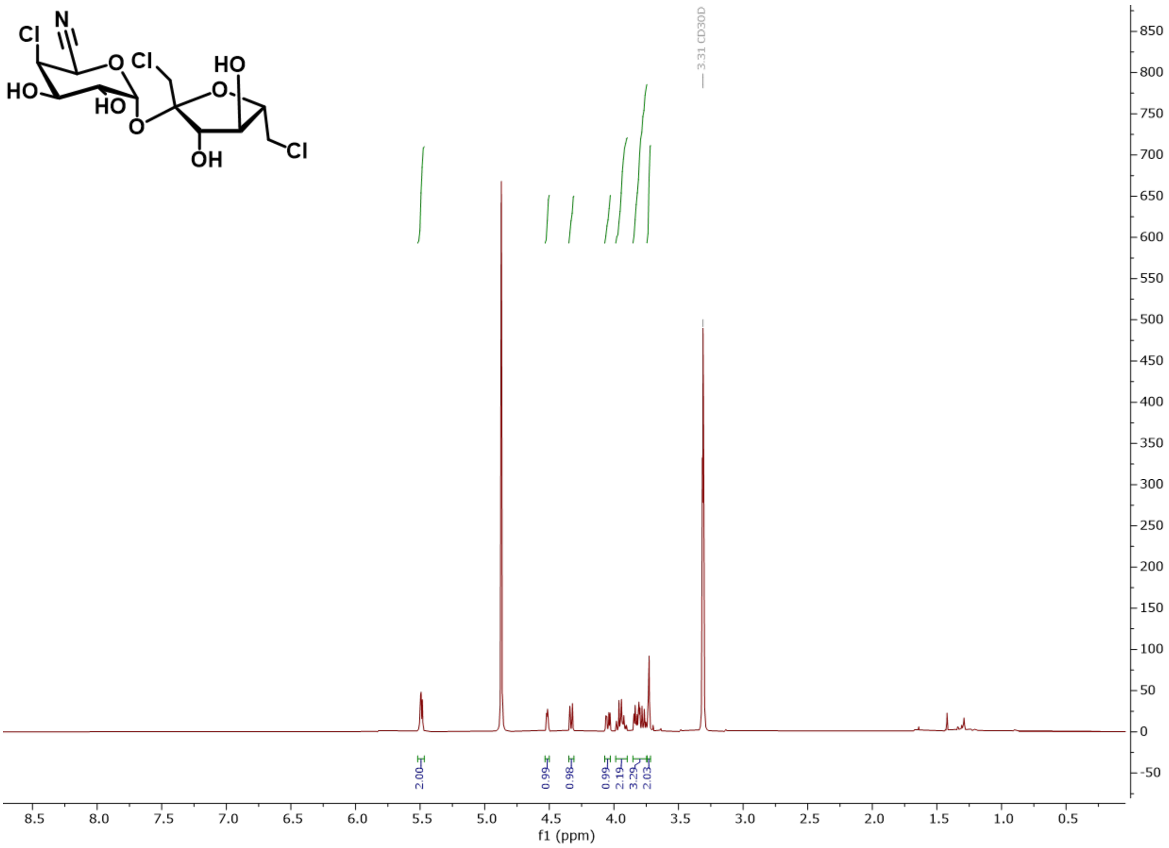

Figure S52: ^{1}H-NMR (400 MHz) spectrum in CD_3OD of product **4j.**

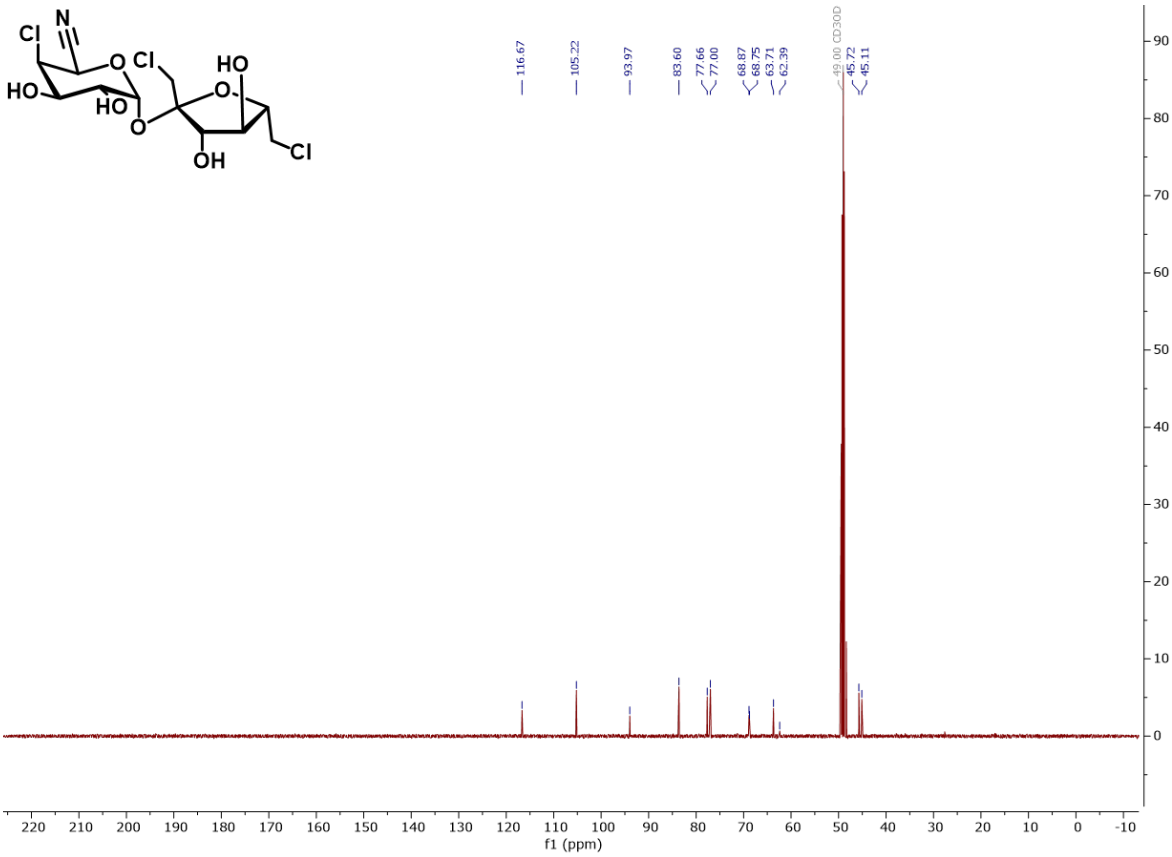

Figure S53*:* ^{13}C-NMR (101 MHz) spectrum in CD_3OD of product **4j.**

10.3 HRMS spectra

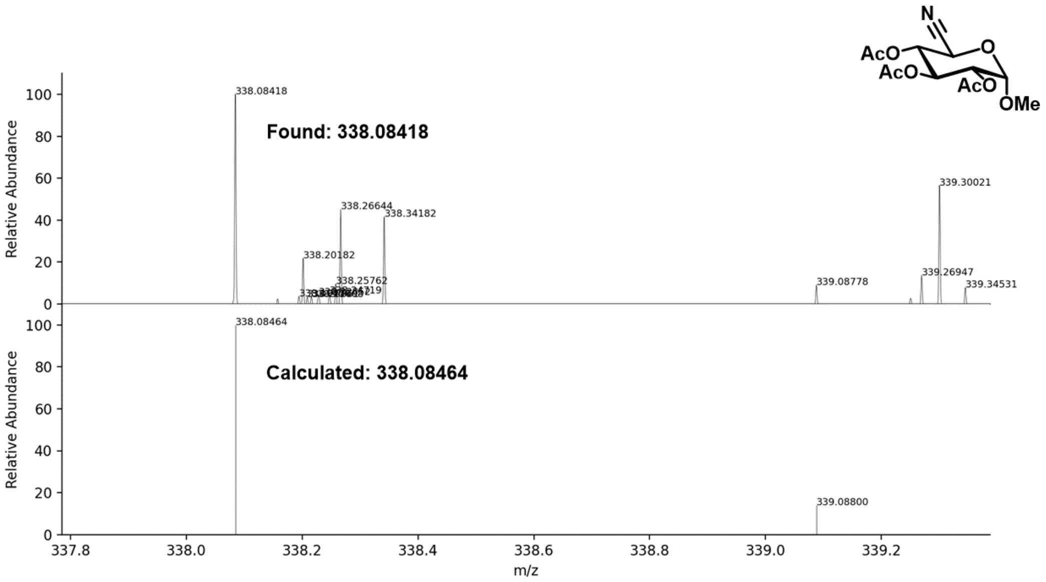

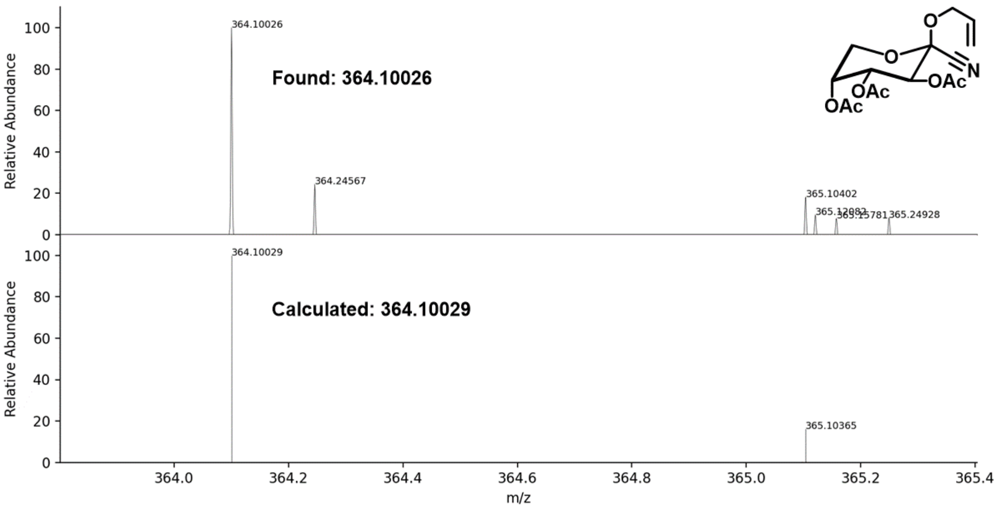

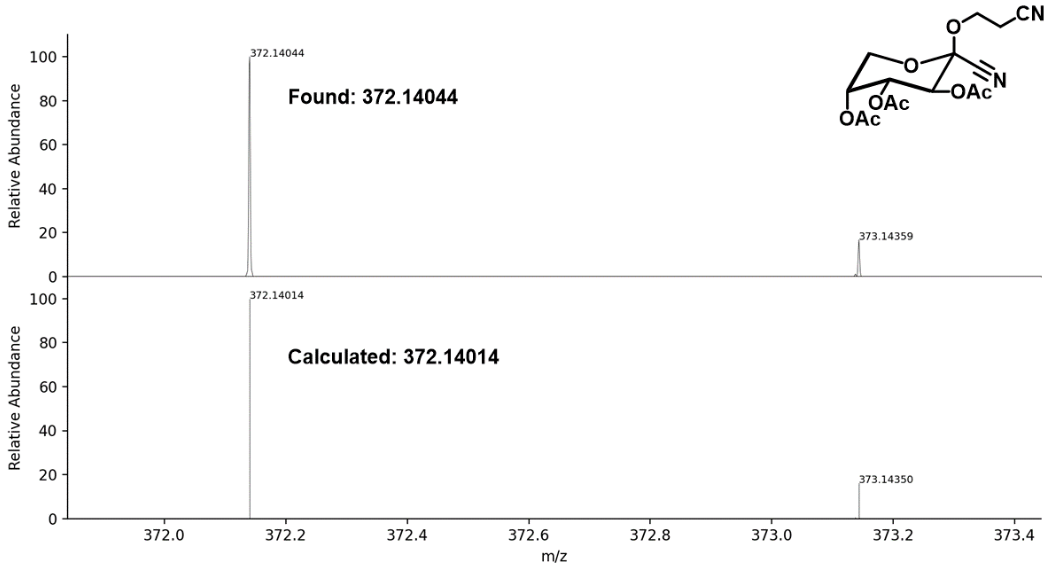
Found: 372.14044
Calculated: 372.14014
372.14044
373.14359
372.14014
373.14350
Relative Abundance
m/z

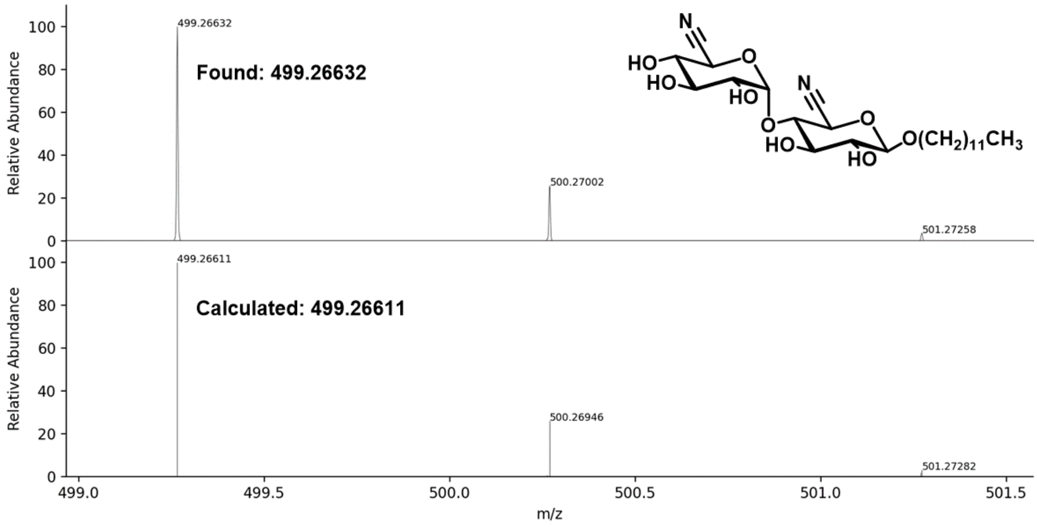
Found: 499.26632
Calculated: 499.26611
499.26632
500.27002
501.27258
499.26611
500.26946
501.27282
Relative Abundance
m/z

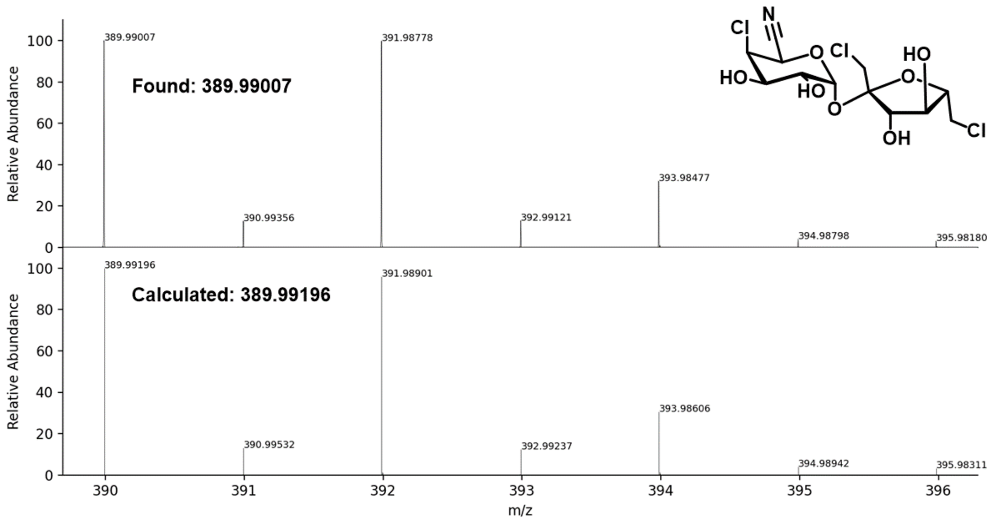
Found: 389.99007
Calculated: 389.99196
Relative Abundance
m/z
389.99007
390.99356
391.98778
392.99121
393.98477
394.98798
395.98180
389.99196
390.99532
391.98901
392.99237
393.98606
394.98942
395.98311

References

(1) Griffin, J. D.; Harper, K. C.; Velasquez Morales, S.; Morrill, W. H.; Thornton, W. I.; Sutherland, D.; Greiner, B. A. A Scalable Solution to Constant-Potential Flow Electrochemistry. *Org Process Res Dev* **2024**, *28* (5), 1877–1885. https://doi.org/10.1021/ACS.OPRD.3C00432.

(2) Sletten, E. M.; Liotta, L. J. A Flexible Stereospecific Synthesis of Polyhydroxylated Pyrrolizidines from Commercially Available Pyranosides. *Journal of Organic Chemistry* **2006**, *71* (4), 1335–1343. https://doi.org/10.1021/JO051792O/SUPPL_FILE/JO051792OSI20051205_102825.PDF.

(3) Haaksma, J.-J.; Kaniraj, J. P.; Opielak, W. M.; Egmond, J. van; Roo, C. M. de; Browne, W. R.; Minnaard, A. J.; Witte, M. D. Ammoxidation of Unprotected Glycosides: A One-Pot Conversion of Alcohols to Nitriles. *Chemistry – A European Journal* **2025**, e202500796. https://doi.org/10.1002/CHEM.202500796.

(4) Raaijmakers, H. W. C.; Arnouts, E. G.; Zwanenburg, B.; Chittenden, G. J. F. Some Aspects of the Synthesis of Allyl β-d-Fructopyranoside. *Carbohydr Res* **1994**, *257* (2), 293–297. https://doi.org/10.1016/0008-6215(94)80042-1.

(5) Lin, H. C.; Kidonakis, M.; Kaniraj, J. P.; Kholomieiev, I.; Fridrich, B.; Stuart, M. C. A.; Minnaard, A. J. The Synthesis of Fructose-Based Surfactants. *Green Chemistry* **2024**, *26* (8), 4715–4722. https://doi.org/10.1039/D4GC00399C.
